# Chemoselective Decarboxylative Oxygenation of Carboxylic Acids To Access Ketones, Aldehydes, and Peroxides

**DOI:** 10.1021/acs.orglett.3c00649

**Published:** 2023-04-04

**Authors:** Renpeng Guan, Guanhong Chen, Elliot L. Bennett, Zhiliang Huang, Jianliang Xiao

**Affiliations:** † Department of Chemistry, University of Liverpool, Liverpool L69 7ZD, United Kingdom; ‡ Hubei Biomass-Resource Chemistry and Environmental Biotechnology Key Laboratory, School of Resource and Environmental Sciences, Wuhan University, Wuhan, Hubei 430079, People’s Republic of China

## Abstract

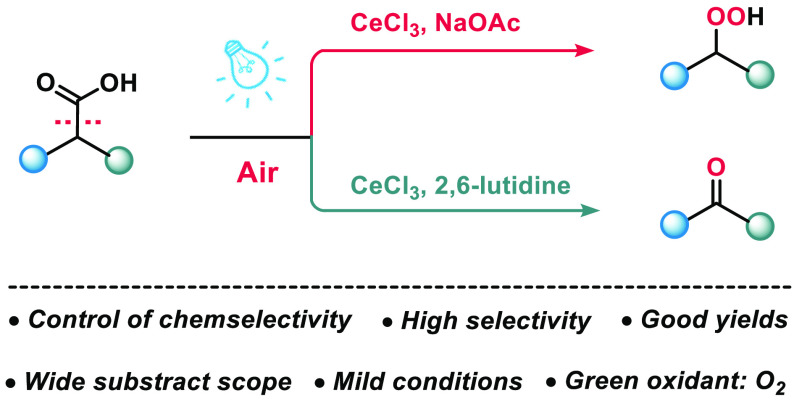

Reported here is a photocatalytic strategy for the chemoselective decarboxylative oxygenation of carboxylic acids using Ce(III) catalysts and O_2_ as the oxidant. By simply changing the base employed, we demonstrate that the selectivity of the reaction can be channeled to favor hydroperoxides or carbonyls, with each class of products obtained in good to excellent yields and high selectivity. Notably, valuable ketones, aldehydes, and peroxides are produced directly from readily available carboxylic acid without additional steps.

Control of chemoselectivity is one of the most important and enduring topics in organic synthesis.^1^ A case in point is decarboxylative oxygenation of carboxylic acids, which could afford two different products, a carbonyl and a peroxide, in a dehomologation manner (Scheme 1a). This is potentially a tremendously interesting reaction because of the easy availability of the substrate and the huge importance of each product. Carboxylic acids are probably the most easily accessible functionality in biological and chemical synthesis.^2^ Many of them are widespread in nature, e.g., amino acids, fatty acids, and keto acids, or are produced at a large industrial scale, e.g., formic acid, acetic acid, benzoic acid, and acrylic acid, and they are easy to store and simple to handle.^3−6^ The importance of ketones and aldehydes can hardly be overstated. They are widely used as precursors and starting materials in the synthesis of a wide variety of chemicals, including vitamins, drugs, and fragrances.^7^ In comparison, organic peroxides are less featured in organic synthesis. However, they play important roles in many biological processes, e.g., biodegradation and aging, and in drug development, e.g., as antimalarial agents, and they feature widely in oxidation reactions as oxidants and in polymerization processes as initiators.^8,9^

**Scheme 1:**
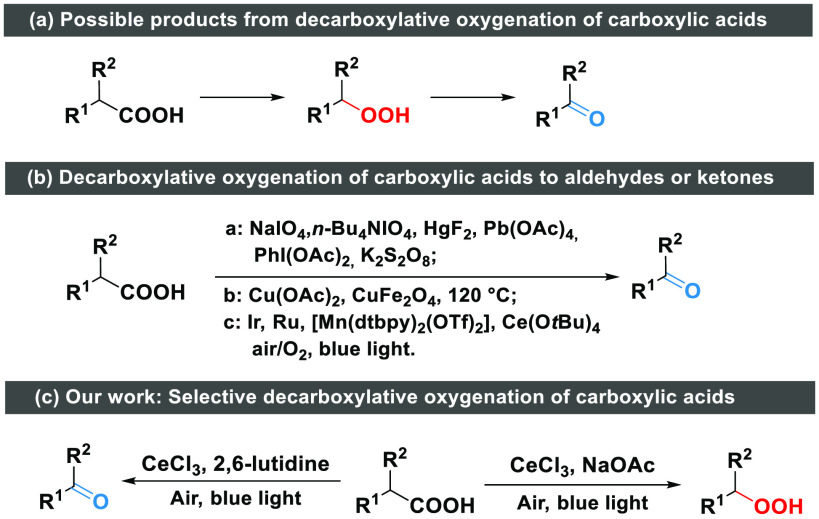
Decarboxylative Oxygenation of Carboxylic Acids To Form Various Products

Thus, developing a method for selective decarboxylative oxygenation of carboxylic acids to carbonyls and peroxides is of significant practical value. The transformation of carboxylic acids to aldehydes or ketones has been well-documented (Scheme 1b). Earlier methods often rely on the use of stoichiometric amounts of oxidants, such as NaIO_4_,^10^*n*-Bu_4_NIO_4_,^11^ HgF_2_,^12^ Pb(OAc)_4_,^13^ PhI(OAc)_2_,^14^ and K_2_S_2_O_8_,^15^ or a high temperature. Photocatalytic oxidative decarboxylation of carboxylic acids with O_2_ has recently been reported, using catalysts, such as acridiniums, [Ir(F(Me)ppy)_2_(bpy)]PF_6_, [Ru(bpy)_3_]Cl_2_, [Mn(dtbpy)_2_(OTf)_2_], or Ce(O*t*Bu)_4_, under blue or visible light irradiation.^16−19^

While various methods exist for the synthesis of ketones and aldehydes via decarboxylation of carboxylic acids, much fewer methods have been developed to access organic peroxides, and those that have been reported usually suffer from a limited substrate scope and/or rely on harsh conditions.^20^ For instance, there appears to be few methods that are feasible for the formation of both benzylic and aliphatic peroxides using benign and economic oxidants, i.e., O_2_^21^ or H_2_O_2_.^22^ Indeed, although a lot of work has been reported on oxidative decarboxylation^4,23^ and peroxides are generally believed to be a key intermediate in the reaction,^16,19,24^ only one example has shown the possibility of synthesis of peroxides from decarboxylation and their use in intramolecular cyclization.^19^

In continuing our interest in selective oxidation,^18,25−28^ we report herein a photocatalytic method that enables selective formation of aldehydes/ketones and peroxides, via aerobic decarboxylative oxygenation of carboxylic acids with simple, cheap cerium halides as a catalyst (Scheme 1c). Remarkably, the selectivity of the reaction can be tuned by a simple change of the base used.

We started by searching for conditions that would allow for selective decarboxylative oxygenation of carboxylic acids. Inspired by the remarkable ability of Ce(III/IV) in engaging photoredox reactions,^29−32^ at the outset, we examined CeCl_3_ as a potential catalyst, which is much cheaper and more easily available than Ce(O*t*Bu)_4_,^19^ for the model reaction of α-methylphenylacetic acid with 1 bar of air under the irradiation of blue light (465 nm and 9 W). The results are shown in Table 1. As seen, in the presence of 1 equiv of a base, NaOAc, hydroperoxide **1** was obtained, much to our surprise, in an excellent yield of 94%. The formation of hydroperoxides has been observed before but in a significantly lower yield.^19^ Interestingly, without the addition of the base, α-methylphenylacetic acid was transformed to a mixture of compound **1** and 1-phenylethanone (**23**) in 49 and 22% yield, respectively (entry 2).

**Table 1:**
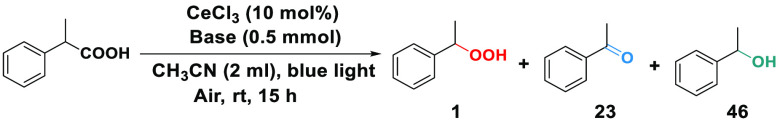
Optimization of Selective Transformation of Carboxylic Acids^a^

			yield (%)^b^		
entry	catalyst	base	**1**	**23**	**46**
1	CeCl_3_	NaOAc	94	2	0
2	CeCl_3_		49	22	0
3	CeCl_3_	2,6-lutidine	0	74	22
4	CeCl_3_	KOAc	37	8	0
5	CeCl_3_	LiOAc	38	42	0
6	CeCl_3_	CsOAc	33	9	0
7	CeCl_3_	Na_2_CO_3_	37	2	0
8	CeCl_3_	NaOH	9	1	0
9	CeCl_3_	pyridine	0	51	10
10	CeCl_3_	Et_3_N	0	37	0
11	CeCl_3_	DBU	0	10	0
12^c^	CeCl_3_	NaOAc	0	0	0
13		NaOAc	0	0	0
14^d^	CeCl_3_	NaOAc	0	0	0

a Reaction conditions: α-methylphenylacetic acid (0.5 mmol), CeCl_3_ (10 mol %), base (0.5 mmol), CH_3_CN (2 mL), air, blue light (465 nm and 9 W), room temperature, and 15 h.

b NMR yields are given, determined using mesitylene (20 μL) as the internal standard.

c Reaction in the dark.

d N_2_ instead of air.

Aiming to alter the reaction selectivity, we screened a range of bases in the reaction. As is clear, the base plays a decisive role in affecting the selectivity of products (entries 1 and 3–11). While NaOAc led to almost exclusive formation of peroxide **1**, replacing it with 2,6-lutidine afforded ketone **23** and alcohol **46** in yields similar to those obtained with Ce(O*t*Bu)_4_.^19^ Lower yields were observed with other bases, such as Na_2_CO_3_, NaOH, KOAc, CsOAc, LiOAc, Et_3_N, and pyridine (entries 4–11). It is interesting to note that the formation of the peroxide is suppressed by amine bases but strongly promoted by NaOAc and to a lesser degree by Na_2_CO_3_. The difference in yield observed with the different acetate bases (entries 1 and 4–6) may be at least partly due to their varying solubilities in the solvent used (Table S2 of the Supporting Information). This dramatic effect of bases on the chemoselectivity of decarboxylative oxygenation has not been noted in previous studies. Taking NaOAc and 2,6-lutidine as the optimum base for the formation of compounds **1** and **23**, respectively, we also examined the effect of other cerium compounds as possible catalysts (Table S1 of the Supporting Information). As may be expected, blue light, CeCl_3_, and air are all essential components for the decarboxylative oxygenation to occur (entries 12–14).

To demonstrate the generality of our strategy, we investigated the decarboxylative oxygenation of a variety of carboxylic acids. First, the scope for the formation of hydroperoxides was examined. As shown in Scheme 2, a variety of phenylacetic acids underwent selective decarboxylative oxygenation, affording the corresponding hydroperoxide products in good yields (46–94%). All of the halogen-substituted (*p*-CF_3_, *p*-Br, *p*-Cl, and *p*-F) phenylacetic acids were tolerated; they afforded the corresponding hydroperoxide products (**4**–**7**) in good yields. Thiopheneacetic acid also worked, without poisoning the catalyst, as did 2-naphthylacetic acid, albeit in moderate yields. The position of substitutes affects the yields, as *m*-substituted (**9**) and *o*-substituted (**10**) hydroperoxide products showed lower yields (61 and 59%). This might result from some steric hindrance.^33^ It is worth noting that the secondary (**1**) and tertiary (**11**) peroxides were obtained in significantly higher yields (94%) than the primary analogue (**2**), indicating the involvement of a benzylic radical in the formation of the peroxide products. The reaction could also be run at a larger scale, albeit with a reduced yield (**1**).

**Scheme 2:**
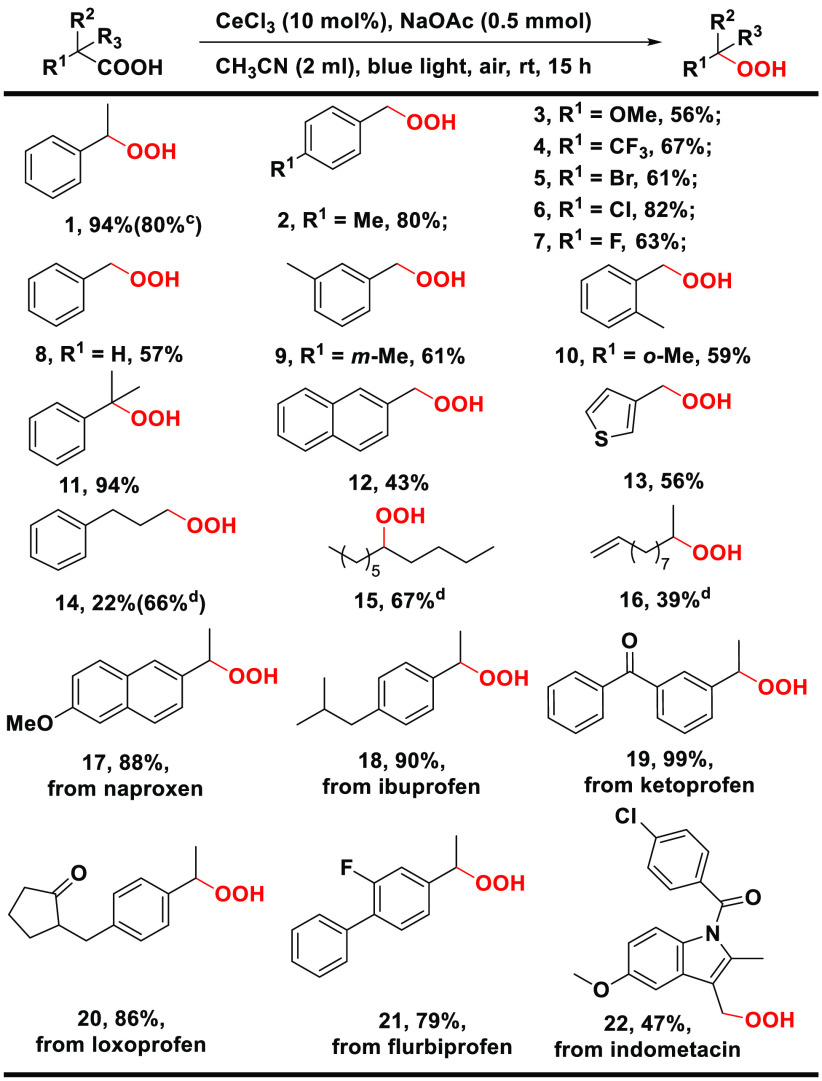
Decarboxylative Oxygenation of Carboxylic Acids to Hydroperoxides^,^ Reaction conditions: acid (0.5 mmol), CeCl_3_ (10 mol %), NaOAc (0.5 mmol), CH_3_CN (2 mL), blue light (465 nm and 9 W), air, room temperature, and 15 h. Isolated yields are given. Acid (1 mmol). Na_2_CO_3_ (0.5 mmol) instead of NaOAc and a N_2_/O_2_ (1:2) mixture instead of air.

The more challenging aliphatic acids are also feasible, as showcased by peroxides **14**–**16**; however, a higher O_2_ concentration (N_2_/O_2_ volume of 1:2 and 1 bar) and Na_2_CO_3_ as the base were necessary. As shown in Scheme 2, a lower yield of compound **14** was obtained under the condition of using air and NaOAc. Remarkably, the oxidation-prone C=C bond remained intact in compound **16**, and byproducts were formed in very low yields (eq 1 in Scheme S1 of the Supporting Information). There was no benzylic oxidation in compound **14**.

Furthermore, a series of anti-inflammatory drugs, such as naproxen, ibuprofen, ketoprofen, loxoprofen, flurbiprofen, and indomethacin, could be oxidatively decarboxylated, furnishing the corresponding hydroperoxide products (**17**–**22**) in moderate to high yields (47–99%). Such peroxides could provide metabolites for a drug study, because they may form under enzymatic oxidation.^34^ We note that, while the formation of peroxyl species from the reaction of the carbon radical with triplet O_2_ is generally assumed,^16,19,24,35^ this is the first time a range of peroxides have been isolated as potentially useful products in oxidative decarboxylation.

A simple change of the base from NaOAc to 2,6-lutidine allows for the selectivity of the oxidative decarboxylation to be channeled to carbonyl products. The scope of aldehydes and ketones resulting from the selective decarboxylative oxygenation of acids is shown in Scheme 3, demonstrating the adaptability and practicability of the method. As seen, a variety of phenylacetic acids bearing different functional groups were converted to the corresponding aldehyde and ketone products (**23**–**35**) in moderate to good yields (43–75%). *o*-Tolylacetic acid was selected as the example substrate to showcase the chemoselectivity of this transformation. As shown in Scheme S1 of the Supporting Information, only a trace of alcohol byproduct was detected. Phenylacetic acids bearing electron-withdrawing halide substituents, including −CF_3_, −Br, −Cl, and −F, were tolerated in the decarboxylative oxygenation, as were those bearing electron-donating substitutes, e.g., *m*-Me and *o*-Me (**34** and **35**). Moreover, an acid bearing a heteroatom ring, i.e., thiophene, showed good reactivity, affording compound **36** in a good yield (70%). Interestingly, 2-(phenylmethoxy)acetic acid with an oxygen atom in the carbon chain also reacted smoothly, without the weak benzylic C–H bond being compromised (**37**). An amino acid derivative was also tolerated, giving the corresponding amide product **38** in a good yield (66%).

**Scheme 3:**
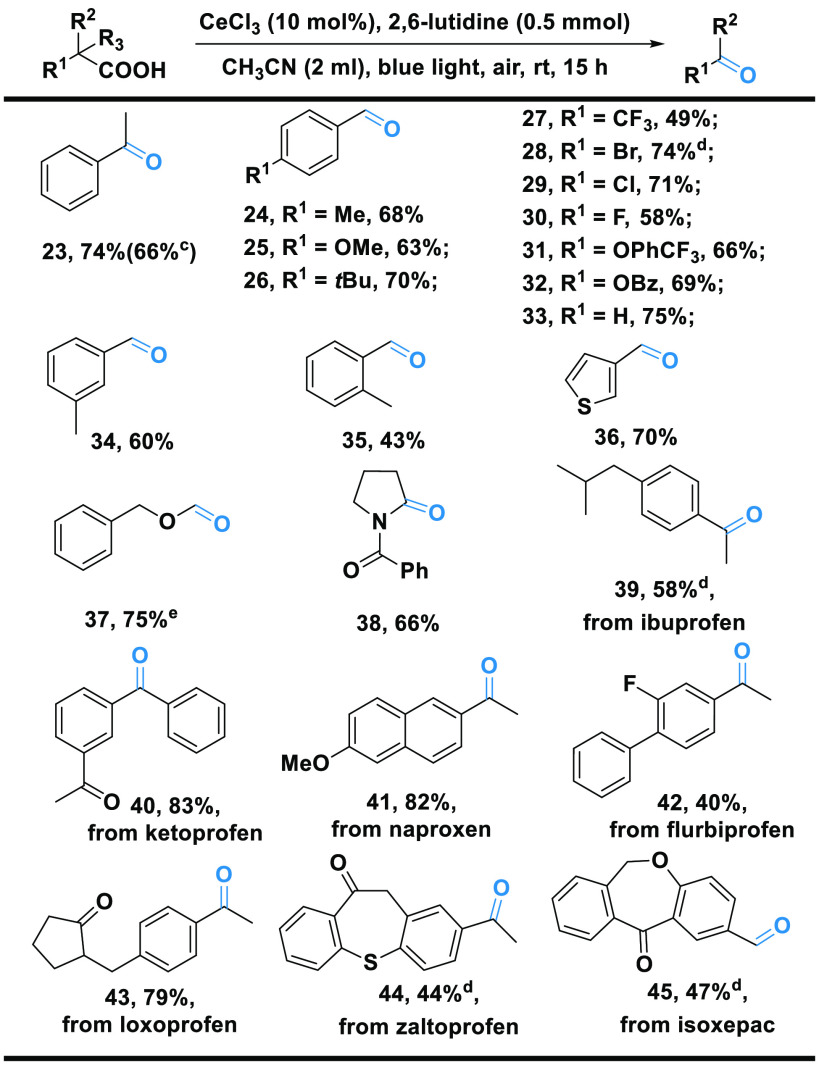
Decarboxylative Oxygenation of Carboxylic Acids to Aldehydes and Ketones^,^ Reaction conditions: acid (0.5 mmol), CeCl_3_ (10 mol %), 2,6-lutidine (0.5 mmol), CH_3_CN (2 mL), blue light (465 nm and 9 W), air, room temperature, and 15 h. Isolated yields are given. Acid (1 mmol). Pyridine (0.5 mmol) instead of 2,6-lutidine. UV (365 nm and 9 W) instead of blue light.

As with the reaction leading to peroxides, a wide range of drug molecules, including ibuprofen, ketoprofen, naproxen, flurbiprofen, loxoprofen, zaltoprofen, and isoxepac, underwent decarboxylative oxygenation to yield the corresponding aldehyde or ketone products (**39**–**45**) in moderate to excellent yields (40–83%). Apart from the possible use in the study of drug metabolism, these derivatives may serve as useful scaffolds to build new bioactive molecules or as substrates for further reactions.^36,37^

While the mechanism of decarboxylative oxygenation of carboxylic acids has been widely accepted,^17−19^ the chemoselective formation of isolable peroxides, aldehydes, and ketones prompted us to look into the mechanism concerning particularly what controls the selectivity of the reaction. First, the coordination of carboxylic acids with cerium catalysts was explored by mass spectroscopy with phenylacetic acid (PA) as a standard substrate and CeCl_3_ as a catalyst. As shown in Figure S3 of the Supporting Information, mixing CeCl_3_ with PA appears to lead to, as indicated by high-resolution mass spectrometry (HRMS) measurement, a cerium species [Ce(PA–H)_2_]^+^, which could result from the coordination of two PA molecules with a Ce(III) center.^38,39^ It is thus likely that the selective decarboxylative oxygenation starts from the coordination of carboxylic acids to CeCl_3_. Indeed, esters do not engage in the reaction (Scheme S2 of the Supporting Information).

As with other decarboxylative oxygenation reactions,^19^ alkyl hydroperoxides are likely to be a key intermediate. The kinetic profile of the reaction of α-methylphenylacetic acid reveals that this is the case. As seen in Figure S4a of the Supporting Information, the formation of hydroperoxide **1** is rapid and precedes that of ketone **23** and alcohol **46**, and its decrease is coincided with the rise of the latter two. Furthermore, subjecting isolated compound **1** to the conditions of CeCl_3_, 2,6-lutidine, and blue light afforded compound **23** in 78% yield (Figure S4b of the Supporting Information). It is thus reasonable to conclude that the carbonyl products result from the peroxide intermediate.

The question then is why are the peroxides not reacting further, as is usually observed? Table 1 indicates that the base plays a critical role. This is more clearly manifested when isolated peroxide **1** was subjected to blue light irradiation, in which compound **1** remained largely intact when using NaOAc as the base but fully converted to compounds **23** and **46** when 2,6-lutidine was used (eqs 1 and 2 in Scheme S3 of the Supporting Information). A possible explanation is that the acetate anion coordinates to cerium, preventing that of peroxide and, hence, its further transformation, whereas 2,6-lutidine could not play such a role. This conjecture finds support in ultraviolet–visible (UV–vis) experiments (see Figure S5b of the Supporting Information and related explanation).^40,41^

On the basis of the above observations and previous literature,^17−19^ a simplified mechanism of this selective decarboxylative oxygenation reaction is suggested (Scheme 4). First, a Ce(III) compound reacts with carboxylic acid, forming the complex **A**. Under light irradiation, complex **A** is oxidized by O_2_ to afford a Ce(IV) superoxide species **B**.^19^ Ce(IV) carboxylate is well-known to undergo facile decarboxylation via light-promoted homolysis of the Ce–oxygen bond. The resulting alkyl radical would be easily trapped by the superoxide radical, giving rise to a Ce(III) peroxide species **C**, of which metathesis with a free carboxylic acid then releases the observed alkyl hydroperoxide. However, light may not be necessary for the conversion of species **B** to species **C**, as indicated by the oxidation of α-methylphenylacetic acid in the dark with pre-irradiated CeBr_3_ mentioned above. The decarboxylation could be facilitated by the superoxide radical attacking α carbon, a process reminiscent of an iron-catalyzed oxidation of ethers.^42^ The peroxide product from species **C** is stable in the presence of NaOAc but is transformed to carbonyl or alcohol when using 2,6-lutidine as a base. Light is necessary to promote the single-electron reduction of O_2_ by Ce(III) species and the transformation of peroxide to aldehyde.^18,19^

**Scheme 4:**
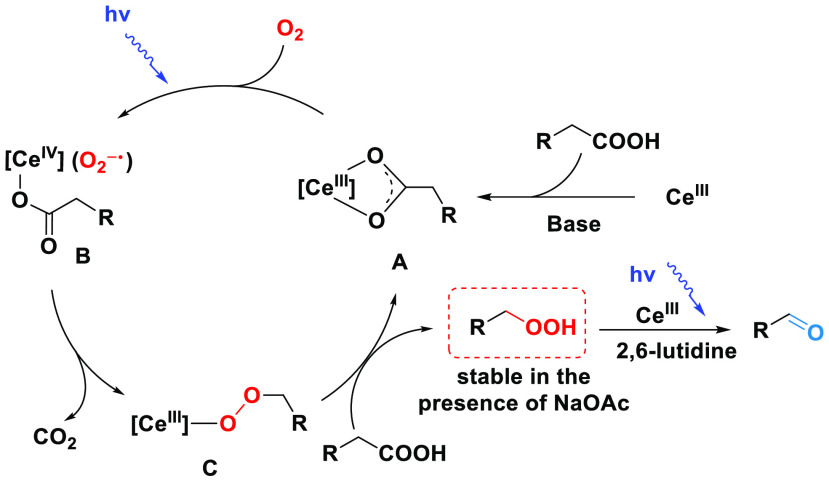
Proposed Mechanism of Selective Decarboxylative Oxygenation of Carboxylic Acids ([Ce^*n*^] Species)

In conclusion, a Ce(III)-catalyzed selective decarboxylative oxygenation of carboxylic acids to widely different products has been developed. The selectivity of this decarboxylative oxygenation process can be tuned with a simple change of the base. With this protocol, a wide range of carboxylic acids have been selectively transformed to hydroperoxides, aldehydes, and ketones in good yields with O_2_ under mild conditions.

## Data Availability

The data underlying this study are available in the published article and its Supporting Information.

## Supporting Information Available

General information, preparation of substrates, optimization of reaction conditions, and characterization data (PDF)
