# Supplementary material for: Chemoselective Decarboxylative Oxygenation of Carboxylic Acids To Access Ketones, Aldehydes, and Peroxides
Source: Org Lett. 2023 Apr 4;25(14):2482–6. doi: 10.1021/acs.orglett.3c00649 (PMC10111417; doi:10.1021/acs.orglett.3c00649)
Supplement: Supplementary file 1 — ol3c00649_si_001.pdf [file ol3c00649_si_001.pdf]

Chemoselective Decarboxylative Oxygenation of Carboxylic Acids to Access Ketones, Aldehydes and Peroxides

Renpeng Guan,[†] Guanhong Chen,[†] Elliot L. Bennett,[†] Zhiliang Huang*[,†,‡] and Jianliang Xiao*[†]

[†] Department of Chemistry, University of Liverpool, L69 7ZD, UK.

[‡] Hubei Biomass-Resource Chemistry and Environmental Biotechnology Key Laboratory, School of Resource and Environmental Sciences, Wuhan University, 430079 Wuhan, China.

E-mail: jxiao@liverpool.ac.uk; zlhuang@whu.edu.cn

Contents

1. General information 1

2. Information of photoreactors 2

3 Preparation of substrates 3

4 General procedure for selective decarboxylative oxygenation of carboxylic acids 4

5 Mechanistic studies 5

6 Analytical data of products 9

6.1 Hydroperoxide products 9

6.2 Aldehyde and ketone products 13

7 NMR spectra of substrates 19

8 NMR spectra of products 21

References 66

1. General information

All manipulations were carried out using standard Schlenk techniques. All glassware was dried at 100 °C for more than one hour before use. Cerium catalysts, different bases, and carboxylic acids were purchased from commercial suppliers and used without further purification. Unless otherwise noted, analytical grade solvents and commercially available reagents were used as received. Analytical thin layer chromatography (TLC) was conducted with TLC Silica gel 60 F254 (Merck) and plates were visualized under UV irradiation, potassium permanganate, or phosphomolybdic acid staining. Flash column chromatography was performed using Aldrich Silica Gel 60 and columns were packed according to the dry method and equilibrated with the appropriate eluent prior to use. HPLC grade solvents were used and the solvent mixtures used as eluent are given as volume/volume. All new compounds were characterized by ^{1}H NMR, ^{13}C NMR and HRMS. The known compounds were characterized by ^{1}H NMR and ^{13}C NMR. Data for ^{1}H NMR are reported as follows: chemical shift (δ ppm), multiplicity (s = singlet, d = doublet, t = triplet, q = quartet, m = multiplet), coupling constant (Hz), and integration. Data for ^{13}C NMR are reported in terms of chemical shift. The ^{1}H and ^{13}C NMR spectra were recorded on a Bruker Advance 400 NMR spectrometer at 400 MHz (^{1}H NMR) and 101 MHz (^{13}C NMR), respectively. The chemical shifts (δ) were given in parts per million relative to internal tetramethylsilane (0 ppm for ^{1}H), $CDCl_3$ (7.26 ppm for ^{1}H, 77.00 ppm for ^{13}C), or DMSO-d_6 (2.5 ppm for ^{1}H, 39.52 ppm for ^{13}C). Mass spectra were obtained by electrospray ionization (ESI) using Agilent

6540 UHD Accurate-Mass Q-TOF LC/MS at the Analytical Services of the Chemistry Department or Materials Innovation Factory, University of Liverpool.

2. Information of photoreactors

Information for the blue LEDs: 2.95 V blue LED SMD, Lumileds LUXEON Rebel LXML-PB01-0040; dominant wavelength or peak wavelength (minimum: 460 nm, typical: 465 nm, maximum: 485 nm); typical spectral half-width (20 nm); typical temperature coefficient of dominant or peak wavelength (0.05 nm/°C); typical total included angle (160°); typical view angle (125°). Each hole on the photoreactor is fitted with three LEDs, giving a total power for each reaction tube as 9 W. Reactor temperature after 12 h irradiation was 45 °C.

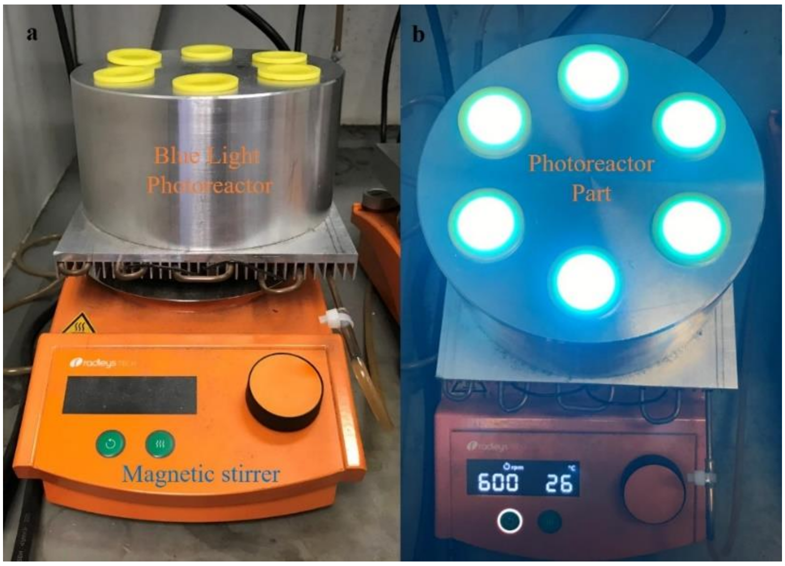

Figure S1. UV photoreactor.

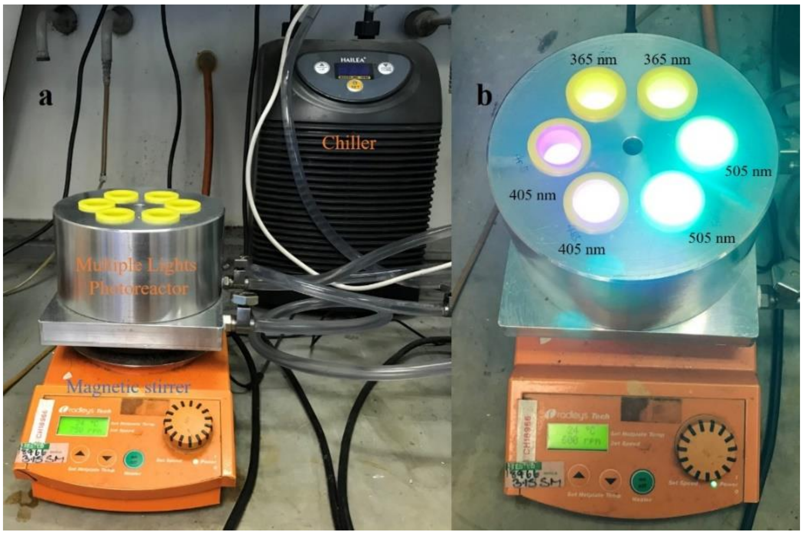

Figure S2. Multi-wavelength photoreactor.

Information for LEDs with various wavelengths:

UV LEDs (365 nm): 3.0 V UV LED SMD, LUMINUS SST-10-UV-E365-00; dominant wavelength or peak wavelength (minimum: 365 nm, typical: 365 nm, maximum: 370 nm); typical spectral half-width (10 nm); typical total included angle (160°); typical view angle (130°). Each hole on the photoreactor is

fitted with three LEDs, giving a total power for each reaction tube as 9 W. Temperature of this photoreactor was controlled by a cooler, and the reactor temperature was around 20 °C.

UV LEDs (405 nm): 3.0 V UV LED SMD, LUMINUS SST-10-UV-F405-00; dominant wavelength or peak wavelength (minimum: 405 nm, typical: 405 nm, maximum: 410 nm); typical spectral half-width (10 nm); typical total included angle (160°); typical view angle (130°). Each hole on the photoreactor is fitted with three LEDs, giving a total power for each reaction tube as 9 W.

Cyan LEDs (505 nm): 2.90 V cyan LED SMD, Lumileds LUXEON Rebel LXML-PE01-0080; dominant wavelength or peak wavelength (minimum: 495 nm, typical: 505 nm, maximum: 515 nm); typical spectral half-width (30 nm); typical temperature coefficient of dominant or peak wavelength (0.04 nm/°C); typical total included angle (160°); typical view angle (125°). Each hole on the photoreactor is fitted with three LEDs, giving a total power for each reaction tube as 9 W.

3 Preparation of substrates

3.1 Synthesis of 2-methylundec-10-enoic acid

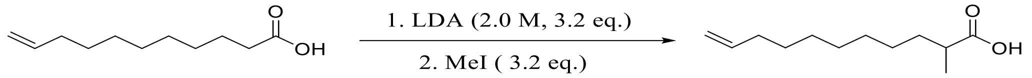

2-Methylundec-10-enoic acid was synthesized according to the literature.[1] Under an argon atmosphere, undec-10-enoic acid (10 mmol, 1.0 eq.) in dry THF (10 mL) was added slowly (over 20 min) into a LDA solution in a Schlenk tube cooled with an ice-water bath. After that, the reaction mixture was warmed to 45 °C, stirred for 3 h, and cooled to -78 °C. Then, MeI (32 mmol, 3.2 eq.) was added into the solution dropwise, and the resulting mixture was warmed to room temperature and stirred overnight. The reaction mixture was quenched by adding water (80 mL) slowly, followed by adding 2.0 M NaOH (aq.) to adjust the pH to 14. The resulting mixture was extracted with diethyl ether (50 mL × 2) and DCM (50 mL × 2). Then, 3.0 M HCl (aq.) was added into the aqueous phase to adjust the pH to 1-2 and the mixture was extracted with EtOAc (100 mL). Finally, the organic layer was dried over Na_2SO_4 and concentrated to obtain 2-methylundec-10-enoic acid as a colourless oil (1.20g, 65%).

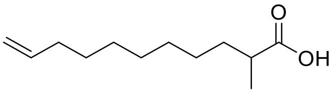

2-Methylundec-10-enoic acid[2]
^{1}H NMR (400 MHz, $CDCl_3$) δ 5.86-5.76 (m, 1H), 5.01-4.92 (m, 2H), 2.50-2.41 (m, 1H), 2.06-2.01 (m, 2H), 1.46 – 1.12 (m, 15H).
^{13}C NMR (101 MHz, $CDCl_3$) δ 183.03, 139.17, 114.13, 39.31, 33.77, 33.51, 29.42, 29.28, 29.04, 28.88, 27.10, 16.82.

3.2 Synthesis of *N*-benzoyl-*l*-proline and 12-benzamidododecanoic acid

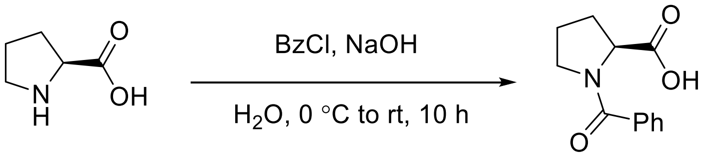

N-Benzoyl-*l*-proline was synthesized according to the literature.[3] To a solution of *L*-proline (4.00 g, 34.7 mmol) and NaOH (2.78 g, 69.5 mmol) in H_2O (64 mL), benzoyl chloride (BzCl, 4.0 mL, 34.7 mmol) was added dropwise at 0 °C. The mixture was stirred for 10 h at room temperature. The mixture was diluted with H_2O and washed with Et_2O (× 1). The aqueous layer was acidified with 10% HCl to pH = 1 and extracted with ethyl acetate. The organic layer was dried over $MgSO_4$ and evaporated to afford *N*-benzoyl-*l*-proline as colourless crystals (3.43 g, 86%).

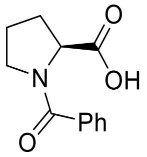

***N*-Benzoyl-*l*-proline**[3]

^{1}H NMR (400 MHz, $CDCl_3$) δ 10.07 (s, 1H), 7.59-7.55 (m, 2H), 7.46-7.40 (m, 3H), 4.76 (t, *J* = 6.0 Hz, 1H), 3.58 (m, 2H), 2.35-2.23 (m, 2H), 2.09-1.98 (m, 1H), 1.95-1.85 (m, 1H).

^{13}C NMR (101 MHz, $CDCl_3$) δ 174.85, 170.86, 135.38, 130.42, 128.27, 127.18, 59.53, 50.23, 28.76, 25.11.

4 General procedure for selective decarboxylative oxygenation of carboxylic acids

4.1 Screening of experimental conditions for decarboxylative oxygenation of carboxylic acids

To an oven dried Schlenk tube, a Ce catalyst (10 mol%), base (0.5 mmol), α-methylphenylacetic acid (0.5 mmol) and 2 mL of CH_3CN were added under air. The Schlenk tube was allowed to stir under blue light (465 nm) for 15 h. After the reaction, water (2 mL), chloroform-*d* (1 mL), and mesitylene (17.3 mg) as internal standard were added. The product yield of **1, 23** and **46** was obtained by ^{1}H NMR analysis of the organic layer.

Table S1. Screening of Ce catalysts for selective decarboxylative oxygenation of α-methylphenylacetic acid.[a, b]

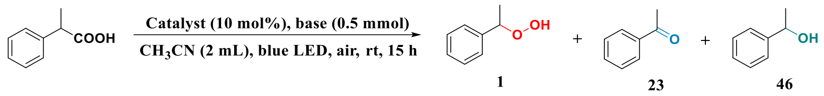

Entry	Catalyst	Base	Yield (%)		
			**1**	**23**	**46**
1	$Ce(OAc)_3$	NaOAc	88	2	0
2	$CeBr_3$	NaOAc	89	4	0
3	$Ce_2(C_2O_4)_3$	NaOAc	90	1	0
4	CeF_3	NaOAc	11	0	0
5	CeF_4	NaOAc	50	1	0
6	$NH_4Ce(NO_3)_6$	NaOAc	84	1	0
7	$Ce(OAc)_3$	2,6-lutidine	0	51	28
8	$CeBr_3$	2,6-lutidine	0	36	17
9	$Ce_2(C_2O_4)_3$	2,6-lutidine	0	2	0
10	CeF_3	2,6-lutidine	0	6	0
11	CeF_4	2,6-lutidine	0	0	3
12	$NH_4Ce(NO_3)_6$	2,6-lutidine	0	0	5

[a]Reaction conditions: α-methylphenylacetic acid (0.5 mmol), catalyst (10 mol%), base (0.5 mmol), CH_3CN (2 mL), under air, blue LED (465 nm, 9W), room temperature, overnight. [b]NMR yields are given.

4.2 Detection of byproducts in selective decarboxylative oxygenation

2-Methylundec-10-enoic acid: To an oven dried Schlenk tube, $CeCl_3$ (10 mol%), 2-methylundec-10-enoic acid (0.5 mmol), NaOAc (0.5 mmol) and CH_3CN (2 ml) were added under air. After that, the reaction tube was allowed to stir under blue light irradiation (465 nm) for 15 h. After the reaction, water (2 mL), chloroform-*d* (1 mL), and mesitylene (17.3 mg) were added. The product yields were obtained by ^{1}H NMR analysis of the organic layer.

o-Tolylacetic acid: To an oven dried Schlenk tube, $CeCl_3$ (10 mol%), *o*-tolylacetic acid (0.5 mmol), 2,6-lutidine (0.5 mmol) and CH_3CN (2 ml) were added under air. After that, the reaction tube was allowed to stir under blue light irradiation (465 nm) for 15 h. After the reaction, water (2 mL),

chloroform-*d* (1 mL), and mesitylene (17.3 mg) were added. The product yields were obtained by ^{1}H NMR analysis of the organic layer.

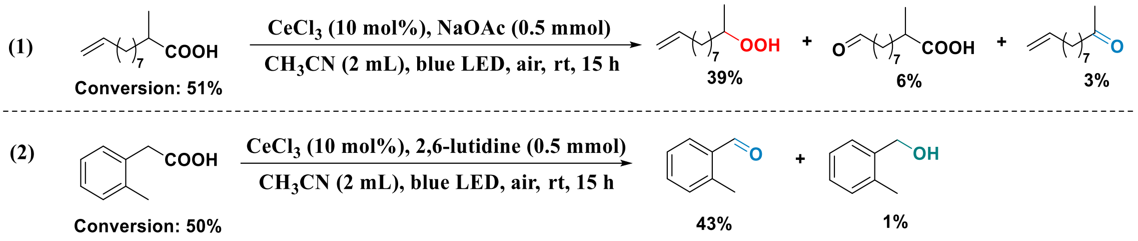

Scheme S1. Selective decarboxylative oxygenation of different acids.

4.3 Estimation of solubility of base in selective decarboxylative oxygenation

To a sample tube (14 mL), NaOAc (500 mg) was added. The total weight of tube and NaOAc was measured. Then, CH_3CN (3.0 mL, used as received; the same as one used in catalysis) was added, and the tube was allowed to stir for 30 min at room temperature. To remove the solvent, the sample tube was placed in an oven under 100 °C until the NaOAc was completely dried. The weight of the tube and NaOAc was measured again and the lost weight was used to estimate the solubility (wt. %) of NaOAc in CH_3CN. The solubility of KOAc, LiOAc and CsOAc was estimated similarly.

Table S2 Estimated solubility of NaOAc, KOAc, LiOAc and CsOAc in CH_3CN used.

Base	NaOAc	KOAc	LiOAc	CsOAc
Solubility	7.3%	5.1%	0.8 %	0.4%

4.4 Transformations of 2-phenylpropanoic acid (1 mmol scale)

To an oven dried Schlenk tube, $CeCl_3$ (10 mol%), phenylacetic acid (1 mmol), NaOAc (1 mmol) and CH_3CN (2 mL) were added under air. The reaction tube was allowed to stir at room temperature under blue light irradiation (465 nm) for 15 h. After reaction, the product was purified by silica gel column chromatography (hexane: ethyl acetate = 10:1), affording (1-hydroperoxyethyl)benzene as a colourless oil (120.06 mg, 80%).

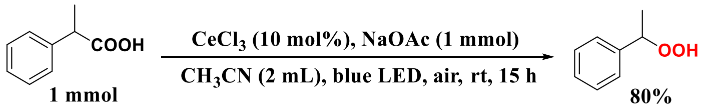

To an oven dried Schlenk tube, $CeCl_3$ (10 mol%), phenylacetic acid (1 mmol), 2,6-lutidine (1 mmol) and CH_3CN (2 mL) were added under air. The reaction tube was allowed to stir at room temperature under blue light irradiation (465 nm) for 15 h. After reaction, the product was purified by silica gel column chromatography (hexane: ethyl acetate = 10:1), affording acetophenone as a yellow oil (99.05 mg, 66%).

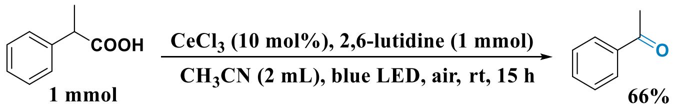

5 Mechanistic studies

5.1 HRMS detection of Ce(III) complex formed in the in situ reaction of $CeCl_3$ with phenylacetic acid

To an oven dried Schlenk tube, $CeCl_3$ (10 mol%), phenylacetic acid (0.5 mmol), NaOAc or 2,6-lutidine (0.5 mmol) and CH_3CN (2 mL) were added under air. The reaction tube was allowed to stir at room

temperature under blue light irradiation (465 nm) for 2 h. After reaction, 1 mL of the mixture was taken out as the sample. After the following evaporation, this sample was analysed by ESI-MS.

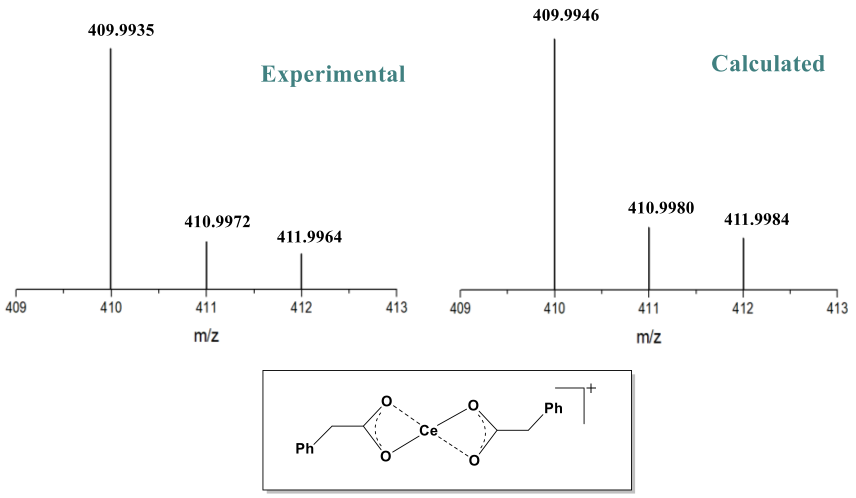

Figure S3 HRMS spectra of Ce(III) complex formed in the in situ reaction of $CeCl_3$ with phenylacetic acid.

5.2 Decarboxylative oxygenation of ethyl 2-phenylacetate

To an oven dried Schlenk tube, $CeCl_3$ (10 mol%), ethyl 2-phenylacetate (0.5 mmol), NaOAc or 2,6-lutidine (0.5 mmol) and CH_3CN (2 mL) were added under air. The reaction tube was allowed to stir at room temperature under blue light irradiation (465 nm) for 15 h. After the reaction, water (2 mL), chloroform-*d* (1 mL), and mesitylene (17.3 mg) were added. The product yields were obtained by ^1H NMR analysis of the organic layer.

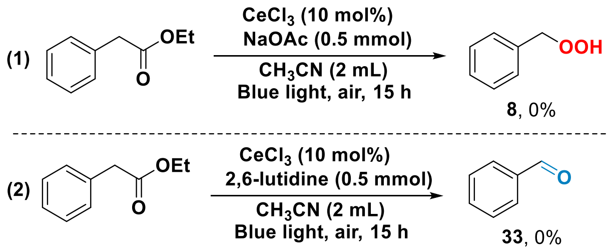

Scheme S2. Decarboxylative oxygenation of ethyl 2-phenylacetate.

5.3 Kinetic behaviours of decarboxylative oxygenation of α-methylphenylacetic acid and 1

Decarboxylative oxygenation of α-methylphenylacetic acid: To an oven dried Schlenk tube, $CeCl_3$ (10 mol%), α-methylphenylacetic acid (0.5 mmol), 2,6-lutidine (0.5 mmol), CH_3CN (2 mL) and 1-bromo-3,5-bis(trifluoromethyl)benzene (0.2 mmol) as internal standard were added under air. The reaction tube was allowed to stir at room temperature under blue light irradiation (465 nm). Every 30 min, 0.1 mL of the reaction mixture was taken out for ^1H NMR analysis.

Transformation of **1**: To an oven dried Schlenk tube, $CeCl_3$ (10 mol%), **1** (0.5 mmol), 2,6-lutidine (0.5 mmol), CH_3CN (2 mL) and 1-bromo-3,5-bis(trifluoromethyl)benzene (0.2 mmol) as internal standard were added under air. The reaction tube was allowed to stir at room temperature under blue light irradiation (465 nm). Every 30 min, 0.1 mL of the reaction mixture was taken out for ^1H NMR analysis.

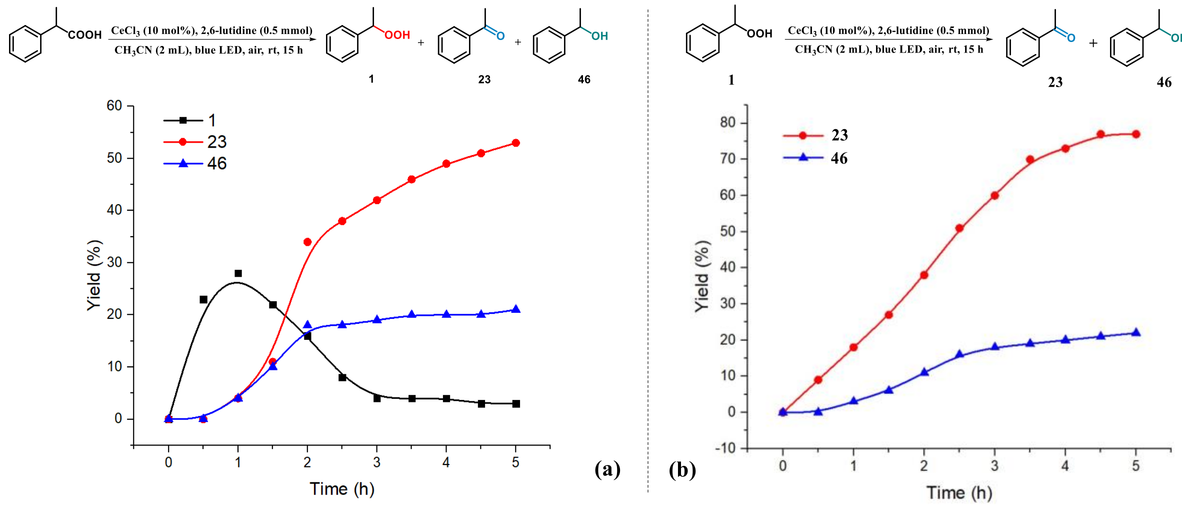

Figure S4 (a) Kinetic behaviour of α-methylphenylacetic acid in decarboxylative oxygenation; (b) Kinetic behaviour of **1** in decarboxylative oxygenation.

5.4 Transformation of 1 in different conditions

Transformation of 1 in the presence of NaOAc or 2,6-lutidine

1 + NaOAc: To an oven dried Schlenk tube, $CeCl_3$ (10 mol%), **1** (0.5 mmol), NaOAc (0.5 mmol) and CH_3CN (2 mL) were added under air. The reaction tube was allowed to stir at room temperature under blue light irradiation (465 nm) for 15 h. After the reaction, water (2 mL), chloroform-*d* (1 mL), and mesitylene (17.3 mg) were added. The product yields were obtained by ^{1}H NMR analysis of the organic layer.

1 + 2,6-lutidine: To an oven dried Schlenk tube, $CeCl_3$ (10 mol%), **1** (0.5 mmol), 2,6-lutidine (0.5 mmol) and CH_3CN (2 mL) were added under air. The reaction tube was allowed to stir at room temperature under blue light irradiation (465 nm) for 15 h. After the reaction, water (2 mL), chloroform-*d* (1 mL), and mesitylene (17.3 mg) were added. The product yields were obtained by ^{1}H NMR analysis of the organic layer.

Transformation of 1 in the presence of 2,6-lutidine under N_2 without catalyst

1 + 2,6-lutidine: To an oven dried Schlenk tube, **1** (0.5 mmol), 2,6-lutidine (0.5 mmol) and CH_3CN (2 mL) were added under N_2. The reaction tube was allowed to stir at room temperature under blue light irradiation (465 nm) for 15 h. After the reaction, water (2 mL), chloroform-*d* (1 mL), and mesitylene (17.3 mg) were added. The product yields were obtained by ^{1}H NMR analysis of the organic layer.

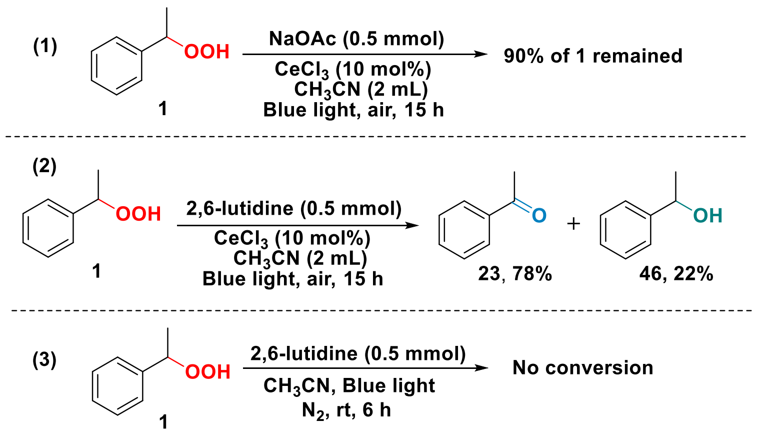

Scheme S3. Transformation of **1** in the presence of NaOAc or 2,6-lutidine.

5.5 UV-Vis spectra of $CeCl_3$ in the presence of NaOAc and Et_3N

$CeCl_3$: To an oven dried Schlenk tube, $CeCl_3$ (0.05 mmol) and CH_3CN/MeOH (2 mL, 1:1) were added. The reaction tube was allowed to stir 5 min at room temperature. 0.01 mL of the solution was then taken out and diluted to 5 mL for UV-Vis analysis.

$CeCl_3$ + α-methylphenylacetic acid: To an oven dried Schlenk tube, $CeCl_3$ (0.05 mmol), α-methylphenylacetic acid (0.1 mmol) and CH_3CN/MeOH (2 mL, 1:1) were added. The reaction tube was allowed to stir 5 min at room temperature. 0.01 mL of the solution was then taken out and diluted to 5 mL for UV-Vis analysis.

$CeCl_3$ + NaOAc: To an oven dried Schlenk tube, $CeCl_3$ (0.05 mmol), NaOAc (0.1 mmol) and CH_3CN/MeOH (2 mL, 1:1) were added. The reaction tube was allowed to stir 5 min at room temperature. 0.01 mL of the solution was then taken out and diluted to 5 mL for UV-Vis analysis.

$CeCl_3$ + Et_3N: To an oven dried Schlenk tube, $CeCl_3$ (0.05 mmol), Et_3N (0.1 mmol) and CH_3CN/MeOH (2 mL, 1:1) were added. The reaction tube was allowed to stir 5 min at room temperature. 0.01 mL of the solution was then taken out and diluted to 5 mL for UV-Vis analysis.

$CeCl_3$ + 2,6-lutidine: To an oven dried Schlenk tube, $CeCl_3$ (0.05 mmol), 2,6-lutidine (0.1 mmol) and CH_3CN/MeOH (2 mL, 1:1) were added. The reaction tube was allowed to stir 5 min at room temperature. 0.01 mL of the solution was then taken out and diluted to 10 mL for UV-Vis analysis.

We started from the detection of the UV-Vis spectrum of the mixture of $CeCl_3$ and 2,6-lutidine. However, the UV-Vis spectrum of the mixture of $CeCl_3$ and 2,6-lutidine is overwhelmed by the absorption of 2,6-lutidine (Figure S5(a)). Hence, 2,6-lutidine was replaced by NEt_3 for a better analysis. The UV-Vis spectra of $CeCl_3$ in the presence of NaOAc or NEt_3 with or without phenylacetic acid show a peak at around 315 nm, assignable to $CeCl_3$ (Figure, S5(b)).[4] However, the spectrum of $CeCl_3$ and NaOAc also showed two stronger absorption bands at around 259 and 276 nm, which may indicate the coordination of NaOAc to $CeCl_3$.[5] This coordination could inhibit further transformation of hydroperoxides by Ce species.

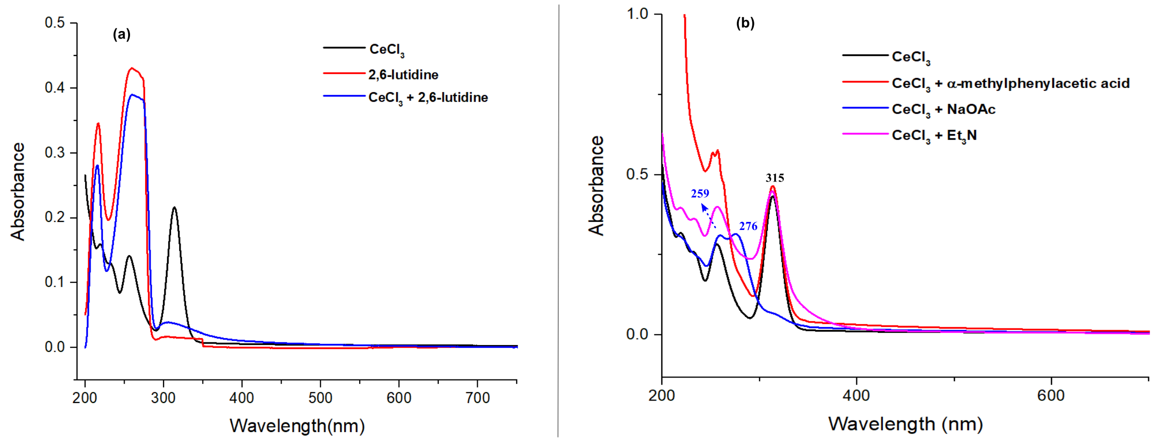

Figure S5 (a) UV-Vis spectra of $CeCl_3$, 2,6-lutidine and the mixture of $CeCl_3$ and 2,6-lutidine; (b) UV-Vis spectra of $CeCl_3$, the mixture of $CeCl_3$ and α-methylphenylacetic acid, the mixture of $CeCl_3$ and NaOAc, and the mixture of $CeCl_3$ and Et_3N.

6 Analytical data of products

6.1 Hydroperoxide products

Hydroperoxides (**1-22**) were synthesized according to the following steps. To an oven dried Schlenk tube, a Ce catalyst (10 mol%), NaOAc (0.5 mmol), carboxylic acids (0.5 mmol) and 2 mL of CH_3CN were added under air. The Schlenk tube was allowed to stir under blue light (465 nm) for 15 h. After the reaction, the hydroperoxides were separated by silica gel column chromatography. For peroxides **14**, **15** and **16**, Na_2CO_3 (0.5 mmol) was used as base, and the reaction was conducted under N_2/O_2 (1:2) atmosphere.

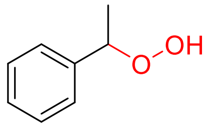

(1-Hydroperoxyethyl)benzene (1)[6]
Colourless oil (94%, 70.5 mg), purified by silica gel column chromatography (Hexane: Ethyl Acetate = 10: 1).
^{1}H NMR (400 MHz, $CDCl_3$) δ 7.85 (s, 1H), 7.42 – 7.28 (m, 5H), 5.08 (q, J = 6.6 Hz, 1H), 1.48 (d, J = 6.6 Hz, 3H).
^{13}C NMR (101 MHz, $CDCl_3$) δ 141.36, 128.63, 128.23, 126.50, 83.74, 20.04.

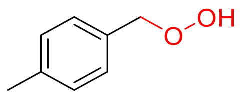

1-(Hydroperoxymethyl)-4-methylbenzene (2)[7]
Colourless oil (80%, 60.0 mg), purified by silica gel column chromatography (Hexane: Ethyl Acetate = 10: 1).
^{1}H NMR (400 MHz, $CDCl_3$) δ 7.90 (s, 1H), 7.29 (d, J = 7.8 Hz, 2H), 7.20 (d, J = 7.8 Hz, 2H), 4.97 (s, 2H), 2.37 (s, 3H).
^{13}C NMR (101 MHz, $CDCl_3$) δ 138.52, 132.61, 129.29, 129.10, 79.09, 21.22.

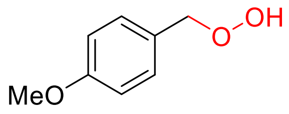

1-(Hydroperoxymethyl)-4-methoxybenzene (3)[7]
Colourless oil (56%, 46.5 mg), purified by silica gel column chromatography (Hexane: Ethyl Acetate = 10: 1).
^{1}H NMR (400 MHz, $CDCl_3$) δ 8.06 (s, 1H), 7.32 (d, J = 8.6 Hz, 2H), 6.91 (d, J = 8.6 Hz, 2H), 4.94 (s, 2H), 3.81 (s, 3H).
^{13}C NMR (101 MHz, $CDCl_3$) δ 159.88, 130.72, 127.68, 113.94, 78.83, 55.27.

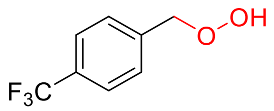

1-(hydroperoxymethyl)-4-(trifluoromethyl)benzene (4)
Colourless oil (67%, 68.4 mg), purified by silica gel column chromatography (Hexane: Ethyl Acetate = 10: 1).
^{1}H NMR (400 MHz, $CDCl_3$) δ 8.09 (d, J = 1.5 Hz, 1H), 7.65 (d, J = 7.9 Hz, 2H), 7.52 (d, J = 7.9 Hz, 2H), 5.06 (s, 2H).
^{13}C NMR (101 MHz, $CDCl_3$) δ 139.99, 130.67 (q, J_{C-F} = 32.5 Hz), 128.81, 125.54 (q, J_{C-F} = 3.8 Hz), 124.01 (q, J_{C-F} = 273.1 Hz), 78.21.
HRMS (ESI) calcd for $C_8H_7F_3O_2$ $[M+Na]^+$: 215.0290; found: 215.0283.

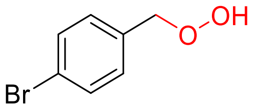

1-Bromo-4-(hydroperoxymethyl)benzene (5)[7]
Colourless oil (61%, 65.3 mg), purified by silica gel column chromatography (Hexane: Ethyl Acetate = 10: 1).
^{1}H NMR (400 MHz, $CDCl_3$) δ 8.09 (s, 1H), 7.42 (d, J = 8.3 Hz, 2H), 7.17 (d, J = 8.3 Hz, 2H), 4.85 (s, 2H).
^{13}C NMR (101 MHz, $CDCl_3$) δ 134.77, 131.70, 130.53, 122.63, 78.31.

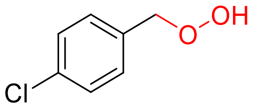

1-Chloro-4-(hydroperoxymethyl)benzene (6)[7]
Colourless oil (82%, 69.7 mg), purified by silica gel column chromatography (Hexane: Ethyl Acetate = 10: 1).
^{1}H NMR (400 MHz, $CDCl_3$) δ 8.09 (s, 1H), 7.38 – 7.30 (m, 4H), 4.96 (s, 2H).
^{13}C NMR (101 MHz, $CDCl_3$) δ 134.48, 134.27, 130.26, 128.76, 78.30.

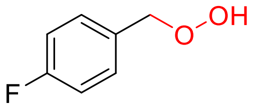

1-Fluoro-4-(hydroperoxymethyl)benzene (7)[7]
Colourless oil (63%, 48.5 mg), purified by silica gel column chromatography (Hexane: Ethyl Acetate = 10: 1).
^{1}H NMR (400 MHz, $CDCl_3$) δ 8.06 (s, 1H), 7.41 – 7.33 (m, 2H), 7.09 – 7.05 (m, 2H), 4.96 (s, 2H).
^{13}C NMR (101 MHz, $CDCl_3$) δ 162.90 (d, J_{C-F} = 248.1 Hz), 131.52 (d, J_{C-F} = 3.2 Hz), 130.89 (d, J_{C-F} = 8.4 Hz), 115.50 (d, J = 21.4 Hz), 78.38.

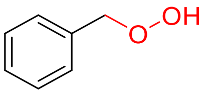

(Hydroperoxymethyl)benzene (8)[7]
Colourless oil (57%, 38.8 mg), purified by silica gel column chromatography (Hexane: Ethyl Acetate = 10: 1).
^{1}H NMR (400 MHz, $CDCl_3$) δ 8.02 (s, 1H), 7.43 – 7.33 (m, 5H), 5.01 (s, 2H).
^{13}C NMR (101 MHz, $CDCl_3$) δ 135.73, 128.97, 128.60, 79.21.

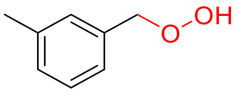

1-(Hydroperoxymethyl)-3-methylbenzene (9)
Colourless oil (61%, 45.8 mg), purified by silica gel column chromatography (Hexane: Ethyl Acetate = 10: 1).
^{1}H NMR (400 MHz, $CDCl_3$) δ 8.04 (s, 1H), 7.32 – 7.26 (m, 1H), 7.22 – 7.17 (m, 3H), 4.98 (s, 2H), 2.38 (s, 3H).
^{13}C NMR (101 MHz, $CDCl_3$) δ 138.31, 135.59, 129.67, 129.34, 128.49, 126.00, 79.24, 21.31.
HRMS (ESI) calcd for $C_8H_{10}O_2$ $[M+Na]^+$: 161.0573; found: 161.0571.

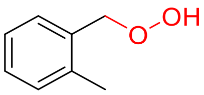

1-(Hydroperoxymethyl)-2-methylbenzene (10)[7]

Colourless oil (59%, 44.3 mg), purified by silica gel column chromatography (Hexane: Ethyl Acetate = 10: 1).
^{1}H NMR (400 MHz, $CDCl_3$) δ 8.02 (s, 1H), 7.36 – 7.31 (m, 1H), 7.30 – 7.25 (m, 1H), 7.24 – 7.18 (m, 2H), 5.06 (s, 2H), 2.40 (s, 3H).
^{13}C NMR (101 MHz, $CDCl_3$) δ 137.82, 133.31, 130.50, 130.42, 128.90, 125.92, 77.34, 18.87.

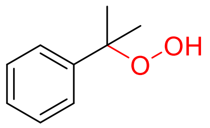

(2-Hydroperoxypropan-2-yl)benzene (11)[6]
Colourless oil (94%, 77.1 mg), purified by silica gel column chromatography (Hexane: Ethyl Acetate = 10: 1).
^{1}H NMR (400 MHz, $CDCl_3$) δ 7.51 – 7.46 (m, 2H), 7.41 – 7.38 (m, 3H), 7.33 – 7.28 (m, 1H), 1.62 (s, 6H).
^{13}C NMR (101 MHz, $CDCl_3$) δ 144.56, 128.51, 127.43, 125.36, 83.93, 26.04.

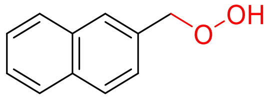

2-(Hydroperoxymethyl)naphthalene (12)[7]
White powder (43%, 40.0 mg), purified by silica gel column chromatography (Hexane: Ethyl Acetate = 10: 1).
^{1}H NMR (400 MHz, $CDCl_3$) δ 8.07 (s, 1H), 7.91 – 7.82 (m, 4H), 7.56 – 7.48 (m, 3H), 5.17 (s, 2H).
^{13}C NMR (101 MHz, $CDCl_3$) δ 133.33, 133.16, 128.43, 128.30, 127.99, 127.71, 126.35, 126.31, 79.32.

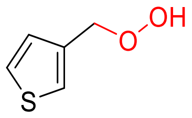

3-(Hydroperoxymethyl)thiophene (13)
Yellow oil (56%, 39.8 mg), purified by silica gel column chromatography (Hexane: Ethyl Acetate = 10: 1).
^{1}H NMR (400 MHz, $CDCl_3$) δ 8.06 (s, 1H), 7.37 – 7.32 (m, 2H), 7.16 – 7.11 (m, 1H), 5.02 (s, 2H).
^{13}C NMR (101 MHz, $CDCl_3$) δ 136.73, 127.94, 126.29, 125.33, 73.77.
HRMS (ESI) calcd for $C_5H_6O_2S$ $[M+Na]^+$: 152.9981; found: 152.9987.

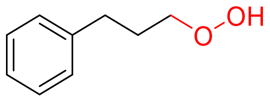

(3-Hydroperoxypropyl)benzene (14)[8]
Colourless oil (66%, 54.1 mg), purified by silica gel column chromatography (Hexane: Ethyl Acetate = 10: 1).
^{1}H NMR (400 MHz, $CDCl_3$) δ 7.82 (s, 1H), 7.32 – 7.26 (m, 2H), 7.21-7.18 (m, 3H), 4.05 (t, J = 6.4 Hz, 2H), 2.71(t, J = 7.8 Hz, 2H), 2.04 – 1.94 (m, 2H).
^{13}C NMR (101 MHz, $CDCl_3$) δ 141.47, 128.42, 125.96, 76.22, 32.01, 29.14.

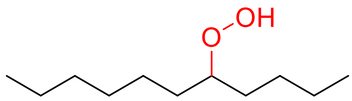

5-Hydroperoxyundecane (15)
Colourless oil (67%, 67.1 mg), purified by silica gel column chromatography (Hexane: Ethyl Acetate = 20: 1).
^{1}H NMR (400 MHz, $CDCl_3$) δ 7.57 (s, 1H), 3.92 – 3.86 (m, 1H), 1.69 – 1.55 (m, 2H), 1.55 – 1.43 (m, 2H), 1.40-1.25 (m, 12H), 0.95-0.86 (m, 6H).

^{13}C NMR (101 MHz, $CDCl_3$) δ 85.74, 31.99, 31.78, 31.68, 29.42, 27.54, 25.33, 22.81, 22.60, 14.05, 14.00.
HRMS (ESI) calcd for $C_{11}H_{24}O_2$ $[M+Na]^+$: 211.1669; found: 211.1671.

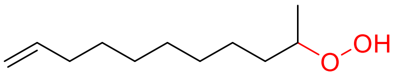

10-Hydroperoxyundec-1-ene (16)
Colourless oil (39%, 38.6 mg), purified by silica gel column chromatography (Hexane: Ethyl Acetate = 20: 1).
^{1}H NMR (400 MHz, $CDCl_3$) δ 7.64 (s, 1H), 5.86-5.76 (m, 1H), 5.02 – 4.88 (m, 2H), 4.10 – 4.02 (m, 1H), 2.08 – 1.98 (m, 2H), 1.69 – 1.55 (m, 2H), 1.40 – 1.27 (m, 10H), 1.22 (d, J = 6.1 Hz, 3H).
^{13}C NMR (101 MHz, $CDCl_3$) δ 139.18, 114.13, 81.71, 33.97, 33.76, 29.60, 29.36, 29.02, 28.88, 25.37, 18.14.
HRMS (ESI) calcd for $C_{11}H_{22}O_2$ $[M+Na]^+$: 209.1512; found: 209.1511.

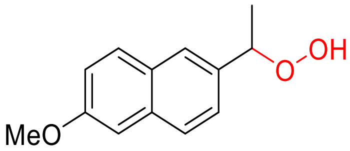

2-(1-Hydroperoxyethyl)-6-methoxynaphthalene (17)
White powder (88%, 101.24 mg), purified by silica gel column chromatography (Hexane: Ethyl Acetate = 10: 1)
^{1}H NMR (400 MHz, $CDCl_3$) δ 7.91 (s, 1H), 7.78 – 7.71 (m, 3H), 7.47 (dd, J = 8.4, 1.6 Hz, 1H), 7.20 – 7.10 (m, 2H), 5.21 (q, J = 6.6 Hz, 1H), 3.92 (s, 3H), 1.56 (d, J = 6.6 Hz, 3H).
^{13}C NMR (101 MHz, $CDCl_3$) δ 157.83, 136.32, 134.48, 129.43, 128.65, 127.36, 125.79, 124.66, 119.07, 105.67, 83.85, 55.28, 19.95.
HRMS (ESI) calcd for $C_{13}H_{14}O_3$ $[M+Na]^+$: 241.0835; found: 241.0842.

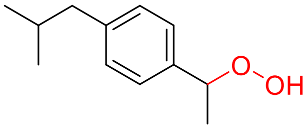

1-(1-Hydroperoxyethyl)-4-isobutylbenzene (18)
Colourless oil (90%, 92.8 mg), purified by silica gel column chromatography (Hexane: Ethyl Acetate = 10: 1)
^{1}H NMR (400 MHz, $CDCl_3$) δ 7.89 (s, 1H), 7.29 (d, J = 8.0 Hz, 2H), 7.17 (d, J = 8.0 Hz, 2H), 5.06 (q, J = 6.6 Hz, 1H), 2.49 (d, J = 7.2 Hz, 2H), 1.95 – 1.81 (m, 1H), 1.48 (d, J = 6.6 Hz, 3H), 0.93 (d, J = 6.6 Hz, 6H).
^{13}C NMR (101 MHz, $CDCl_3$) δ 141.83, 138.40, 129.32, 126.39, 83.59, 45.09, 30.15, 22.35, 22.34, 19.88.
HRMS (ESI) calcd for $C_{12}H_{18}O_2$ $[M+Na]^+$: 217.1199; found: 217.1203.

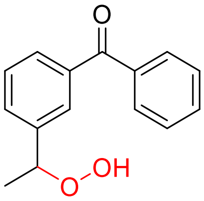

(3-(1-Hydroperoxyethyl)phenyl)(phenyl)methanone (19)
White powder (99%, 125.8 mg), purified by silica gel column chromatography (Hexane: Ethyl Acetate = 10: 1)
^{1}H NMR (400 MHz, $CDCl_3$) δ 8.65 (s, 1H), 7.83 – 7.75 (m, 3H), 7.69 (d, J = 7.6 Hz, 1H), 7.61 – 7.57 (m, 2H), 7.49-7.44 (m, 3H), 5.13 (q, J = 6.6 Hz, 1H), 1.48 (d, J = 6.6 Hz, 3H).
^{13}C NMR (101 MHz, $CDCl_3$) δ 196.87, 142.28, 137.73, 137.27, 132.59, 130.54, 130.08, 129.85, 128.43, 128.29, 127.93, 83.09, 20.18.
HRMS (ESI) calcd for $C_{15}H_{14}O_3$ $[M+Na]^+$: 265.0835; found: 265.0829.

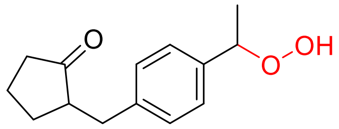

2-(4-(1-Hydroperoxyethyl)benzyl)cyclopentan-1-one (20)
Colourless oil (86%, 105.8 mg), purified by silica gel column chromatography (Hexane: Ethyl Acetate = 10: 1)
^{1}H NMR (400 MHz, $CDCl_3$) δ 8.29 (s, 1H), 7.30 (d, J = 8.0 Hz, 2H), 7.18 (d, J = 8.0 Hz, 2H), 5.06 (q, J = 6.6 Hz, 1H), 3.14 (dd, J = 13.2, 5.0 Hz, 1H), 2.60 – 2.50 (m, 1H), 2.39 – 2.31 (m, 2H), 2.17 – 2.04 (m, 2H), 2.01 – 1.93 (m, 1H), 1.81 – 1.67 (m, 1H), 1.62 – 1.51 (m, 1H), 1.48 (d, J = 6.6 Hz, 3H).
^{13}C NMR (101 MHz, $CDCl_3$) δ 139.93, 139.21, 129.03, 126.65, 83.33, 50.90, 50.88, 38.09, 35.19, 29.12, 20.44, 19.93.
HRMS (ESI) calcd for $C_{14}H_{18}O_3$ $[M+Na]^+$: 257.1148; found: 257.1148.

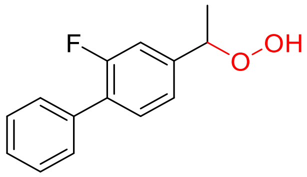

2-Fluoro-4-(1-hydroperoxyethyl)-1,1'-biphenyl (21)
White powder (79%, 96.4 mg), purified by silica gel column chromatography (Hexane: Ethyl Acetate = 10: 1)
^{1}H NMR (400 MHz, $CDCl_3$) δ 8.00 (s, 1H), 7.58 – 7.55 (m, 2H), 7.50 – 7.44 (m, 3H), 7.42 – 7.36 (m, 1H), 7.25 – 7.17 (m, 2H), 5.12 (q, J = 6.6 Hz, 1H), 1.51 (d, J = 6.6 Hz, 3H).
^{13}C NMR (101 MHz, $CDCl_3$) δ 159.81 (d, J_{C-F} = 249.7 Hz), 143.13 (d, J_{C-F} = 7.1 Hz), 135.41 (d, J_{C-F} = 1.4 Hz), 130.95 (d, J_{C-F} = 3.8 Hz), 128.94 (d, J_{C-F} = 2.8 Hz), 128.79 (d, J_{C-F} = 13.7 Hz), 128.44, 127.74, 122.36 (d, J_{C-F} = 3.4 Hz), 114.08 (d, J_{C-F} = 23.5 Hz), 82.89 (d, J_{C-F} = 1.2 Hz), 20.00.
HRMS (ESI) calcd for $C_{14}H_{13}FO_2$ $[M+Na]^+$: 255.0792; found: 255.0789.

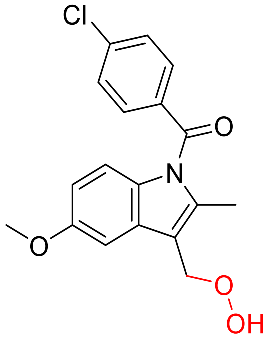

(4-Chlorophenyl)(3-(hydroperoxymethyl)-5-methoxy-2-methyl-1*H*-indol-1-yl)methanone (22)
White powder (47%, 83.9 mg), purified by silica gel column chromatography (Hexane: Ethyl Acetate = 4: 1)
^{1}H NMR (400 MHz, $CDCl_3$) δ 8.05 (s, 1H), 7.67 (d, J = 6.6 Hz, 2H), 7.48 (d, J = 6.6 Hz, 2H), 7.07 (s, 1H), 6.84 (d, J = 9.1 Hz, 1H), 6.70 – 6.67 (m,1H), 5.18 (s, 2H), 3.84 (s, 3H), 2.45 (s, 3H).
^{13}C NMR (101 MHz, $CDCl_3$) δ 168.43, 156.19, 139.62, 138.78, 133.51, 131.26, 130.83, 130.32, 129.20, 114.92, 113.64, 112.12, 101.14, 69.25, 55.73, 13.16.
HRMS (ESI) calcd for $C_{18}H_{16}ClNO_4$ $[M+Na]^+$: 368.0660; found: 368.0662.

6.2 Aldehyde and ketone products

Aldehydes and ketones (**23-45**) were synthesized according to the following steps. To an oven dried Schlenk tube, a Ce catalyst (10 mol%), 2,6-lutidine (0.5 mmol), carboxylic acids (0.5 mmol) and 2 mL of CH_3CN were added under air. The Schlenk tube was allowed to stir under blue light (465 nm) for 15 h. After the reaction, the hydroperoxides were separated by silica gel column chromatography. For

products **28**, **39**, **44** and **45**, pyridine (0.5 mmol) was added as base. For product **37**, UV (365 nm) was used as light source.

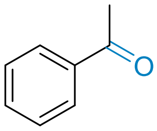

Acetophenone (23)[9]
Yellow oil (74%, 55.5 mg), purified by silica gel column chromatography (Hexane: Ethyl Acetate = 10: 1).
^{1}H NMR (400 MHz, $CDCl_3$) δ 7.96 (d, *J* = 7.2 Hz, 2H), 7.57 (t, *J* = 7.4 Hz, 1H), 7.47 (t, *J* = 7.8 Hz, 2H), 2.61 (s, 3H).
^{13}C NMR (101 MHz, $CDCl_3$) δ 198.15, 137.13, 133.09, 128.56, 128.30, 26.60.

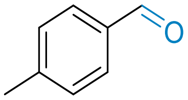

4-Methylbenzaldehyde (24)[9]
Colourless oil (68%, 51.0 mg), purified by silica gel column chromatography (Hexane: Ethyl Acetate = 10: 1).
^{1}H NMR (400 MHz, $CDCl_3$) δ 9.97 (s, 1H), 7.78 (d, *J* = 8.0 Hz, 2H), 7.33 (d, *J* = 8.0 Hz, 2H), 2.44 (s, 3H).
^{13}C NMR (101 MHz, $CDCl_3$) δ 192.01, 145.54, 134.19, 129.85, 129.70, 21.86.

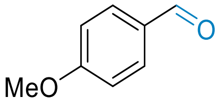

4-Methoxybenzaldehyde (25)[10]
Colourless oil (63%, 52.3 mg), purified by silica gel column chromatography (Hexane: Ethyl Acetate = 10: 1).
^{1}H NMR (400 MHz, $CDCl_3$) δ 9.88 (s, 1H), 7.83 (d, *J* = 8.7 Hz, 2H), 7.00 (d, *J* = 8.7 Hz, 2H), 3.88 (s, 3H).
^{13}C NMR (101 MHz, $CDCl_3$) δ 190.80, 164.56, 131.95, 129.90, 114.26, 55.54.

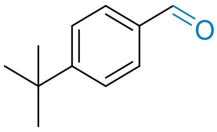

4-(*Tert-butyl*)benzaldehyde (26)[9]
Colourless oil (70%, 67.2 mg), purified by silica gel column chromatography (Hexane: Ethyl Acetate = 10: 1).
^{1}H NMR (400 MHz, $CDCl_3$) δ 9.98 (s, 1H), 7.82 (d, *J* = 8.3 Hz, 2H), 7.55 (d, *J* = 8.3 Hz, 2H), 1.35 (s, 9H).
^{13}C NMR (101 MHz, $CDCl_3$) δ 192.04, 158.42, 134.04, 129.67, 125.96, 35.32, 31.03.

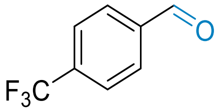

4-(Trifluoromethyl)benzaldehyde (27)[11]
Colourless oil (49%, 50.0 mg), purified by silica gel column chromatography (Hexane: Ethyl Acetate = 10: 1).
^{1}H NMR (400 MHz, $CDCl_3$) δ 9.56 (s, 1H), 7.47 (d, *J* = 8.0 Hz, 2H), 7.27 (d, *J* = 8.0 Hz, 2H).
^{13}C NMR (101 MHz, $CDCl_3$) δ 191.06, 138.65, 135.60 (q, J_{C-F} = 32.8 Hz), 129.90, 126.10 (q, J_{C-F} = 3.8 Hz), 123.43 (q, J_{C-F} = 273.9 Hz).

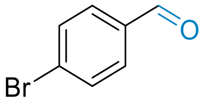

4-Bromobenzaldehyde (28)[9]

White powder (74%, 79.2 mg), purified by silica gel column chromatography (Hexane: Ethyl Acetate = 10: 1).

^{1}H NMR (400 MHz, $CDCl_3$) δ 9.97 (s, 1H), 7.74 (d, *J* = 8.4 Hz, 2H), 7.68 (d, *J* = 8.4 Hz, 2H).

^{13}C NMR (101 MHz, $CDCl_3$) δ 191.01, 135.05, 132.41, 130.94, 129.74.

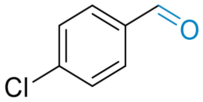

4-Chlorobenzaldehyde (29)[9]

White powder (71%, 60.4 mg), purified by silica gel column chromatography (Hexane: Ethyl Acetate =10: 1).

^{1}H NMR (400 MHz, $CDCl_3$) δ 9.99 (s, 1H), 7.83 (d, *J* = 8.4 Hz, 2H), 7.52 (d, *J* = 8.4 Hz, 2H).

^{13}C NMR (101 MHz, $CDCl_3$) δ 190.73, 140.80, 134.61, 130.79, 129.33.

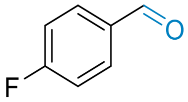

4-Fluorobenzaldehyde (30)[12]

Colourless oil (58%, 44.7 mg), purified by silica gel column chromatography (Hexane: Ethyl Acetate = 10: 1).

^{1}H NMR (400 MHz, $CDCl_3$) δ 9.96 (s, 1H), 7.93 – 7.88 (m, 2H), 7.24 – 7.17 (m, 2H).

^{13}C NMR (101 MHz, $CDCl_3$) δ 190.47, 166.51 (d, J_{C-F} = 257.8 Hz), 132.95 (d, J_{C-F} = 2.8 Hz), 132.21 (d, J_{C-F} = 9.8 Hz), 116.33 (d, J_{C-F} = 22.3 Hz).

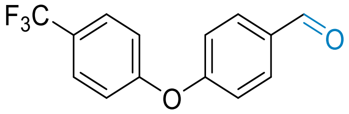

4-(4-(Trifluoromethyl)phenoxy)benzaldehyde (31)[13]

White powder (66%, 97.7 mg), purified by silica gel column chromatography (Hexane: Ethyl Acetate = 10: 1).

^{1}H NMR (400 MHz, $CDCl_3$) δ 9.96 (s, 1H), 7.97 – 7.83 (m, 2H), 7.66 (d, *J* = 8.4 Hz, 2H), 7.21 – 7.07 (m, 4H).

^{13}C NMR (101 MHz, $CDCl_3$) δ 190.64, 161.64, 158.34, 132.21, 132.03, 127.49 (q, J_{C-F} = 3.7 Hz), 126.64 (q, J_{C-F} = 33.0 Hz), 123.92 (q, J_{C-F} = 272.7 Hz), 119.73, 118.65.

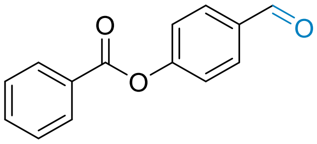

4-Formylphenyl benzoate (32)[14]

White powder (69%, 88.3 mg), purified by silica gel column chromatography (Hexane: Ethyl Acetate = 10: 1).

^{1}H NMR (400 MHz, $CDCl_3$) δ 10.03 (s, 1H), 8.21 (d, *J* = 7.2 Hz, 2H), 7.98 (d, *J* = 8.6 Hz, 2H), 7.69-7.61 (m, 1H), 7.54 (t, *J* = 7.8 Hz, 2H), 7.42 (d, *J* = 8.5 Hz, 2H).

^{13}C NMR (101 MHz, $CDCl_3$) δ 190.92, 164.47, 155.67, 134.04, 133.99, 131.24, 130.25, 128.88, 128.69, 122.51.

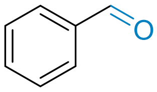

Benzaldehyde (33)[10]

Colourless oil (75%, 51.0 mg), purified by silica gel column chromatography (Hexane: Ethyl Acetate = 10: 1).

^{1}H NMR (400 MHz, $CDCl_3$) δ 10.02 (s, *J* = 1.8 Hz, 1H), 7.92 – 7.83 (m, 2H), 7.65-7.61 (m, 1H), 7.52 (t, *J* = 8.0 Hz, 2H).
^{13}C NMR (101 MHz, $CDCl_3$) δ 192.38, 136.36, 134.43, 129.70, 128.95.

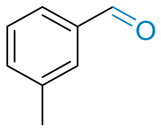

3-Methylbenzaldehyde (34)[15]
Colourless oil (60%, 45.0 mg), purified by silica gel column chromatography (Hexane: Ethyl Acetate = 10: 1).
^{1}H NMR (400 MHz, $CDCl_3$) δ 9.99 (s, 1H), 7.72 – 7.65 (m, 2H), 7.47 – 7.39 (m, 2H), 2.44 (s, 3H).
^{13}C NMR (101 MHz, $CDCl_3$) δ 192.60, 138.90, 136.47, 135.27, 130.00, 128.86, 127.21, 21.17.

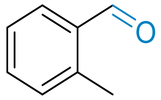

2-Methylbenzaldehyde (35)[16]
Colourless oil (43%, 32.3 mg), purified by silica gel column chromatography (Hexane: Ethyl Acetate = 10: 1).
^{1}H NMR (400 MHz, $CDCl_3$) δ 10.28 (s, 1H), 7.80 (dd, *J* = 7.7, 1.5 Hz, 1H), 7.48 (td, *J* = 7.5, 1.5 Hz, 1H), 7.36 (t, *J* = 7.5 Hz, 1H), 7.27 (d, *J* = 7.5 Hz, 1H), 2.68 (s, 3H).
^{13}C NMR (101 MHz, $CDCl_3$) δ 192.83, 140.62, 134.14, 133.64, 132.04, 131.76, 126.31, 19.57.

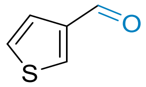

Thiophene-3-carbaldehyde (36)[17]
Yellow oil (70%, 49.7 mg), purified by silica gel column chromatography (Hexane: Ethyl Acetate =10: 1).
^{1}H NMR (400 MHz, $CDCl_3$) δ 9.94 (s, 1H), 8.13 – 8.12 (m, 1H), 7.55 – 7.54 (m, 1H), 7.38 (m, 1H).
^{13}C NMR (101 MHz, $CDCl_3$) δ 184.95, 143.03, 136.68, 127.38, 125.37.

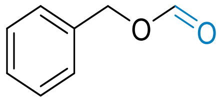

Benzyl formate (37)[18]
Colourless oil (75%, 62.3 mg), purified by silica gel column chromatography (Hexane: Ethyl Acetate = 10: 1).
^{1}H NMR (400 MHz, $CDCl_3$) δ 8.15 (s, 1H), 7.42 – 7.30 (m, 5H), 5.21 (s, 2H).
^{13}C NMR (101 MHz, $CDCl_3$) δ 160.76, 135.16, 128.64, 128.49, 128.34, 65.68.

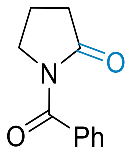

1-Benzoylpyrrolidin-2-one (38)[19]
White powder (66%, 72.3 mg), purified by silica gel column chromatography (Hexane: Ethyl Acetate = 5: 1).
^{1}H NMR (400 MHz, $CDCl_3$) δ 7.60 (d, *J* = 7.7 Hz, 2H), 7.51 (t, *J* = 7.4 Hz, 1H), 7.41 (t, *J* = 7.4 Hz, 2H), 3.96 (t, *J* = 7.0 Hz, 2H), 2.61 (t, *J* = 8.0 Hz, 2H), 2.19 – 2.11 (m, 2H).
^{13}C NMR (101 MHz, $CDCl_3$) δ 174.50, 170.69, 134.31, 131.89, 128.89, 127.76, 46.51, 33.29, 17.65.

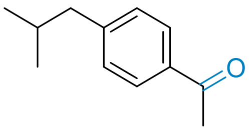

1-(4-Isobutylphenyl)ethan-1-one (39)[20]

Colourless oil (58%, 59.8 mg), purified by silica gel column chromatography (Hexane: Ethyl Acetate = 10: 1).

^{1}H NMR (400 MHz, $CDCl_3$) δ 7.88 (d, J = 8.3 Hz, 2H), 7.23 (d, J = 8.3 Hz, 2H), 2.59 (s, 3H), 2.53 (d, J = 7.2 Hz, 2H), 1.96 – 1.82 (m, 1H), 0.91 (d, J = 6.6 Hz, 6H).

^{13}C NMR (101 MHz, $CDCl_3$) δ 197.91, 147.59, 134.96, 129.27, 128.29, 45.37, 30.10, 26.53, 22.31.

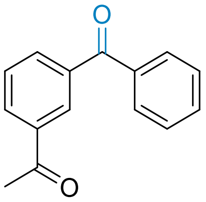

1-(3-Benzoylphenyl)ethan-1-one (40)[20]

White powder (83%, 105.4 mg), purified by silica gel column chromatography (Hexane: Ethyl Acetate = 5: 1).

^{1}H NMR (400 MHz, $CDCl_3$) δ 8.36 (s, 1H), 8.18 (d, J = 7.8 Hz, 1H), 7.99 (d, J = 7.8 Hz, 1H), 7.80 (d, J = 7.6 Hz, 2H), 7.61 (q, J = 7.6 Hz, 2H), 7.51 (t, J = 7.6 Hz, 2H), 2.65 (s, 3H).

^{13}C NMR (101 MHz, $CDCl_3$) δ 197.27, 195.83, 138.05, 137.16, 136.98, 134.22, 132.86, 131.73, 130.01, 129.67, 128.72, 128.48, 26.72.

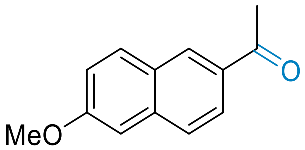

1-(6-Methoxynaphthalen-2-yl)ethan-1-one (41)[20]

White powder (82%, 94.3 mg), purified by silica gel column chromatography (Hexane: Ethyl Acetate = 5: 1).

^{1}H NMR (400 MHz, $CDCl_3$) δ 8.38 (s, 1H), 8.00 (dd, J = 8.6, 1.6 Hz, 1H), 7.84 (d, J = 8.9 Hz, 1H), 7.75 (d, J = 8.6 Hz, 1H), 7.21-7.14 (m, 2H), 3.94 (s, 3H), 2.69 (s, 3H).

^{13}C NMR (101 MHz, $CDCl_3$) δ 197.80, 159.70, 137.22, 132.56, 131.06, 130.00, 127.76, 127.03, 124.60, 119.66, 105.70, 55.36, 26.49.

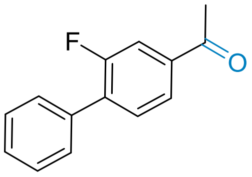

1-(2-Fluoro-[1,1'-biphenyl]-4-yl)ethan-1-one (42)[21]

White powder (40%, 48.8 mg), purified by silica gel column chromatography (Hexane: Ethyl Acetate = 10: 1).

^{1}H NMR (400 MHz, $CDCl_3$) δ 7.81 (dd, J = 8.0, 1.7 Hz, 1H), 7.74 (dd, J = 11.2, 1.7 Hz, 1H), 7.61 – 7.52 (m, 3H), 7.51 – 7.39 (m, 3H), 2.63 (s, 3H).

^{13}C NMR (101 MHz, $CDCl_3$) δ 196.49 (d, J_{C-F} = 1.9 Hz), 159.68 (d, J_{C-F} = 249.9 Hz), 137.81 (d, J_{C-F} = 6.4 Hz), 134.67 (d, J_{C-F} = 1.0 Hz), 133.80 (d, J_{C-F} = 13.7 Hz), 130.93 (d, J_{C-F} = 3.4 Hz), 129.00 (d, J_{C-F} = 3.1 Hz), 128.61, 128.50, 124.33 (d, J_{C-F} = 3.5 Hz), 115.91 (d, J_{C-F} = 24.1 Hz), 26.65.

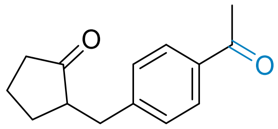

2-(4-Acetylbenzyl)cyclopentan-1-one (43)[22]

Colourless oil (79%, 97.2 mg), purified by silica gel column chromatography (Hexane: Ethyl Acetate = 5: 1).

^{1}H NMR (400 MHz, $CDCl_3$) δ 7.91 (d, *J* = 8.2 Hz, 2H), 7.29 (d, *J* = 8.2 Hz, 2H), 3.22 (dd, *J* = 13.9, 4.3 Hz, 1H), 2.70 – 2.63 (m, 1H), 2.61 (s, 3H), 2.43 – 2.33 (m, 2H), 2.19 – 2.07 (m, 2H), 2.04-1.96 (m, 1H), 1.84-1.72 (m, 1H), 1.62 – 1.46 (m, 1H).

^{13}C NMR (101 MHz, $CDCl_3$) δ 219.49, 197.70, 145.76, 135.30, 129.05, 128.51, 50.58, 37.96, 35.47, 29.04, 26.48, 20.43.

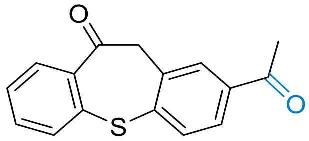

2-Acetyldibenzo[*b*,*f*]thiepin-10(11*H*)-one (44)[21]

Yellow powder (44%, 65.6 mg), purified by silica gel column chromatography (Hexane: Ethyl Acetate = 5: 1).

^{1}H NMR (400 MHz, $CDCl_3$) δ 8.20 (d, *J* = 7.9 Hz, 1H), 7.99 (s, 1H), 7.78 (d, *J* = 8.1 Hz, 1H), 7.72 (d, *J* = 8.1 Hz, 1H), 7.60 (d, *J* = 7.8 Hz, 1H), 7.45 (t, *J* = 7.5 Hz, 1H), 7.33 (t, *J* = 7.5 Hz, 1H), 4.41 (s, 2H), 2.59 (s, 3H).

^{13}C NMR (101 MHz, $CDCl_3$) δ 196.89, 190.65, 140.04, 139.01, 138.13, 137.90, 135.98, 132.75, 131.56, 131.42, 130.89, 129.09, 127.14, 126.88, 50.95, 26.69.

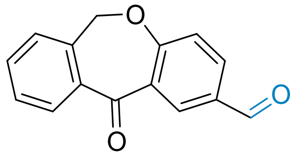

11-Oxo-6,11-dihydrodibenzo[*b*,*e*]oxepine-2-carbaldehyde (45)[23]

Yellow powder (47%, 63.0 mg), purified by silica gel column chromatography (Hexane: Ethyl Acetate = 5: 1).

^{1}H NMR (400 MHz, $CDCl_3$) δ 10.00 (s, 1H), 8.73 (d, *J* = 2.1 Hz, 1H), 8.03 (dd, *J* = 8.6, 2.1 Hz, 1H), 7.89 (d, *J* = 7.6, 1H), 7.61 (t, *J* = 7.6, 1H), 7.51 (t, *J* = 7.6, 1H), 7.41 (d, *J* = 7.4, 1H), 7.17 (d, *J* = 8.6 Hz, 1H), 5.29 (s, 2H).

^{13}C NMR (101 MHz, $CDCl_3$) δ 190.38, 190.17, 165.54, 140.32, 137.46, 134.51, 133.41, 133.20, 130.93, 129.73, 129.39, 128.14, 125.01, 122.16, 73.61.

7 NMR spectra of substrates

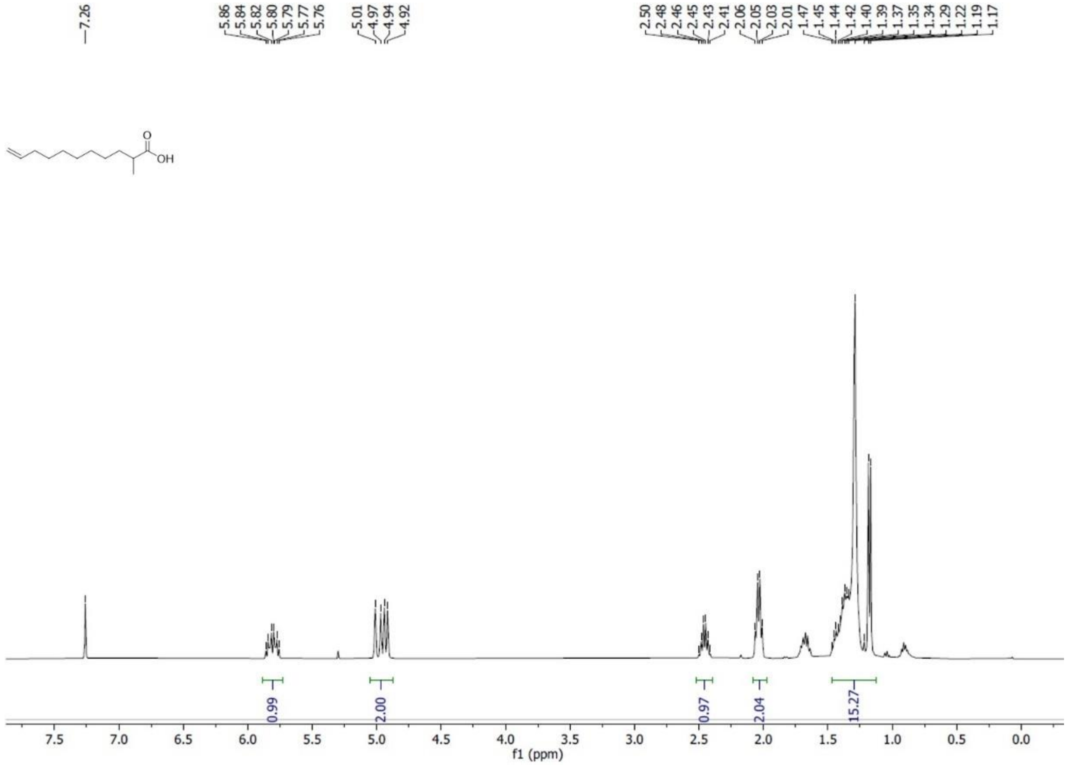

^{1}H NMR (400 MHz, $CDCl_3$)

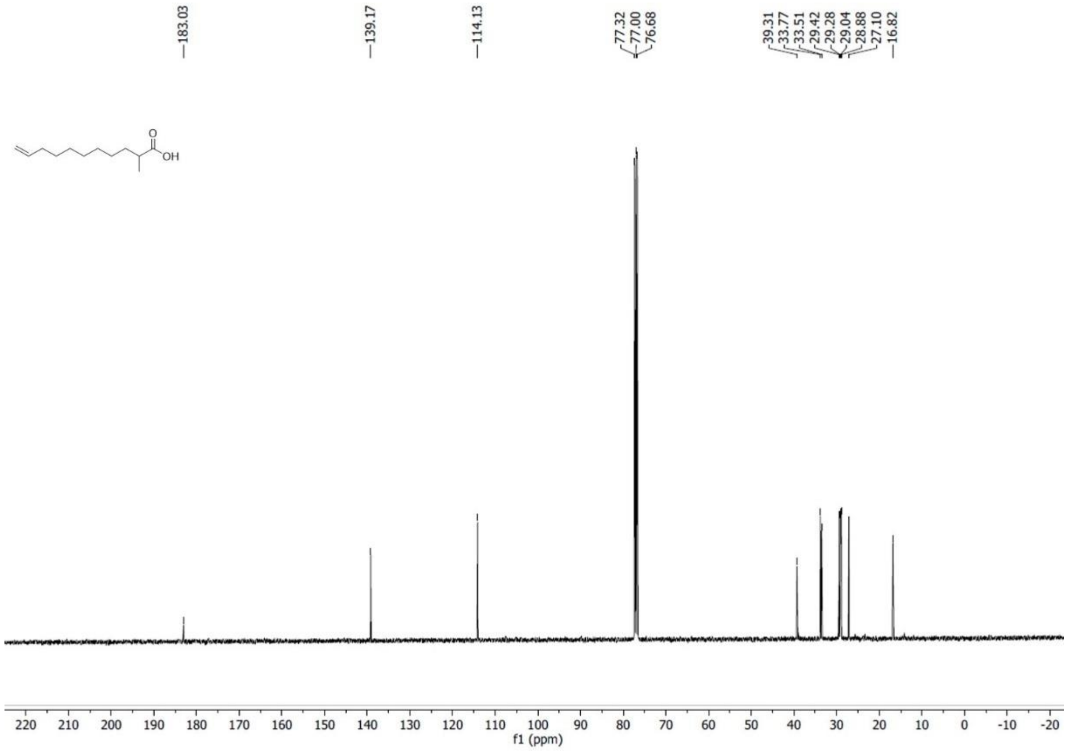

^{13}C NMR (101 MHz, $CDCl_3$)

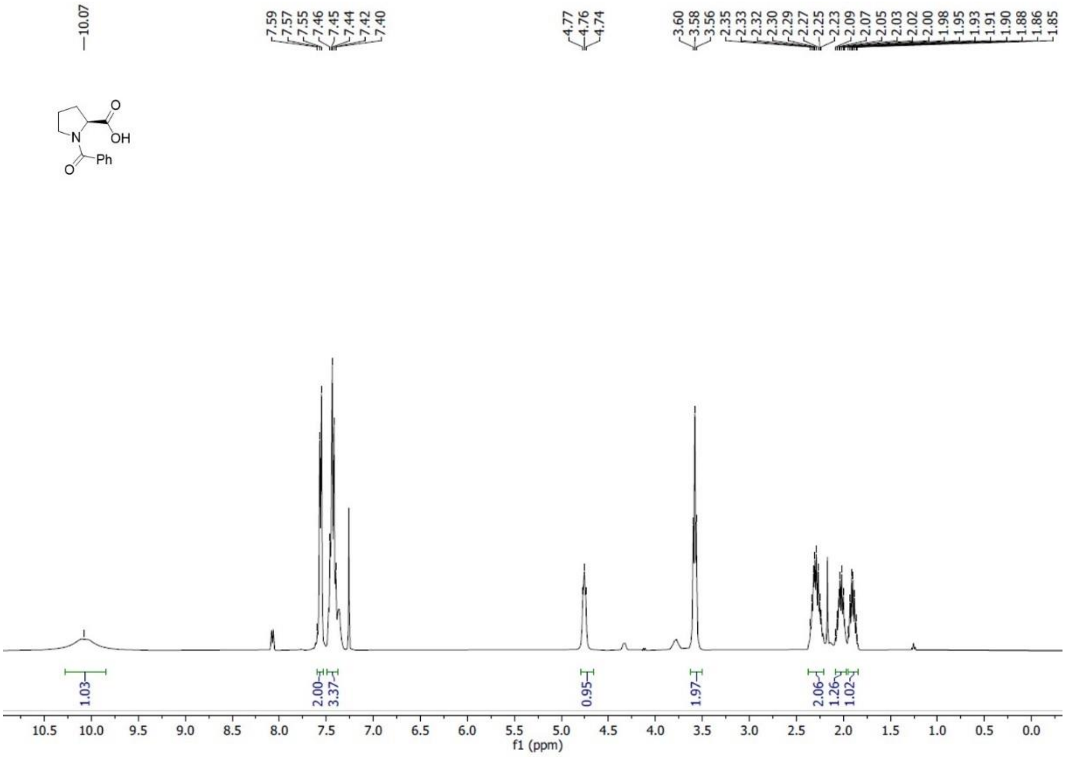

^{1}H NMR (400 MHz, $CDCl_3$)

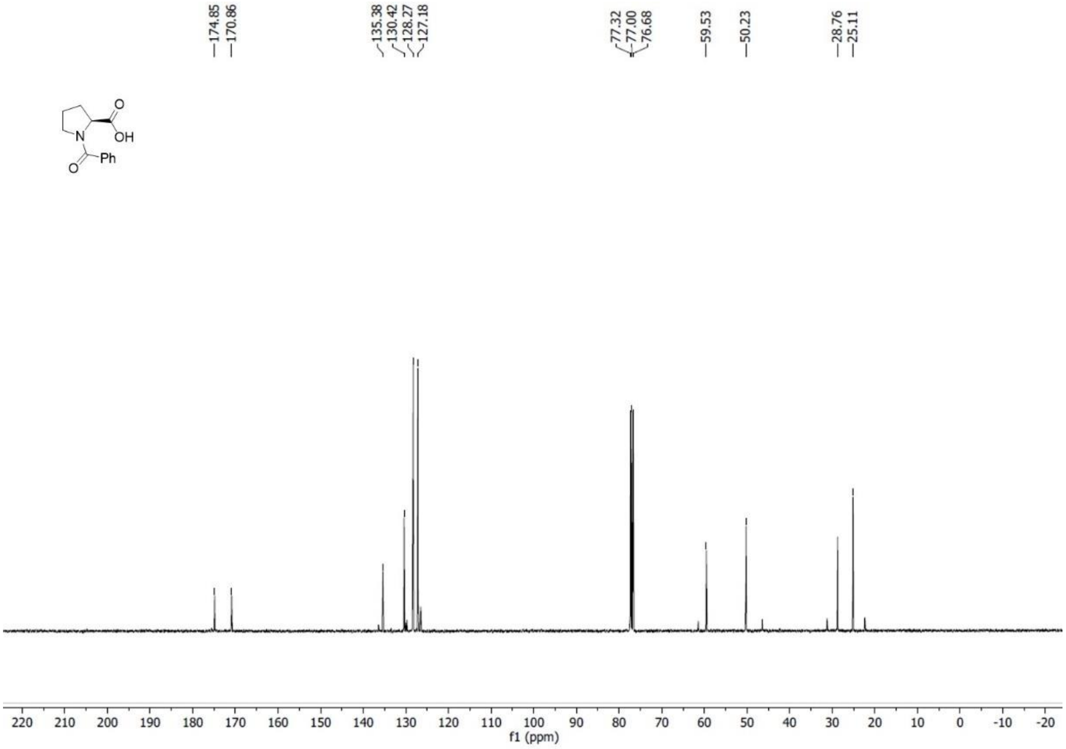

^{13}C NMR (101 MHz, $CDCl_3$)

8 NMR spectra of products

8.1 NMR spectra of peroxides

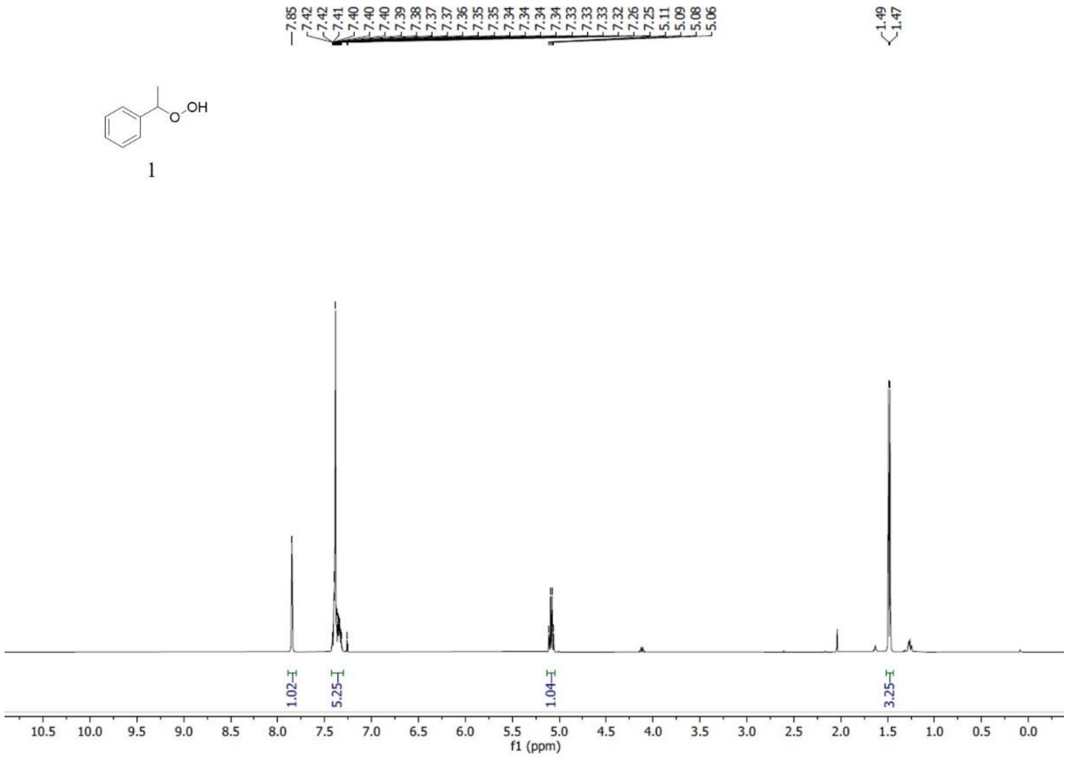

^{1}H NMR (400 MHz, $CDCl_3$)

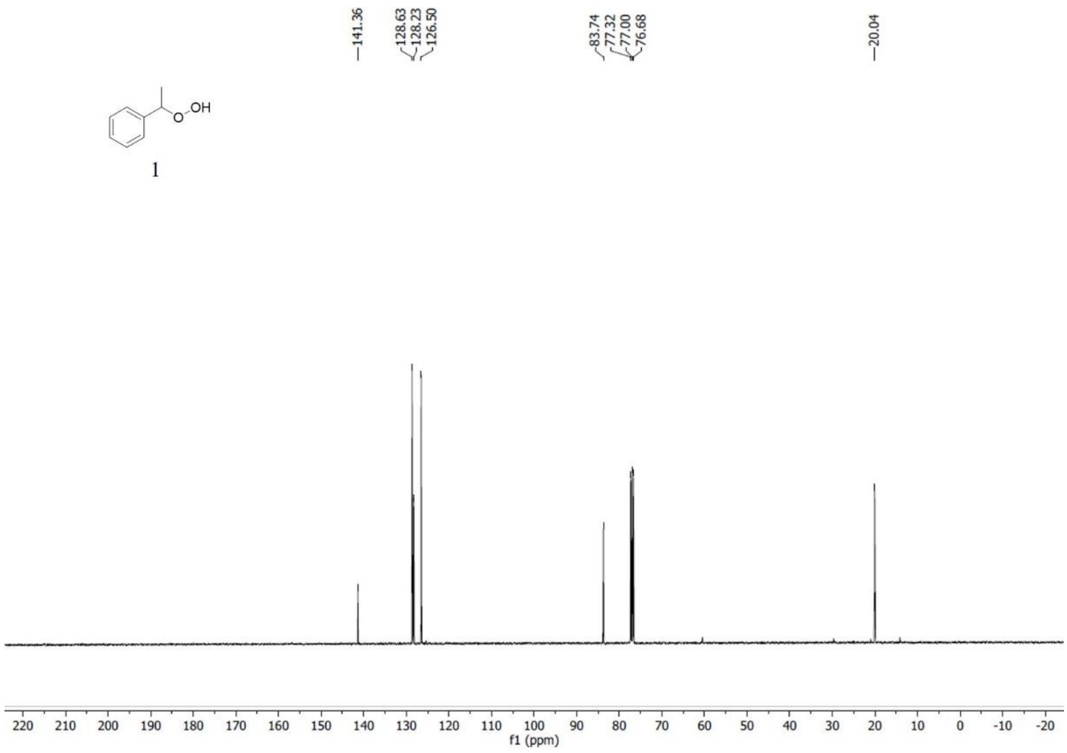

^{13}C NMR (101 MHz, $CDCl_3$)

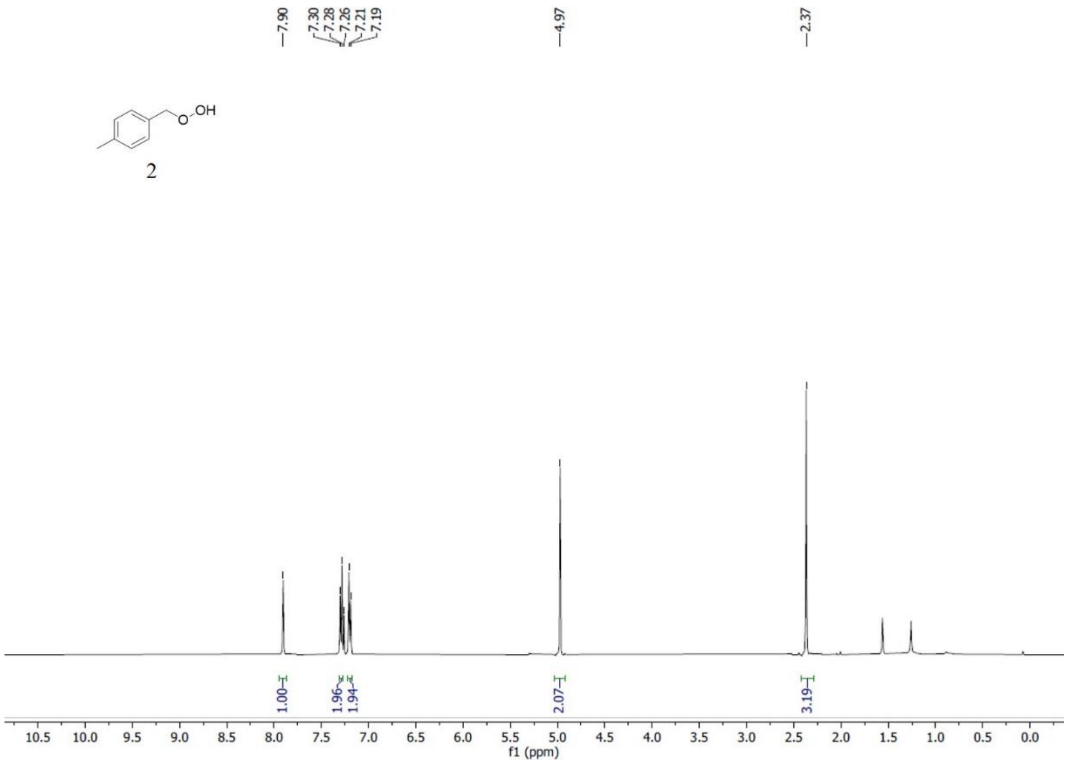

^{1}H NMR (400 MHz, $CDCl_3$)

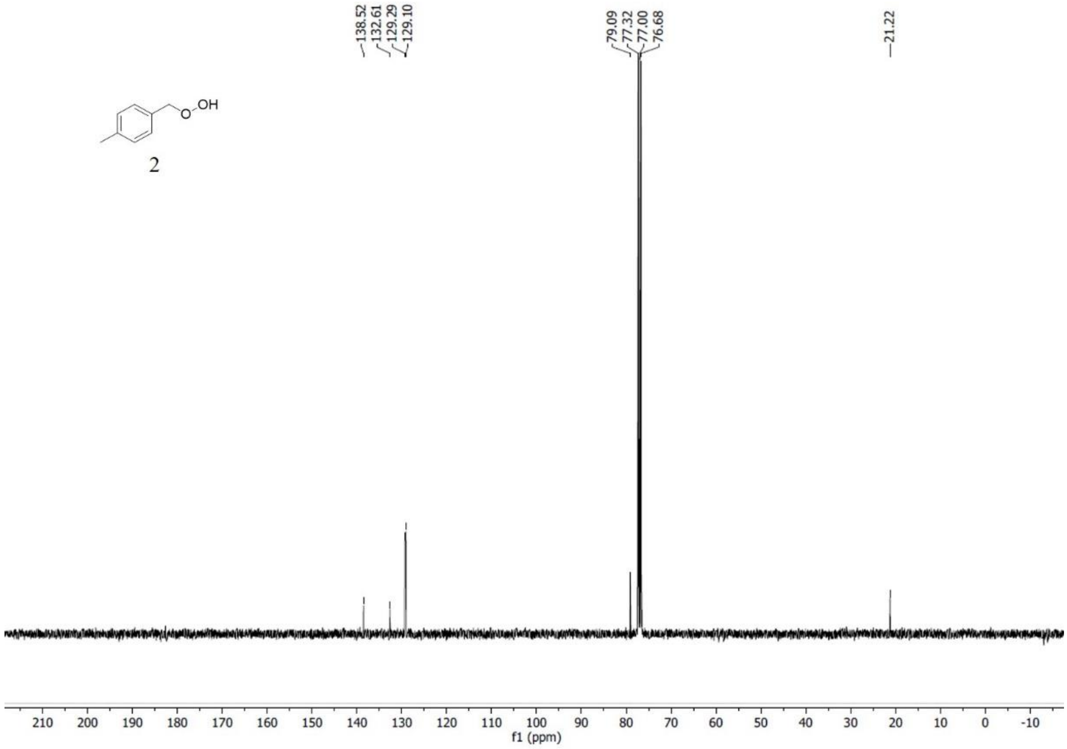

^{13}C NMR (101 MHz, $CDCl_3$)

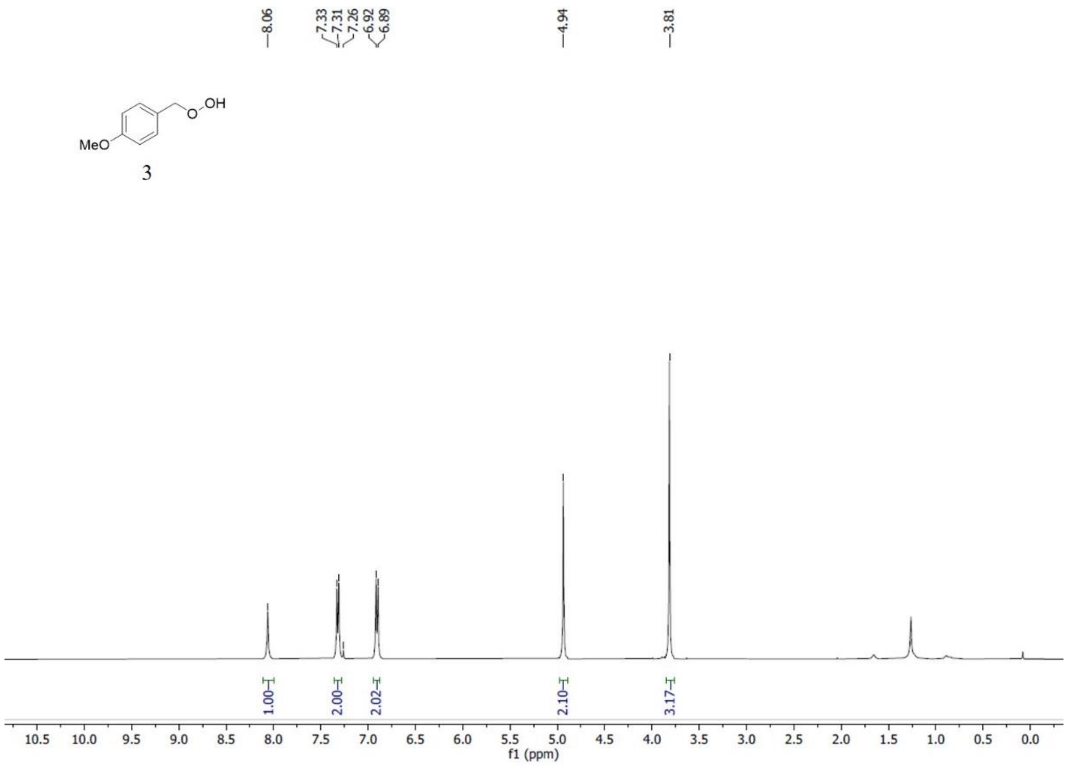

^{1}H NMR (400 MHz, $CDCl_3$)

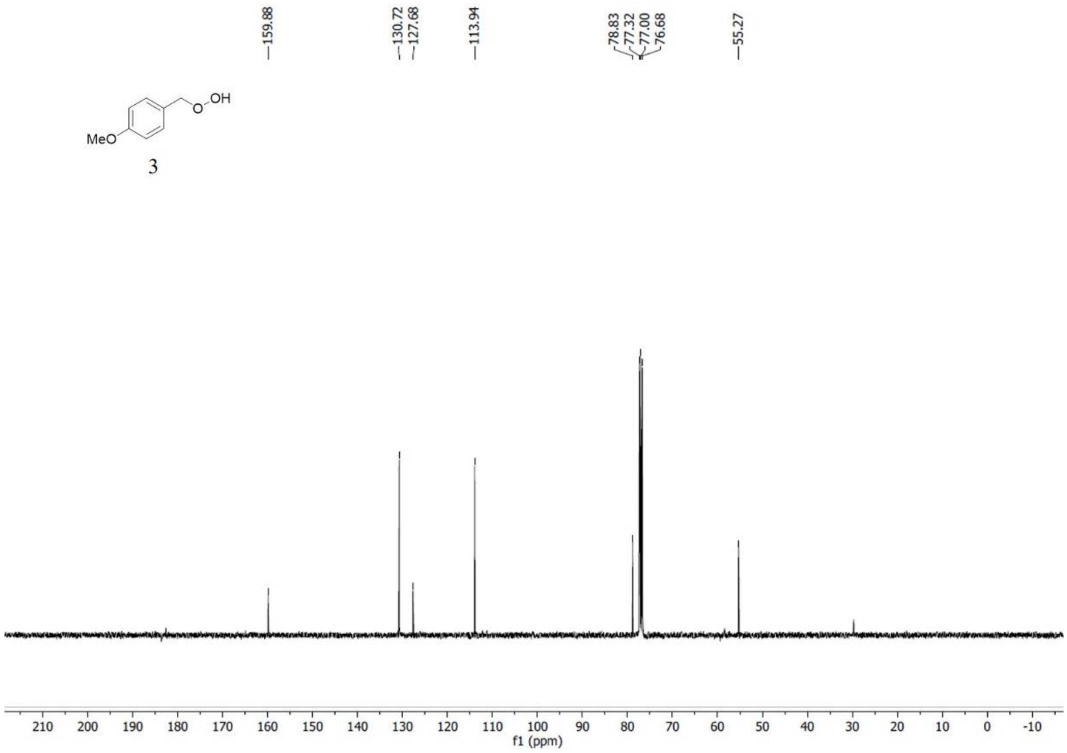

^{13}C NMR (101 MHz, $CDCl_3$)

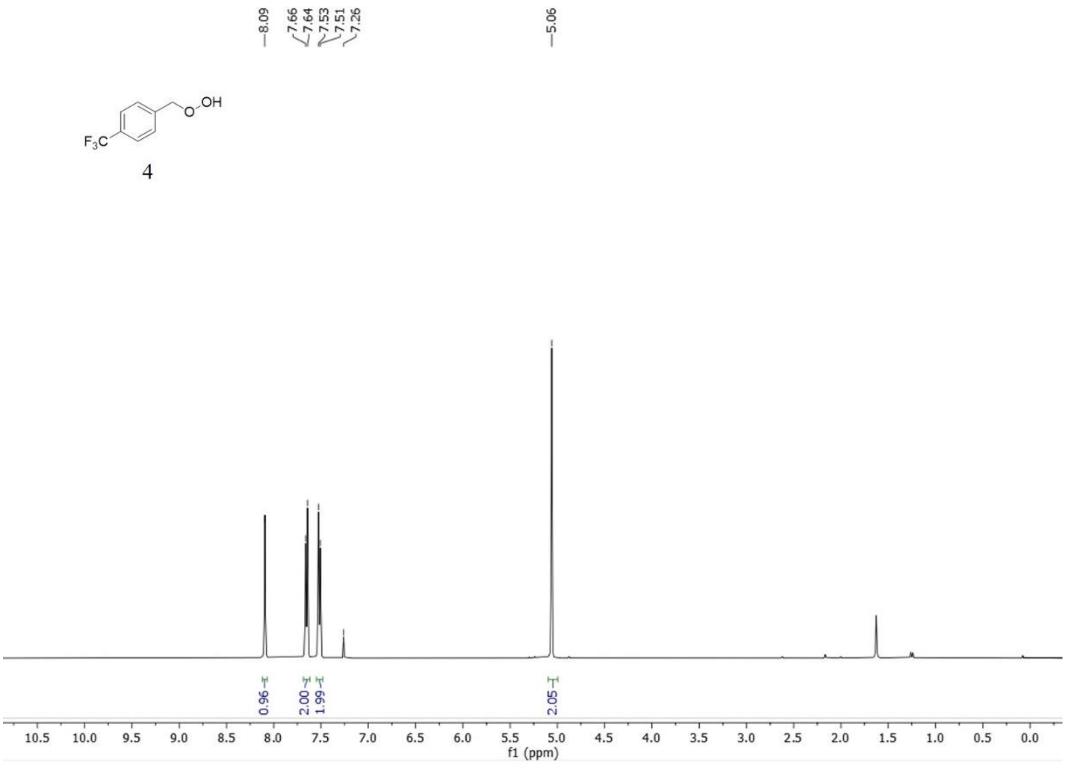

^{1}H NMR (400 MHz, $CDCl_3$)

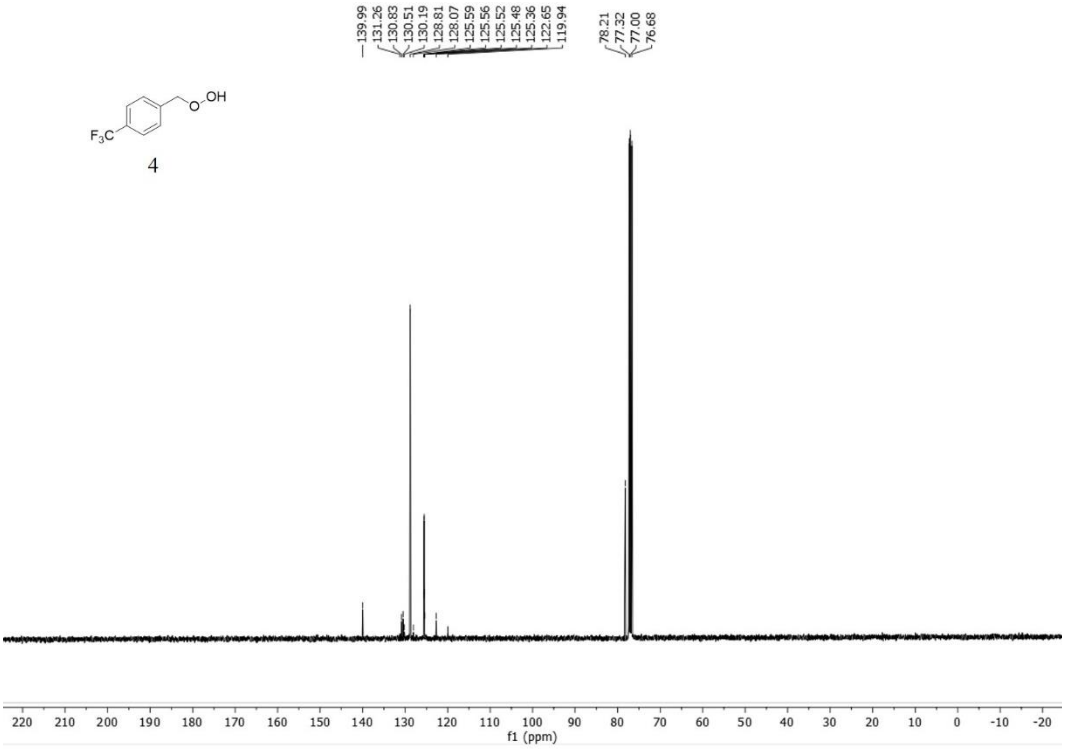

^{13}C NMR (101 MHz, $CDCl_3$)

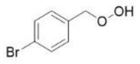

5

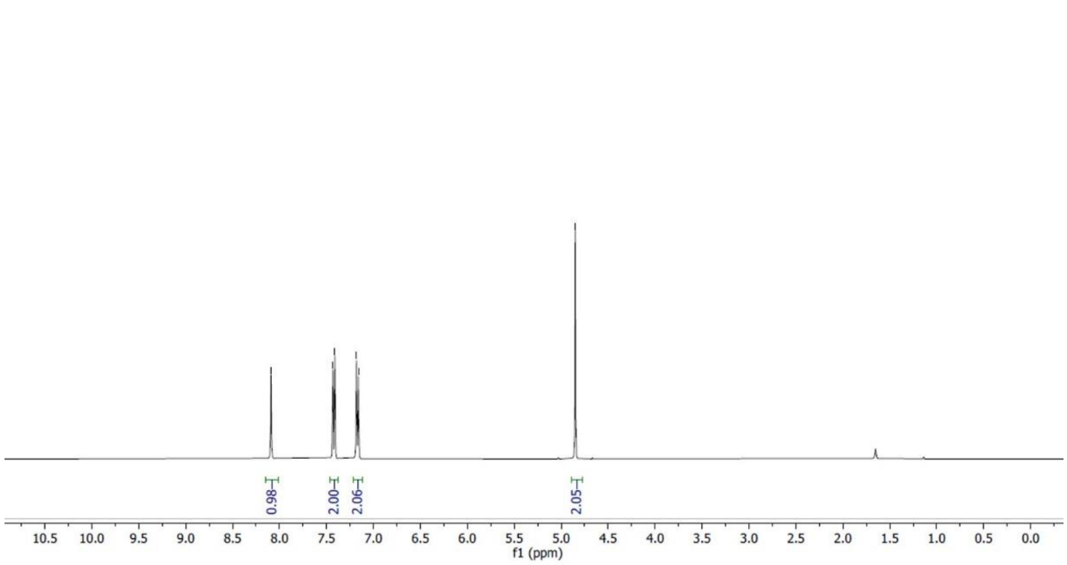

^{1}H NMR (400 MHz, $CDCl_3$)

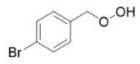

5

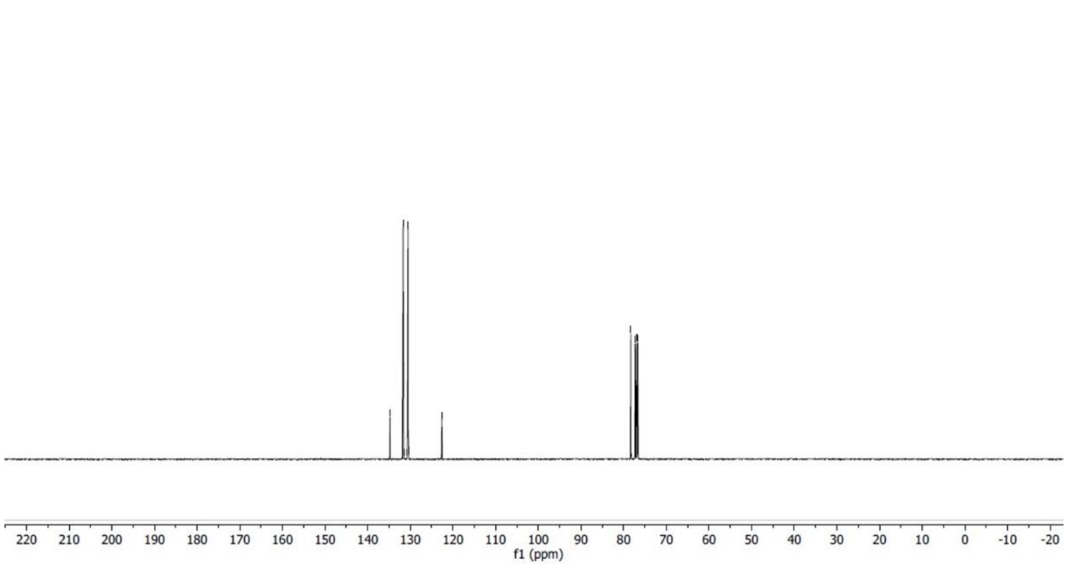

^{13}C NMR (101 MHz, $CDCl_3$)

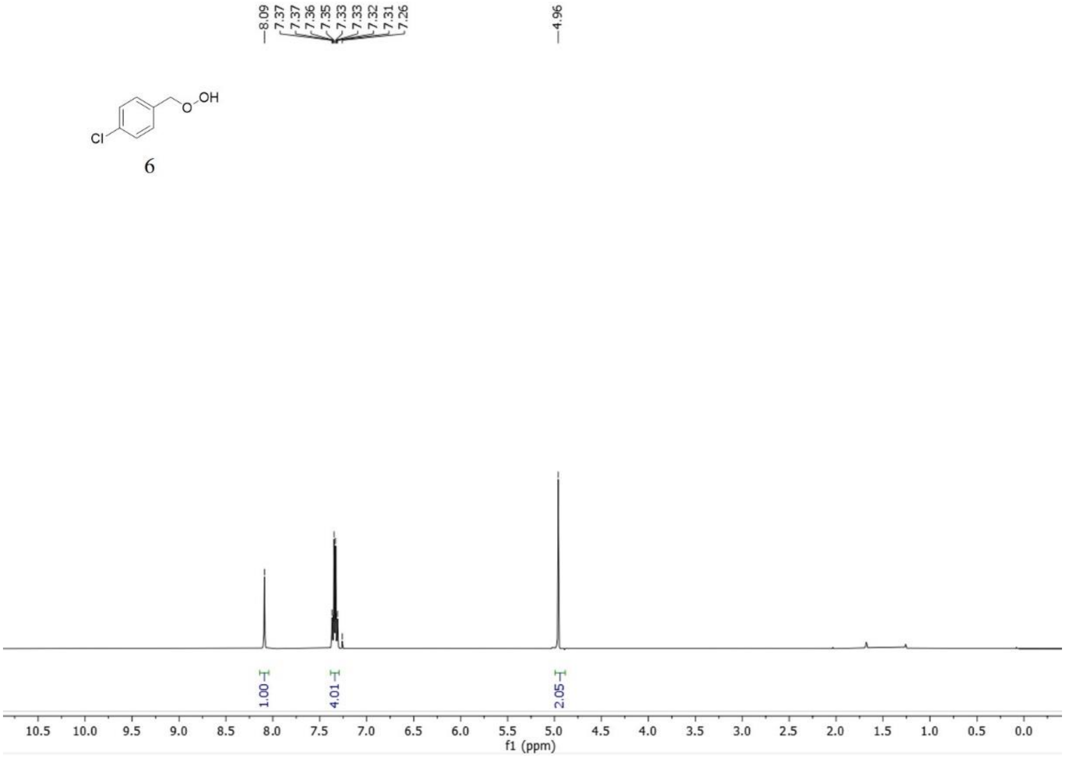

^{1}H NMR (400 MHz, $CDCl_3$)

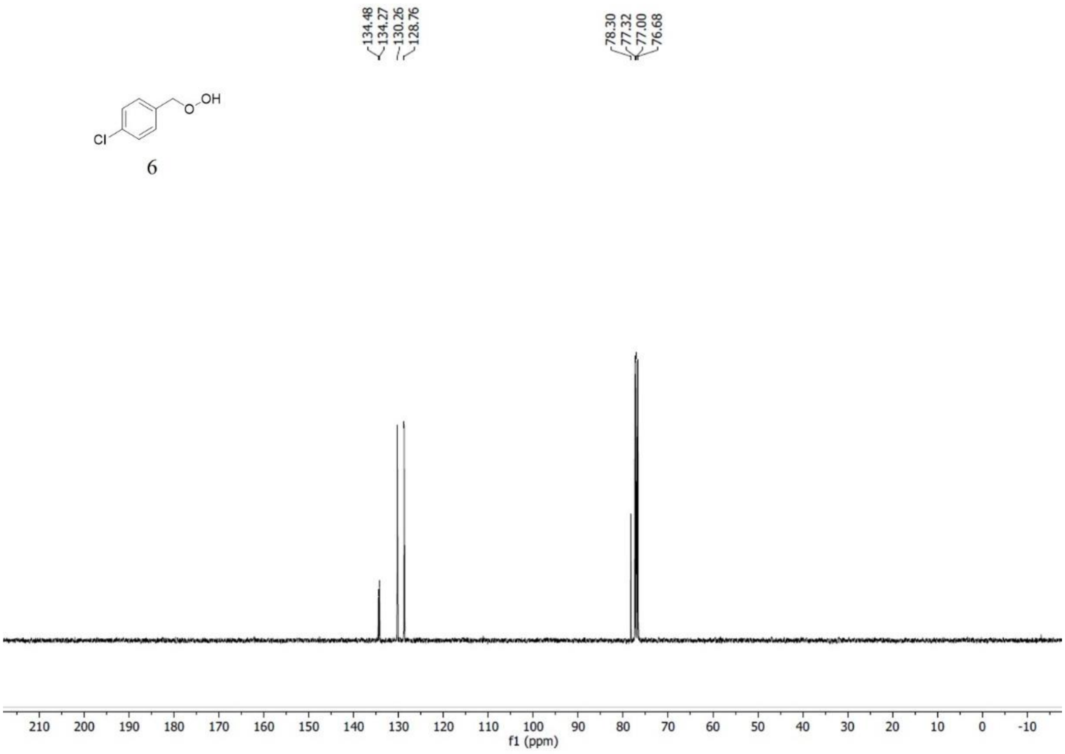

^{13}C NMR (101 MHz, $CDCl_3$)

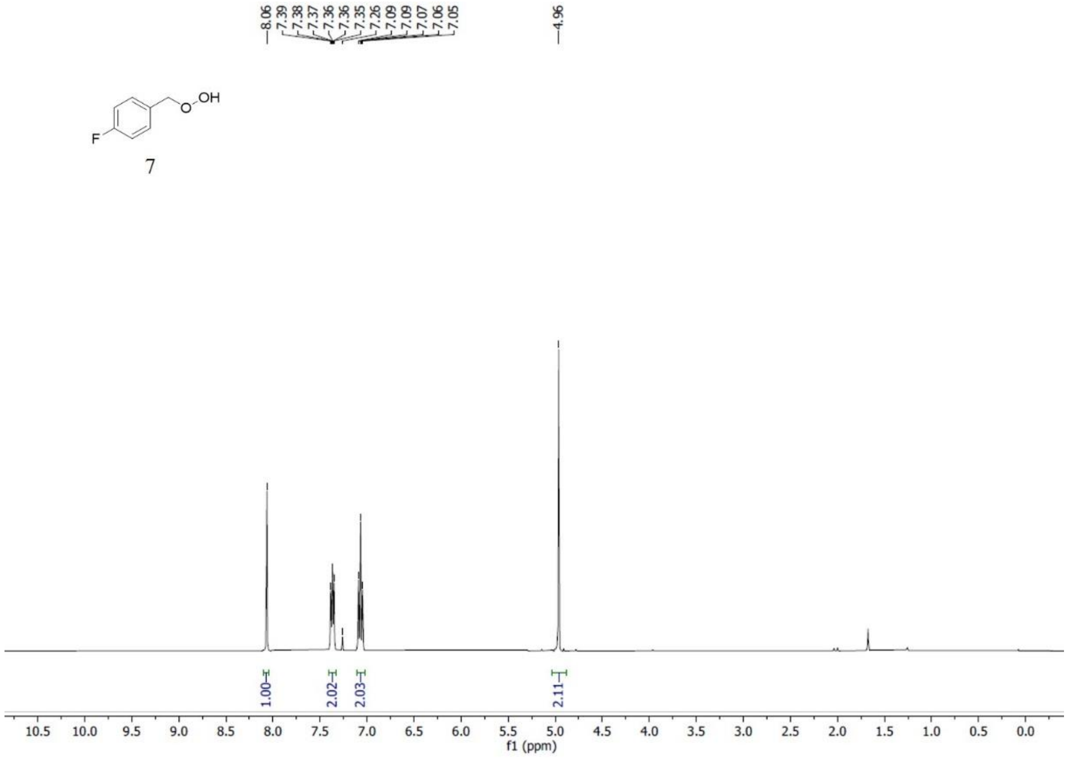

^{1}H NMR (400 MHz, $CDCl_3$)

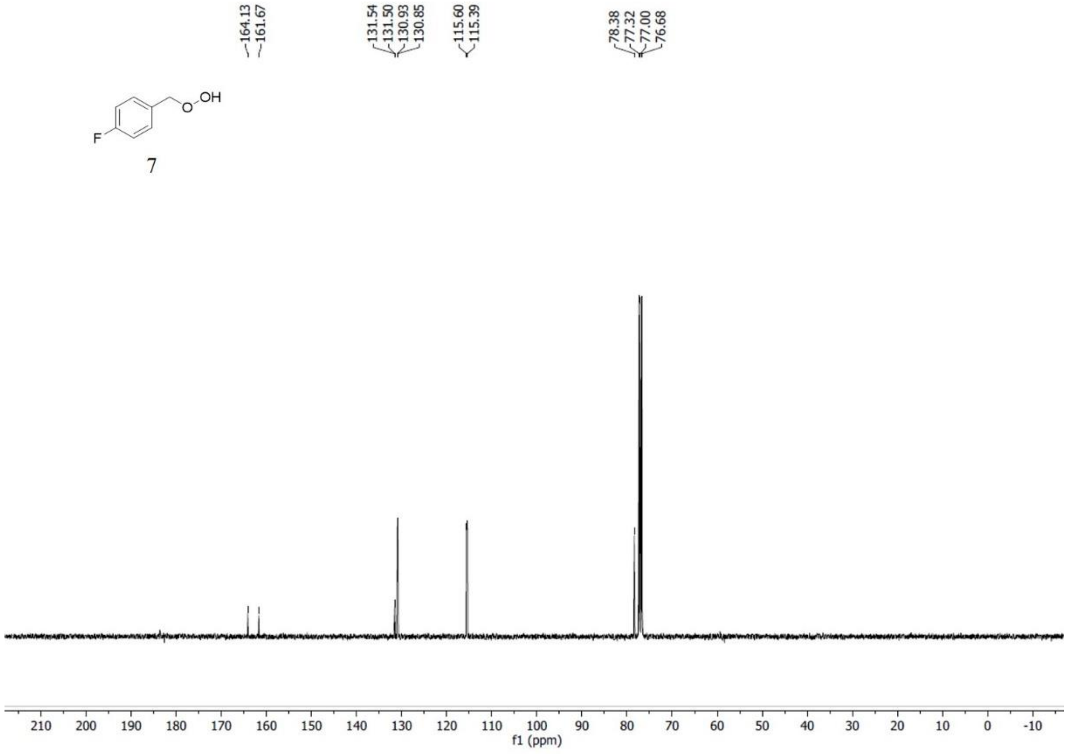

^{13}C NMR (101 MHz, $CDCl_3$)

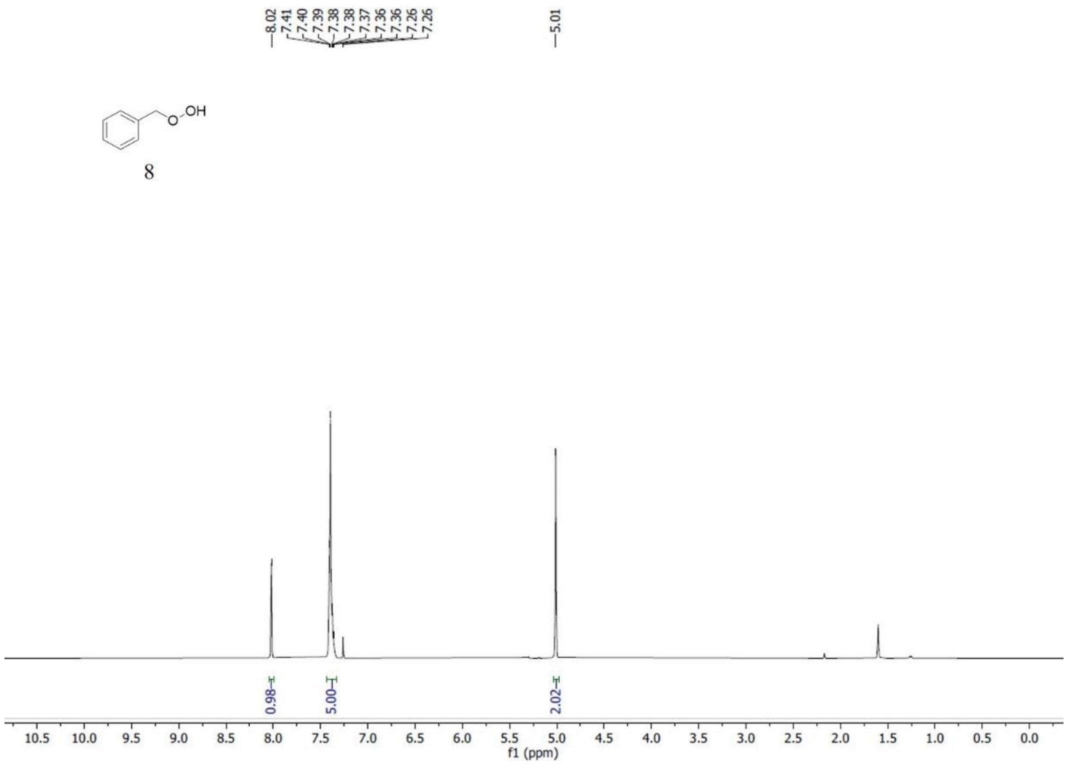

^{1}H NMR (400 MHz, $CDCl_3$)

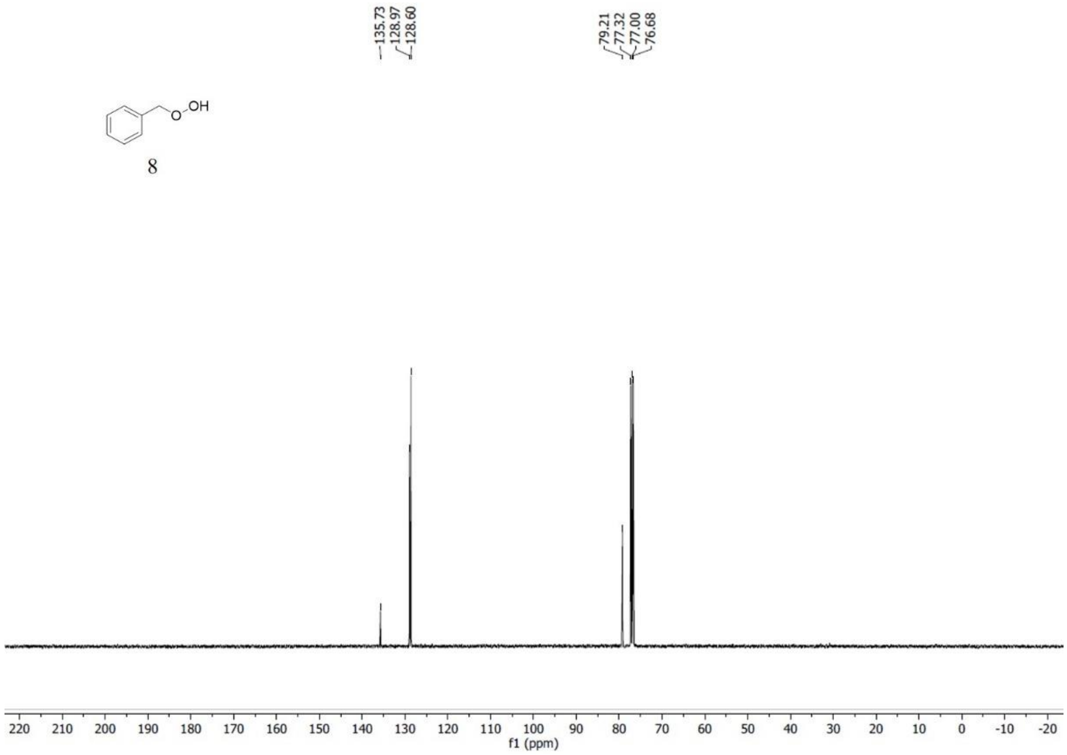

^{13}C NMR (101 MHz, $CDCl_3$)

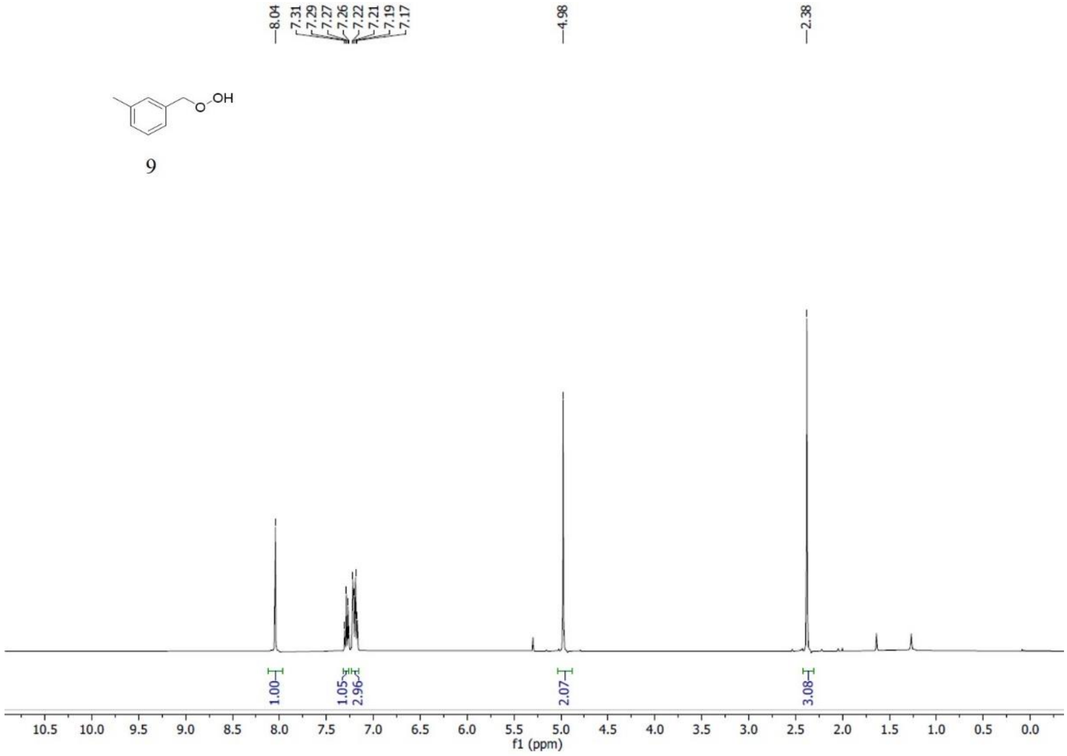

^{1}H NMR (400 MHz, $CDCl_3$)

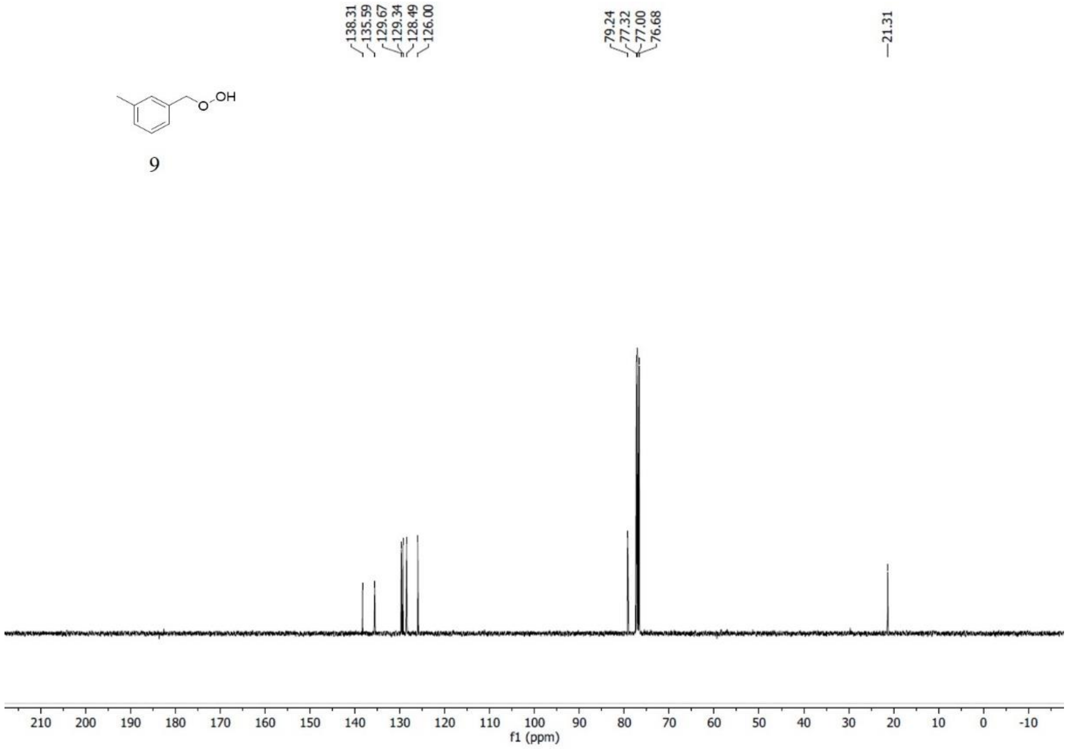

^{13}C NMR (101 MHz, $CDCl_3$)

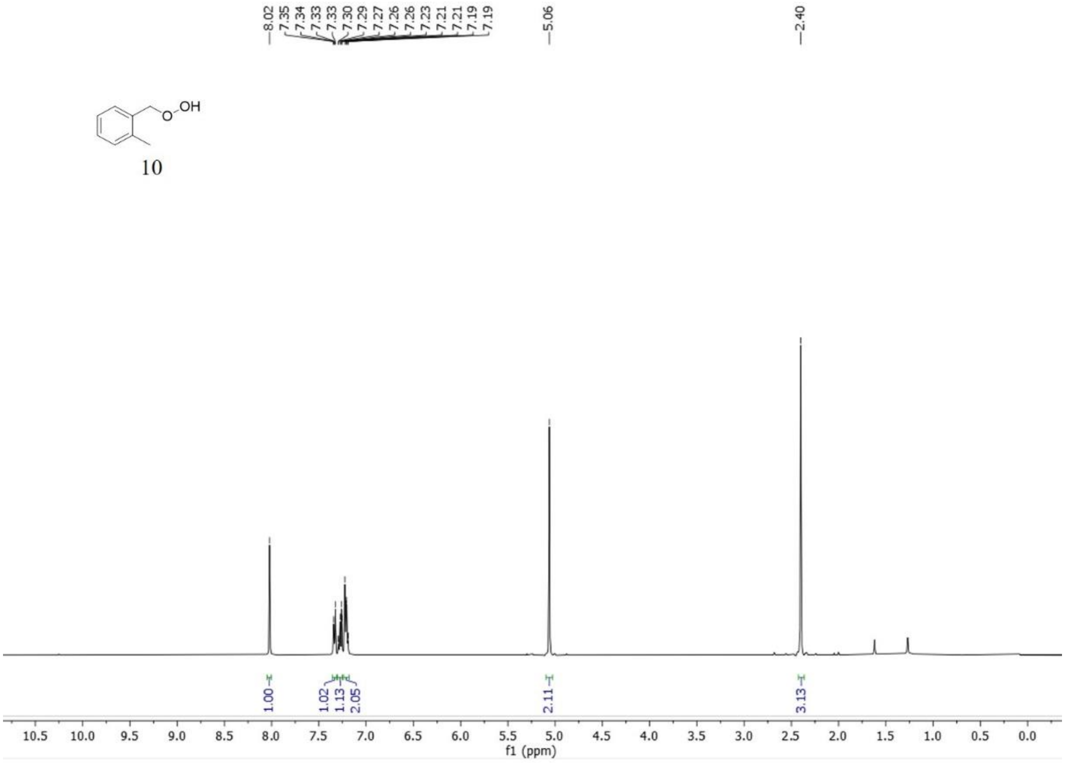

^{1}H NMR (400 MHz, $CDCl_3$)

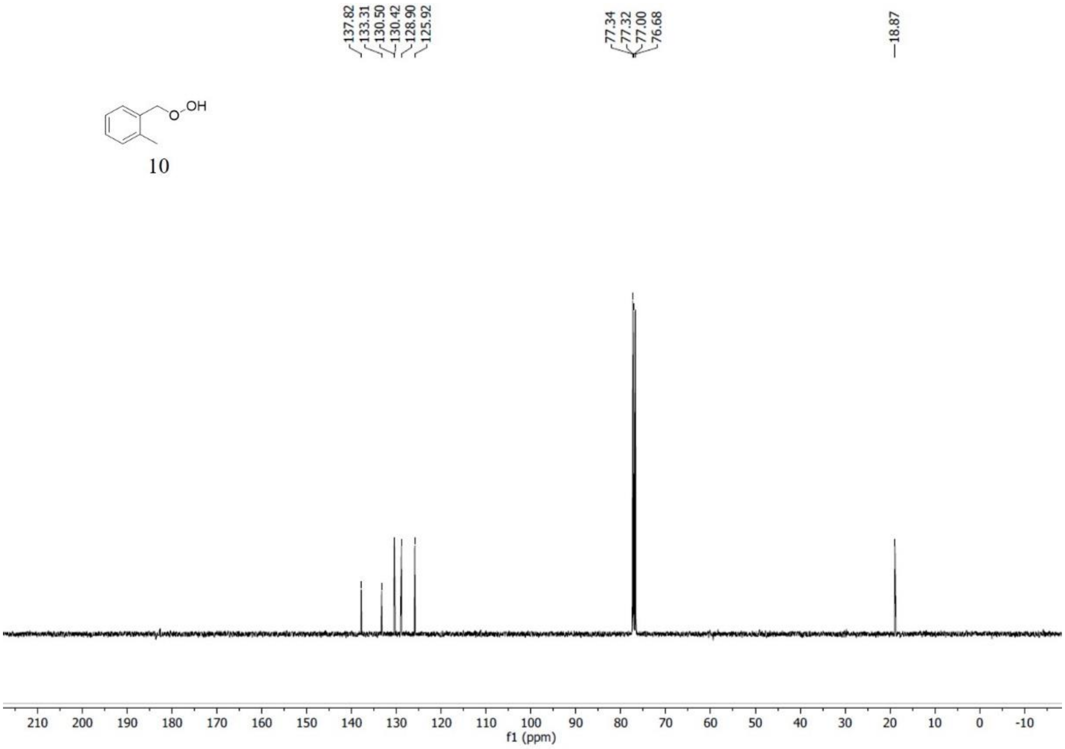

^{13}C NMR (101 MHz, $CDCl_3$)

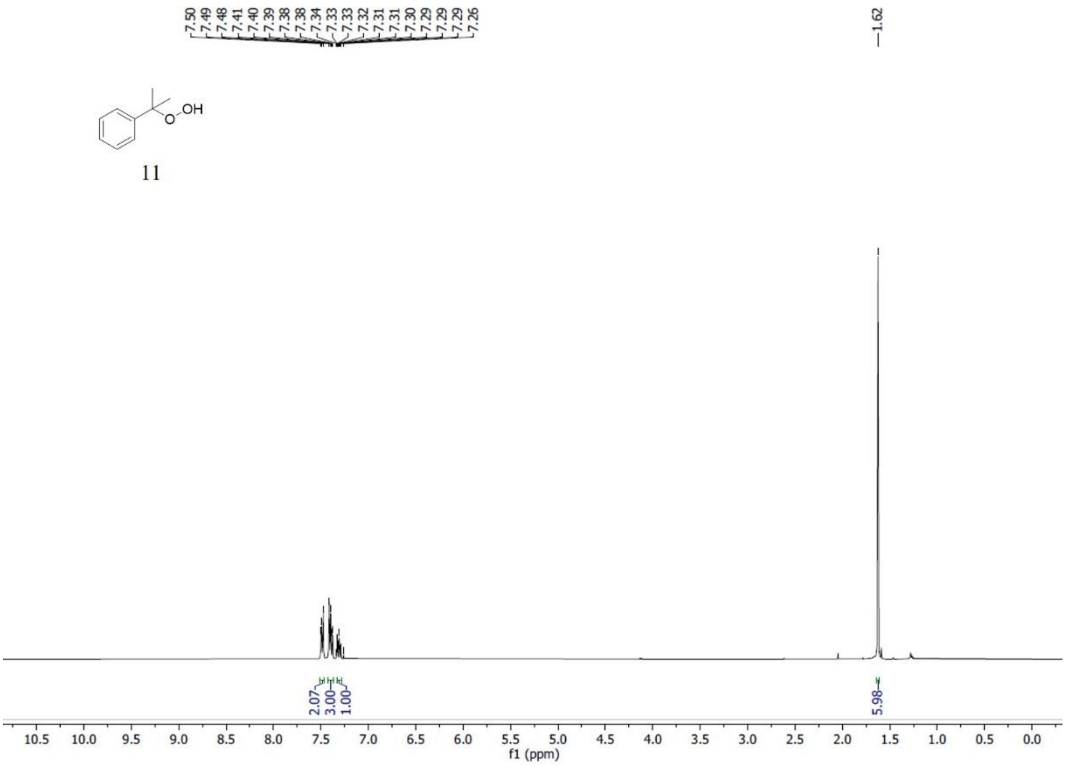

^{1}H NMR (400 MHz, $CDCl_3$)

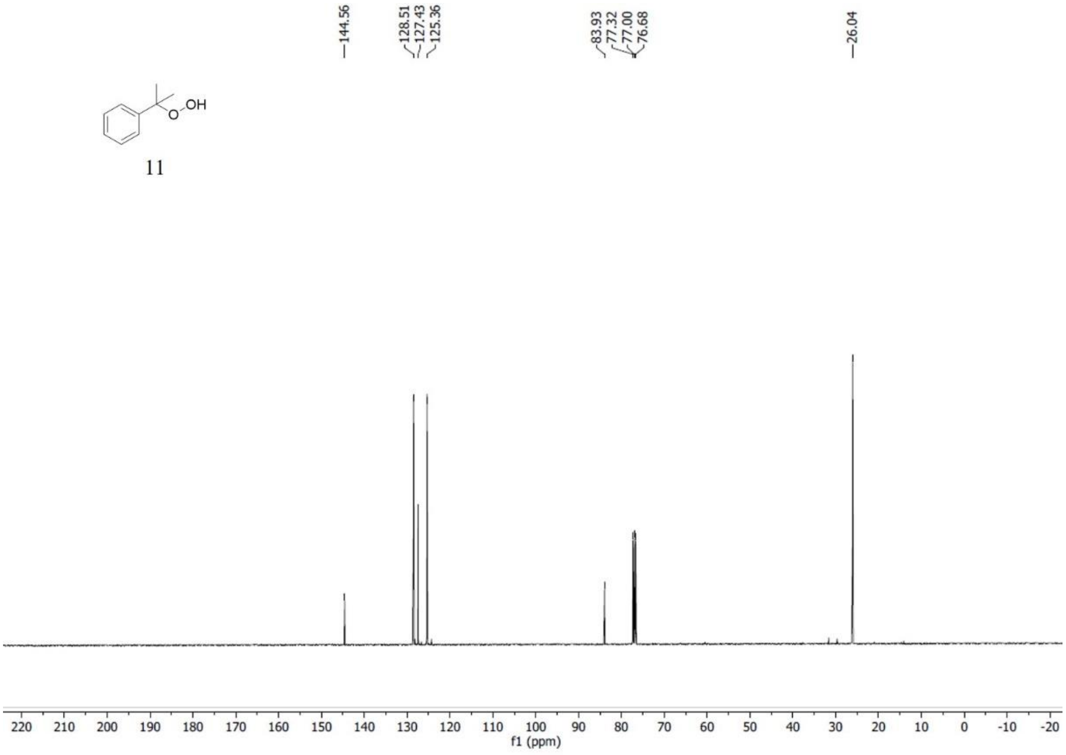

^{13}C NMR (101 MHz, $CDCl_3$)

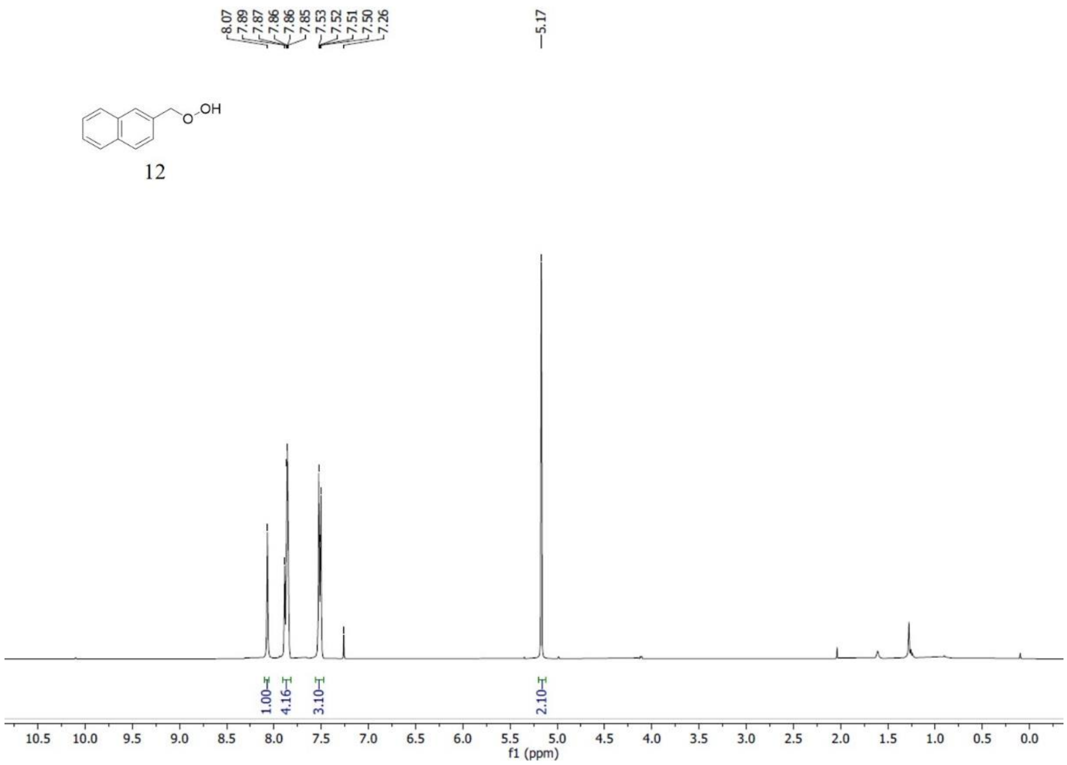

^{1}H NMR (400 MHz, $CDCl_3$)

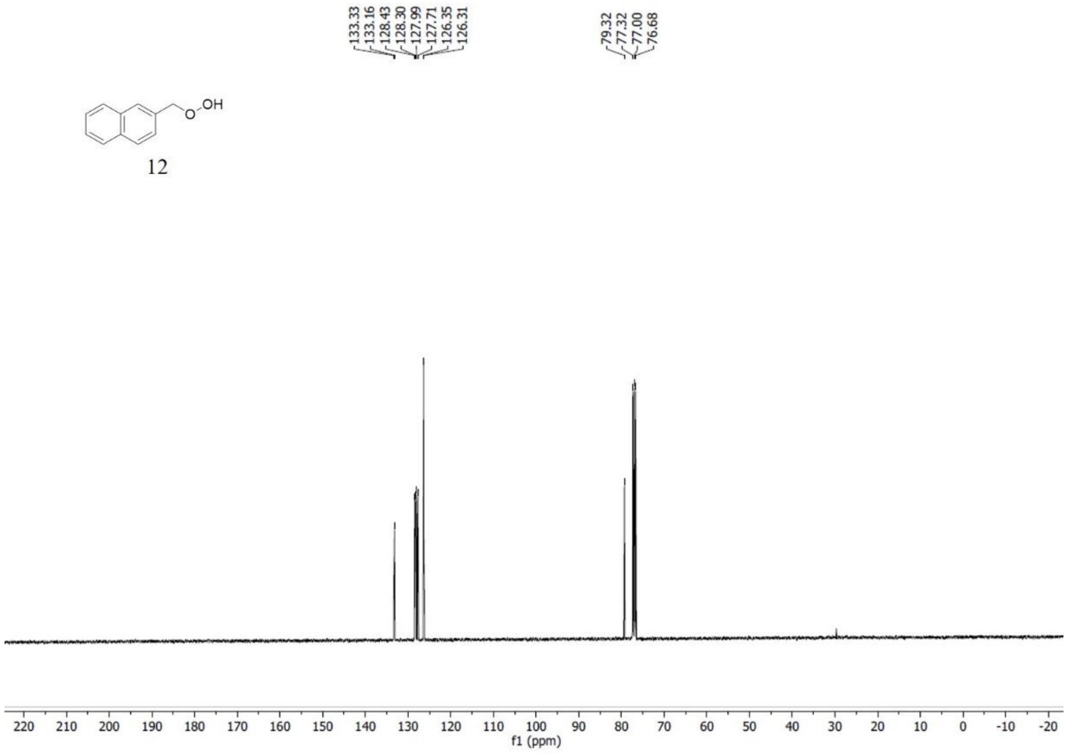

^{13}C NMR (101 MHz, $CDCl_3$)

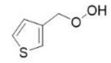

13

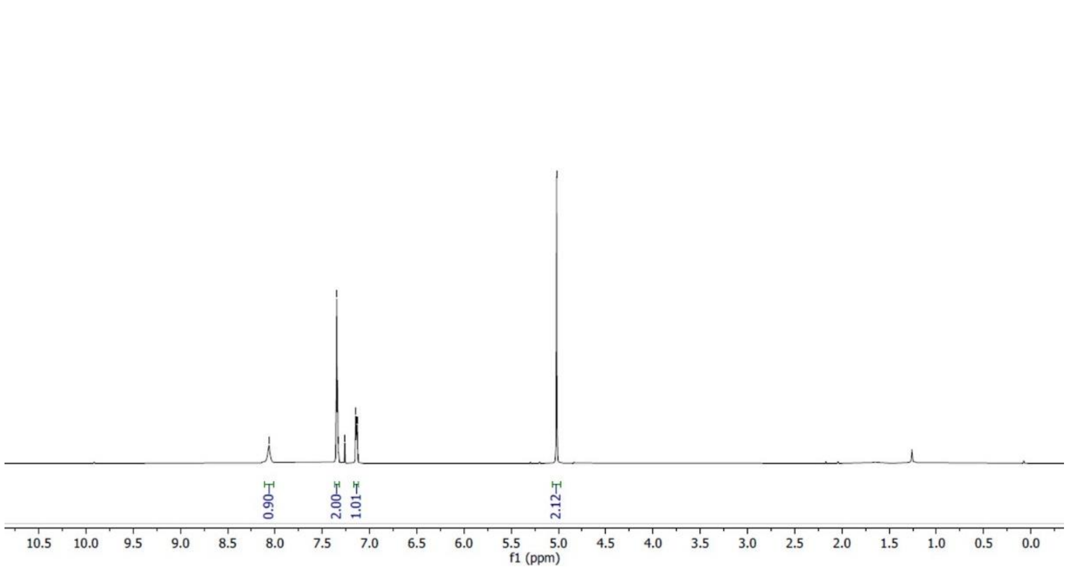

^{1}H NMR (400 MHz, $CDCl_3$)

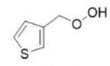

13

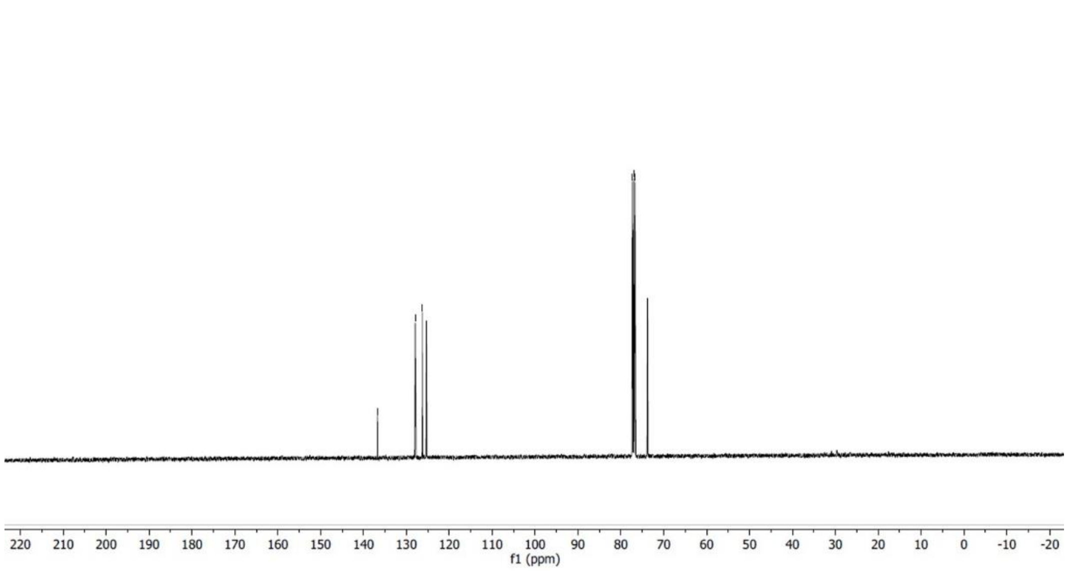

^{13}C NMR (101 MHz, $CDCl_3$)

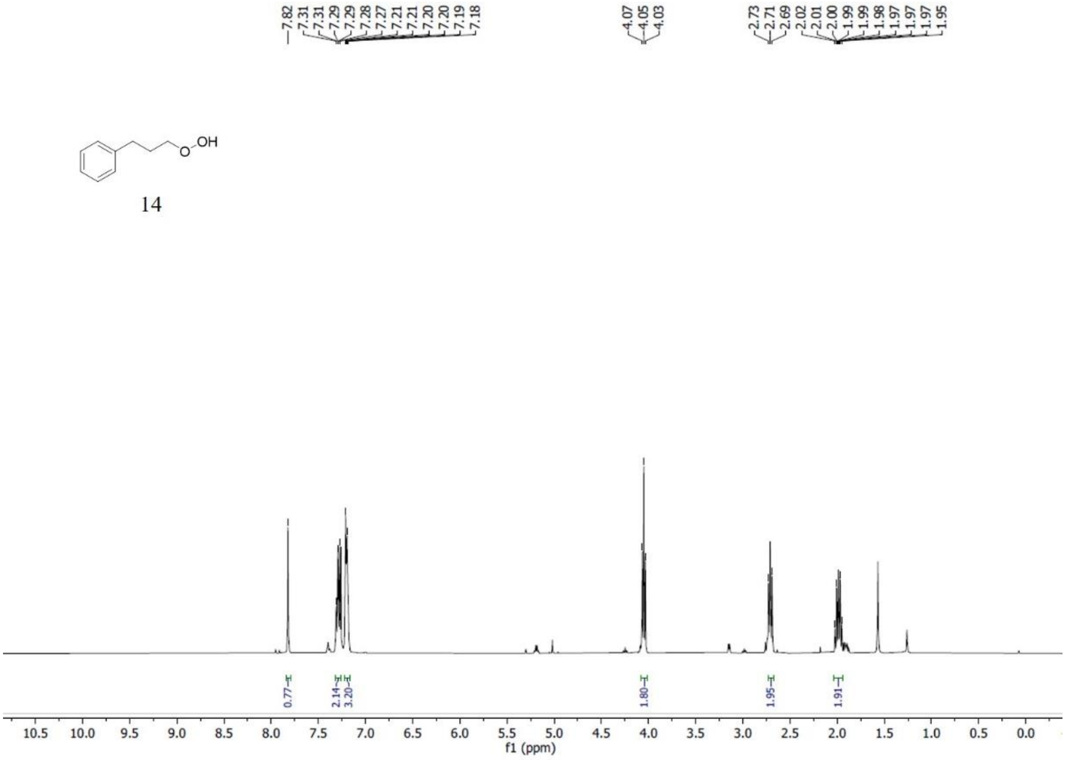

^1H NMR (400 MHz, $CDCl_3$)

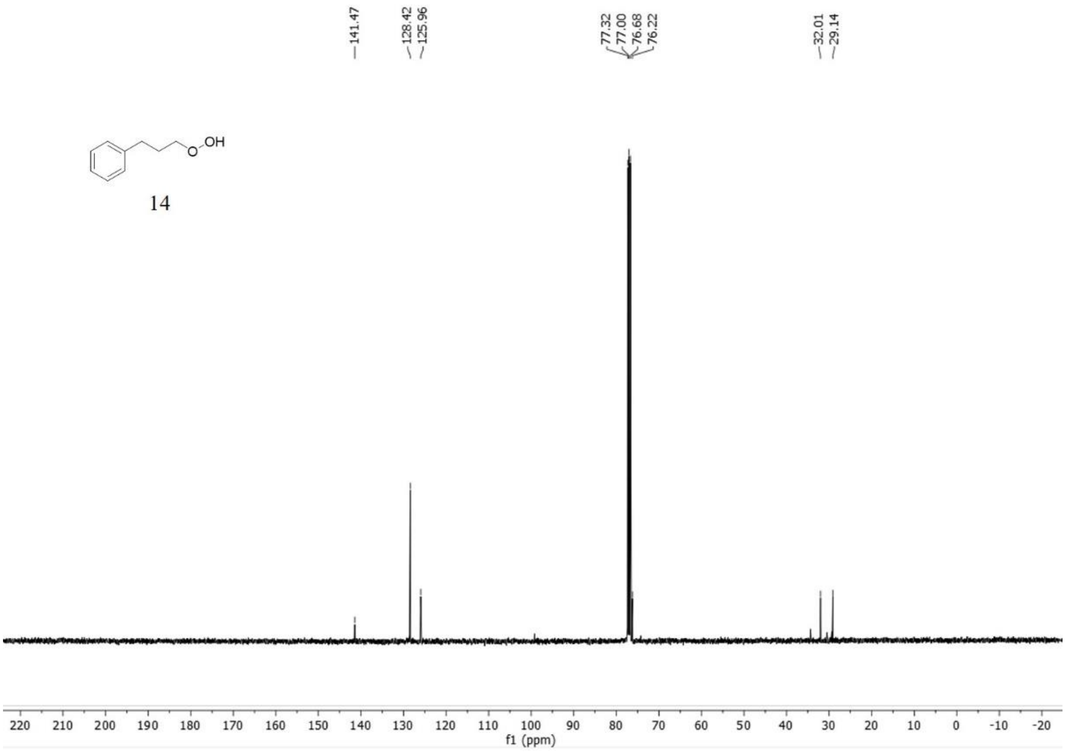

^{13}C NMR (101 MHz, $CDCl_3$)

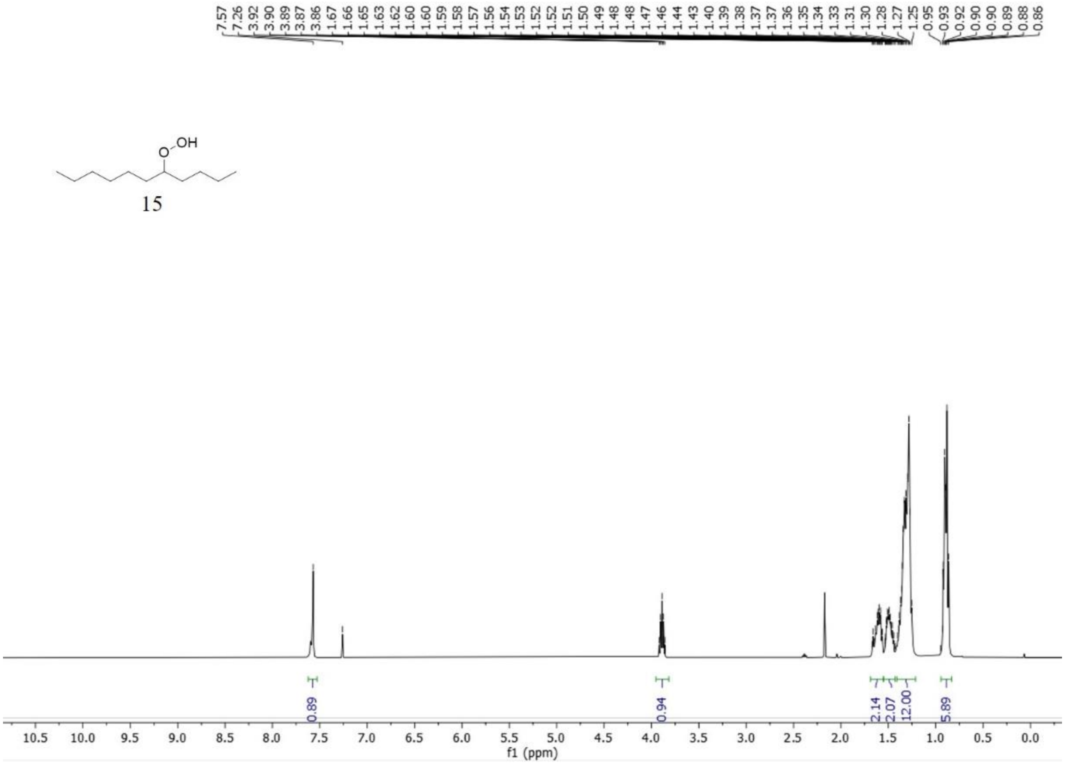

^{1}H NMR (400 MHz, $CDCl_3$)

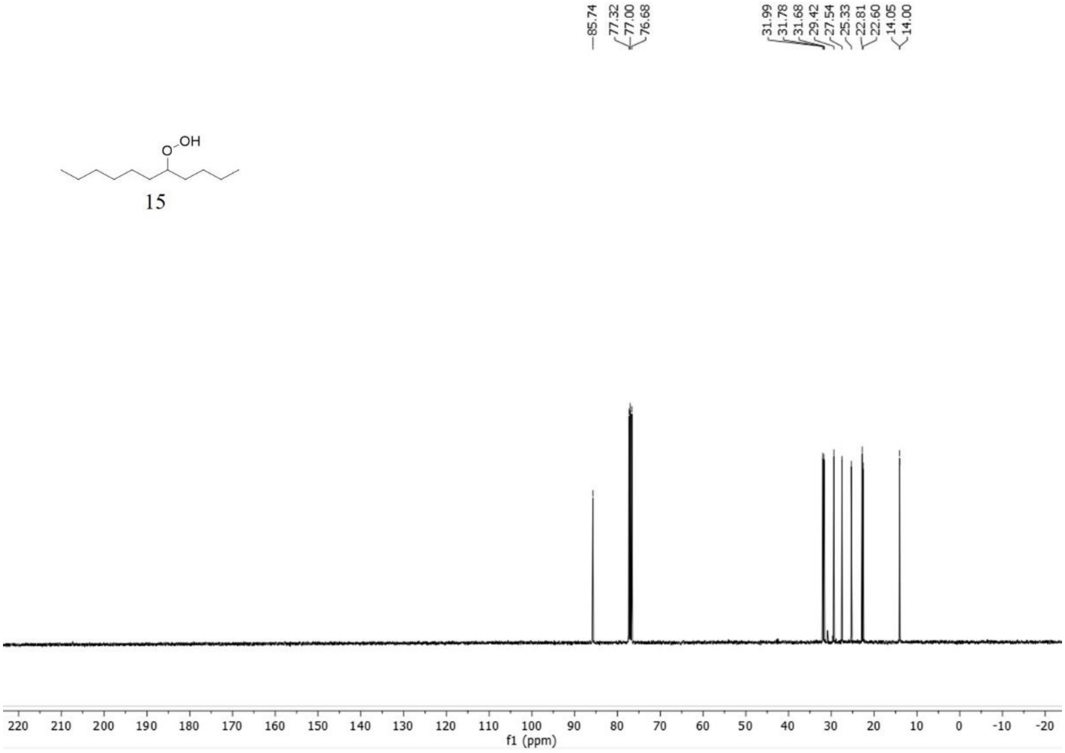

^{13}C NMR (101 MHz, $CDCl_3$)

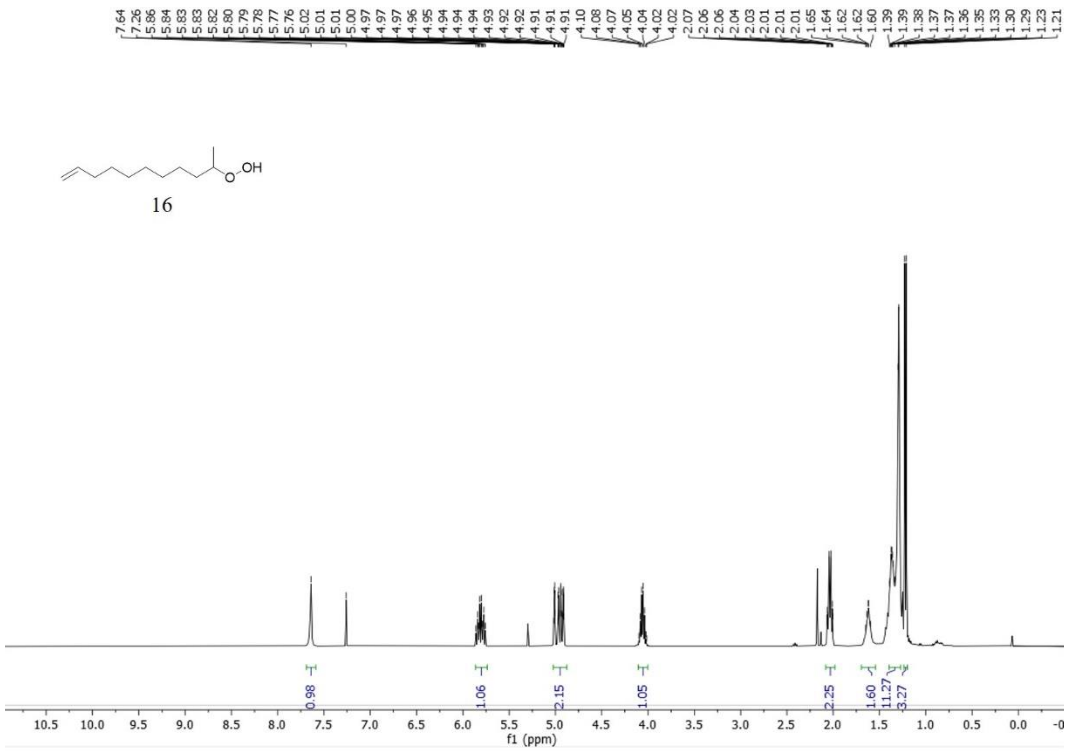

^{1}H NMR (400 MHz, $CDCl_3$)

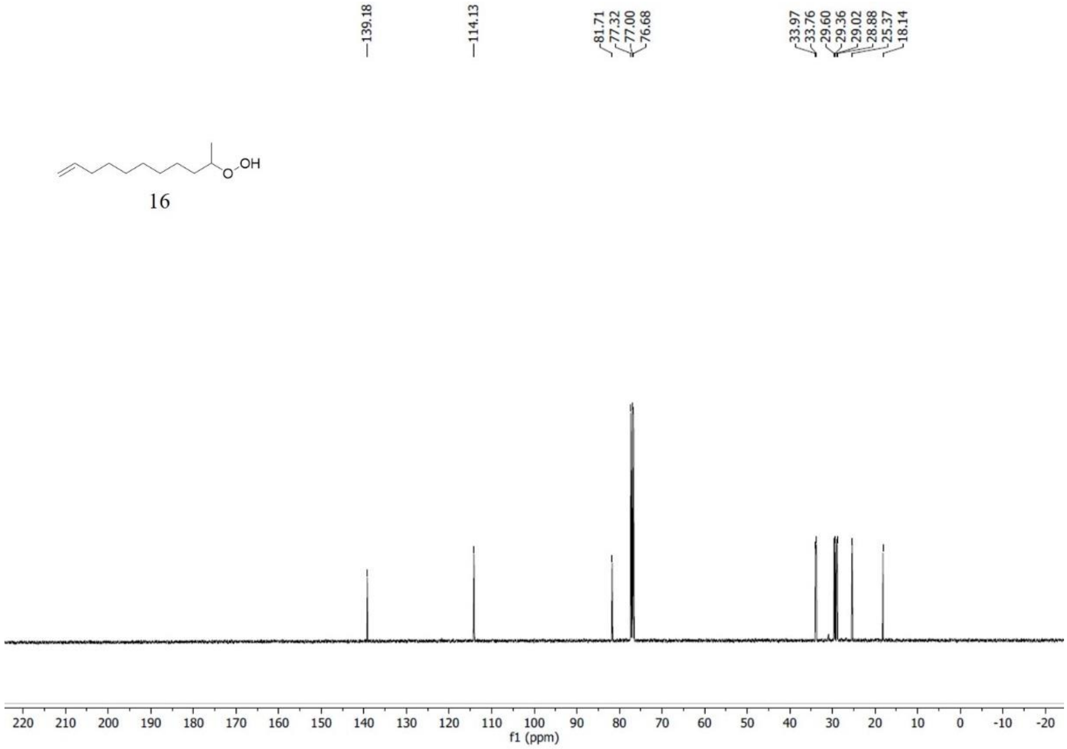

^{13}C NMR (101 MHz, $CDCl_3$)

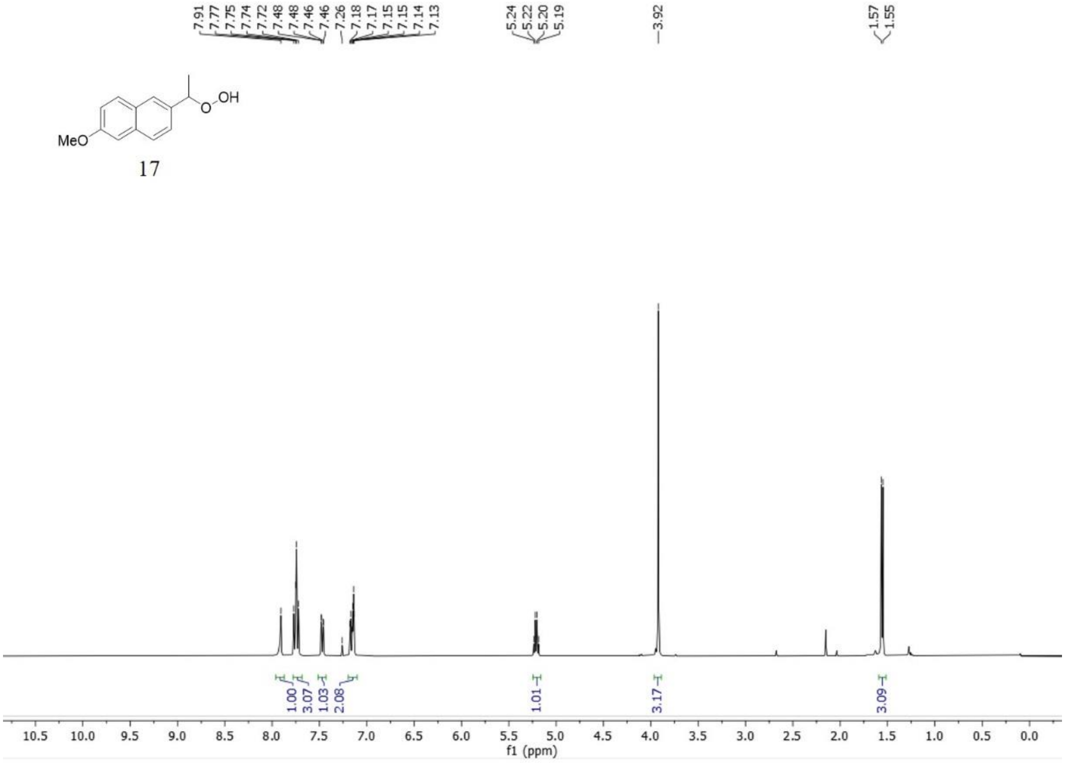

^{1}H NMR (400 MHz, $CDCl_3$)

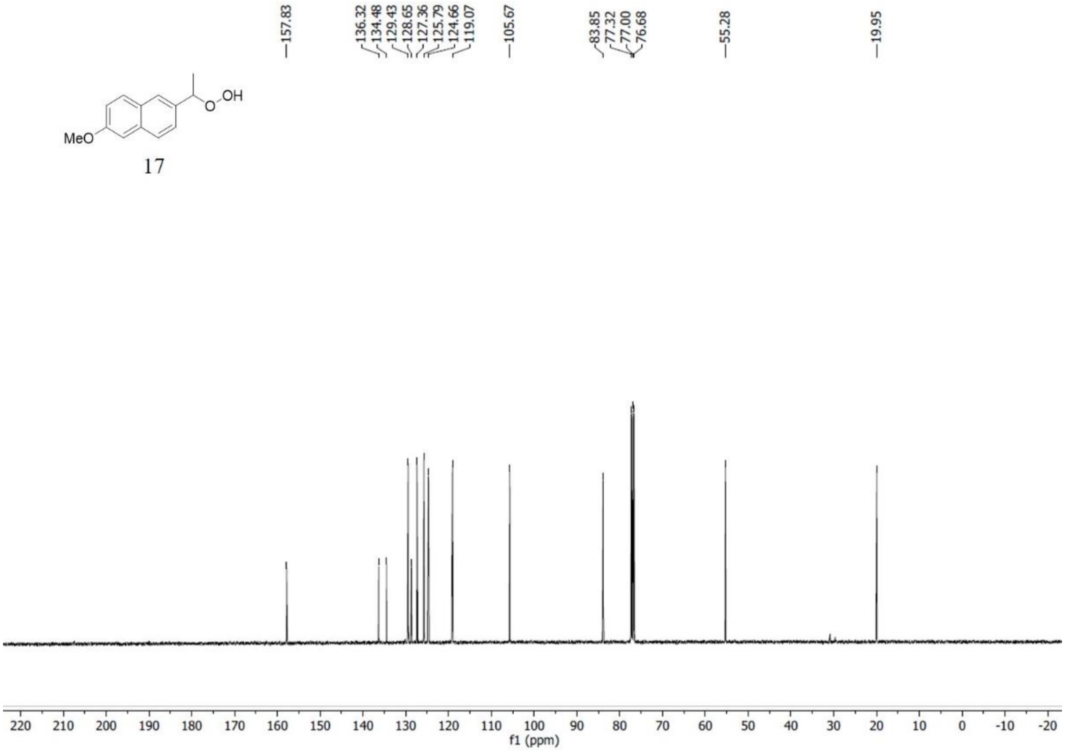

^{13}C NMR (101 MHz, $CDCl_3$)

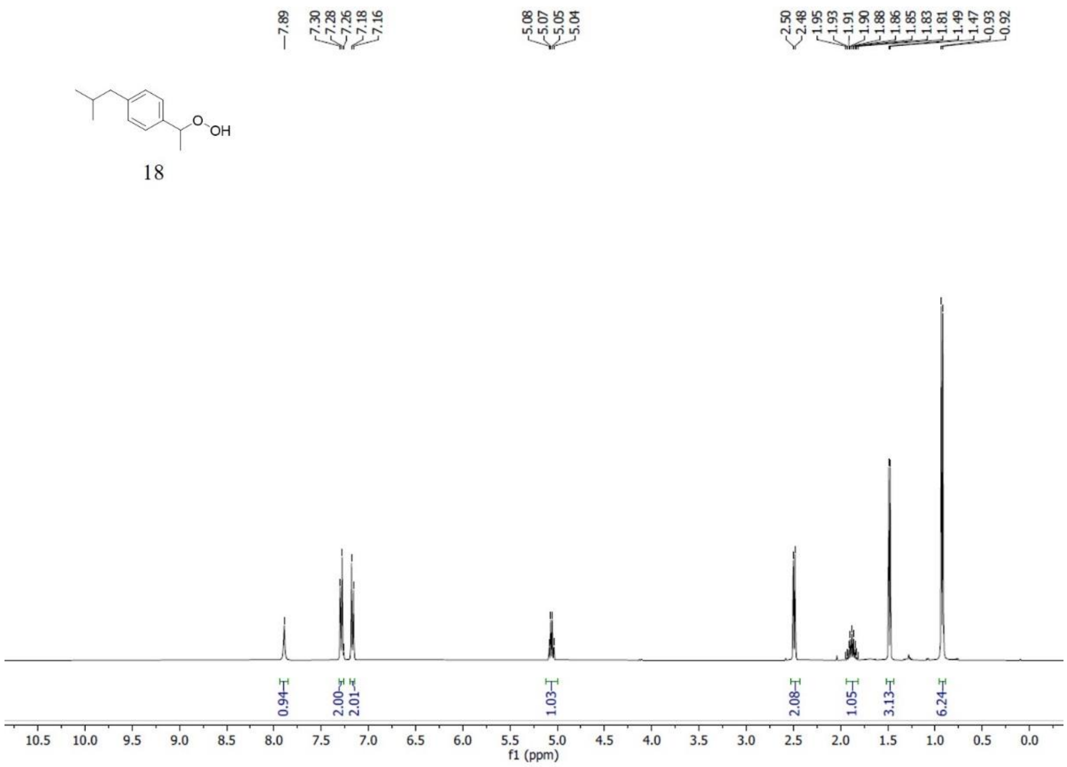

^{1}H NMR (400 MHz, $CDCl_3$)

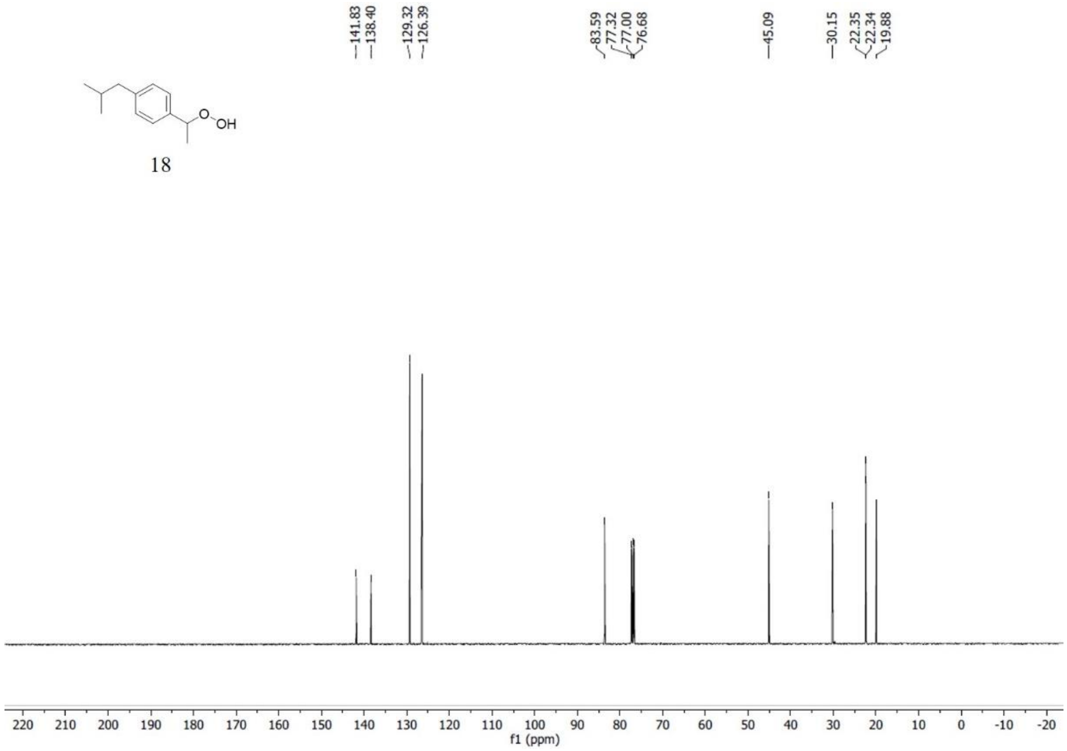

^{13}C NMR (101 MHz, $CDCl_3$)

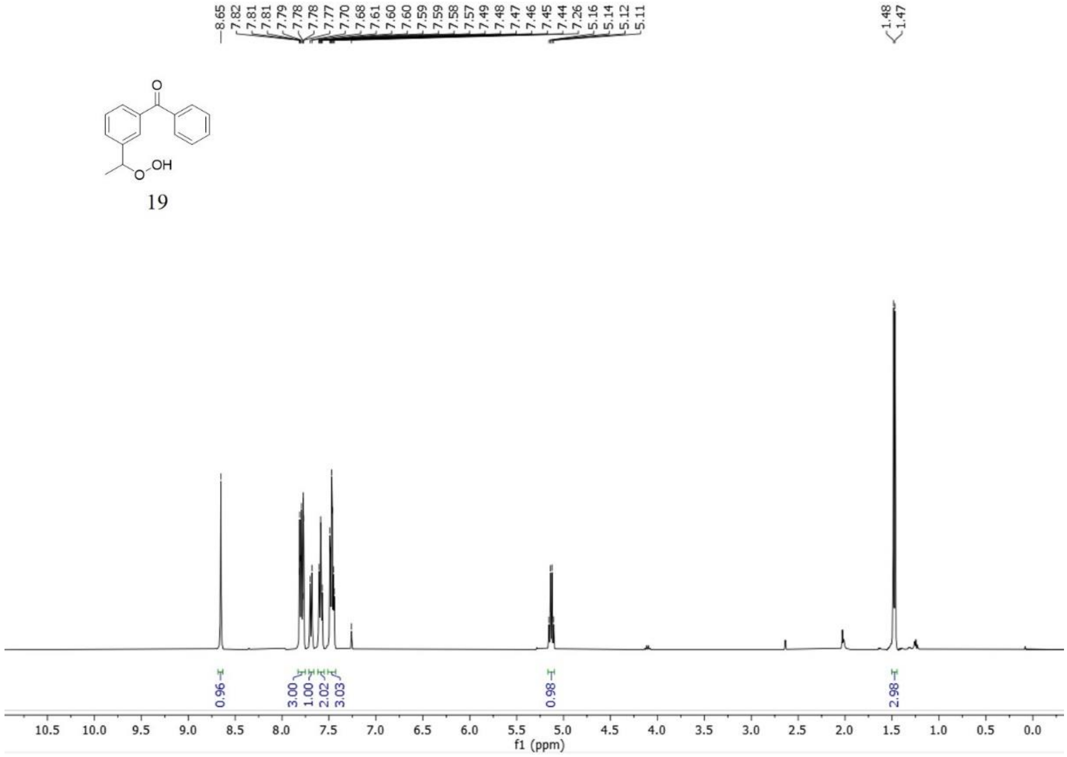

^{1}H NMR (400 MHz, $CDCl_3$)

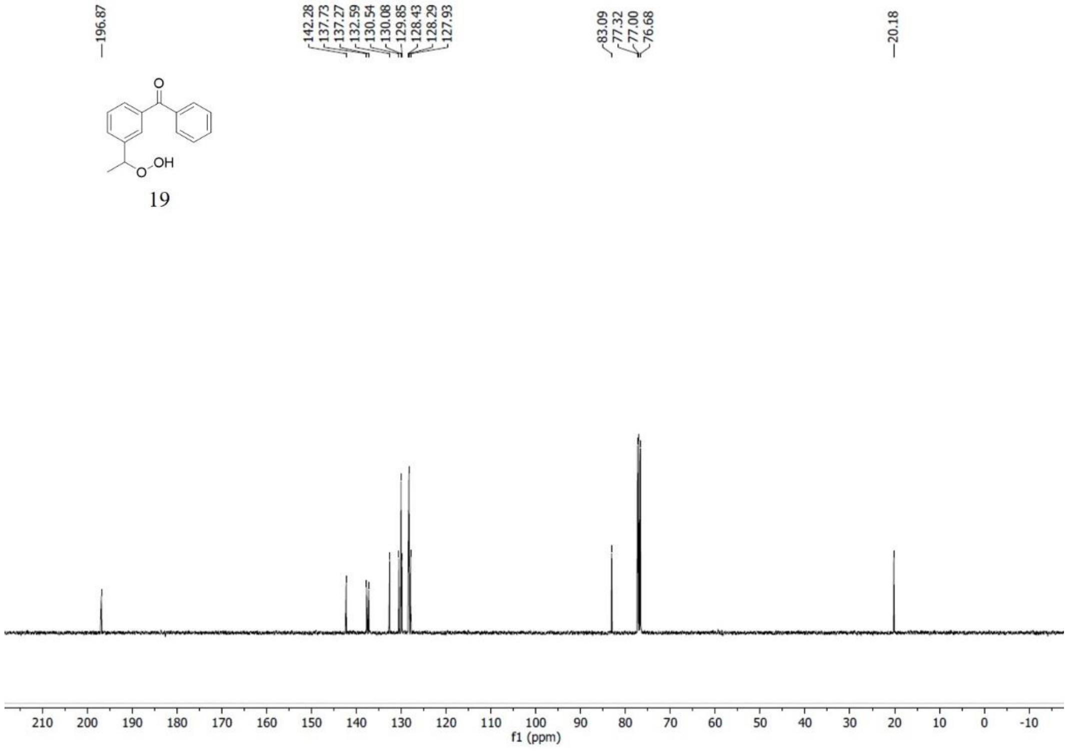

^{13}C NMR (101 MHz, $CDCl_3$)

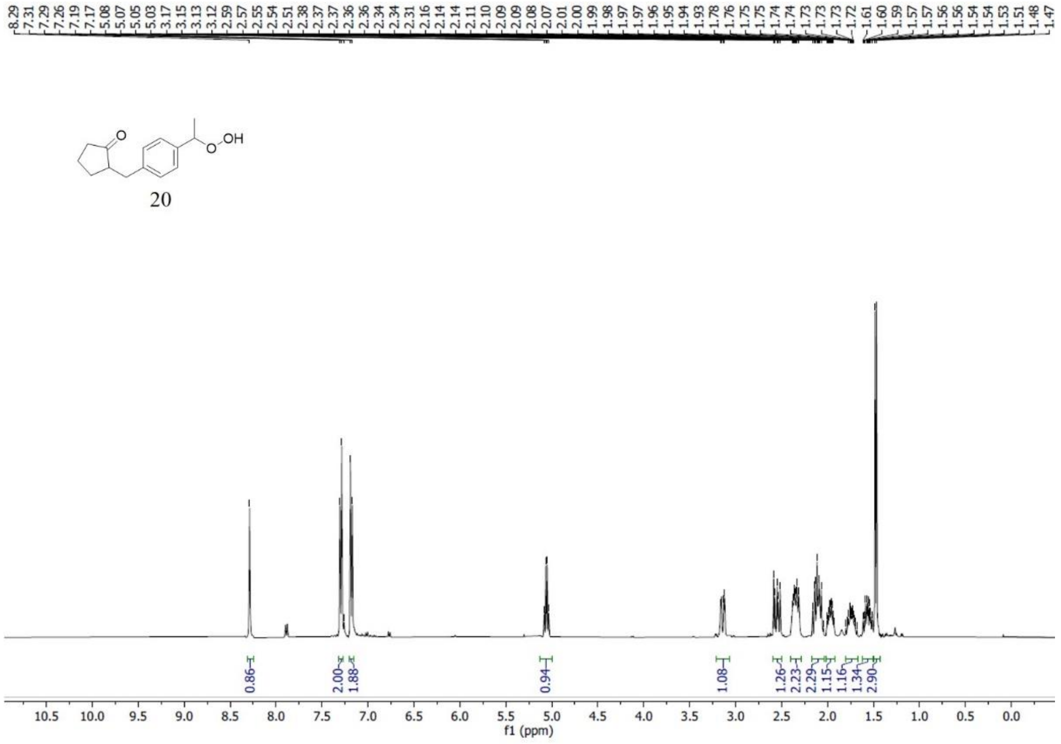

^{1}H NMR (400 MHz, $CDCl_3$)

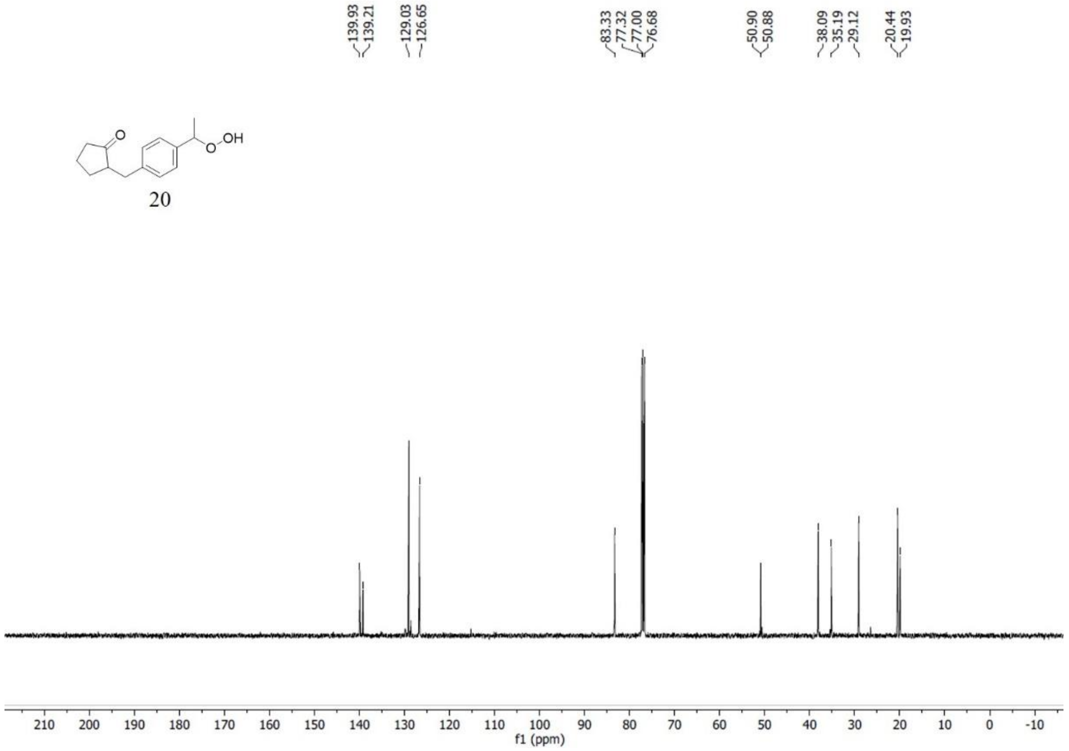

^{13}C NMR (101 MHz, $CDCl_3$)

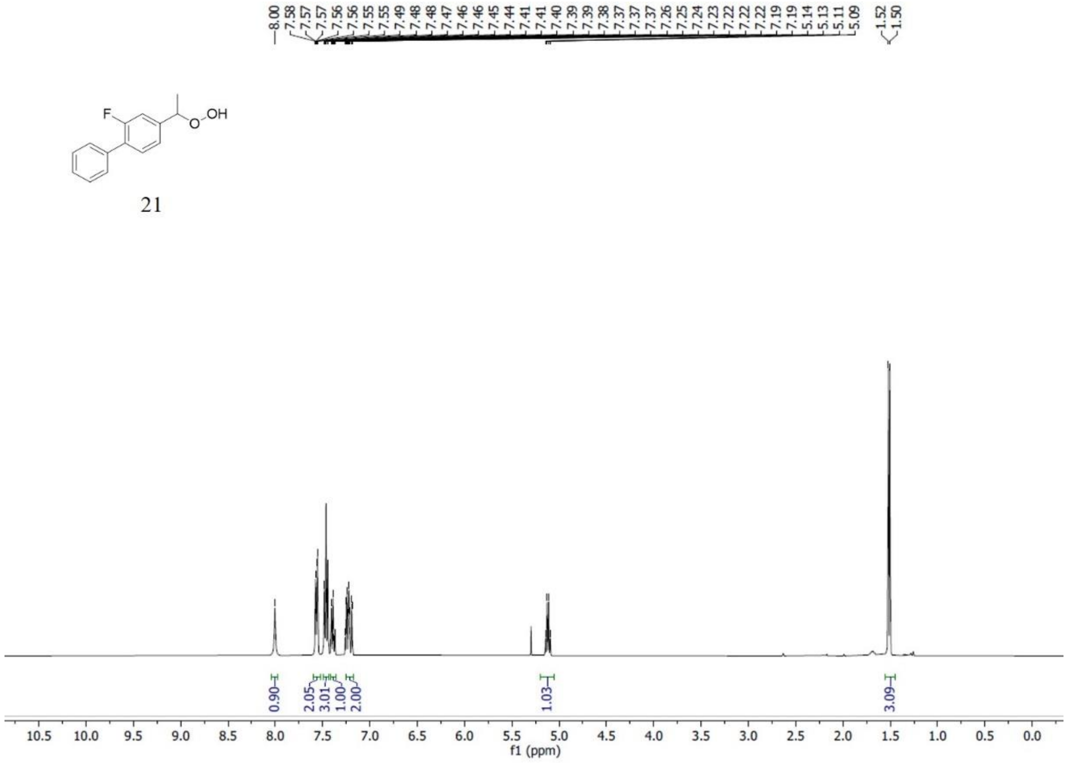

^{1}H NMR (400 MHz, $CDCl_3$)

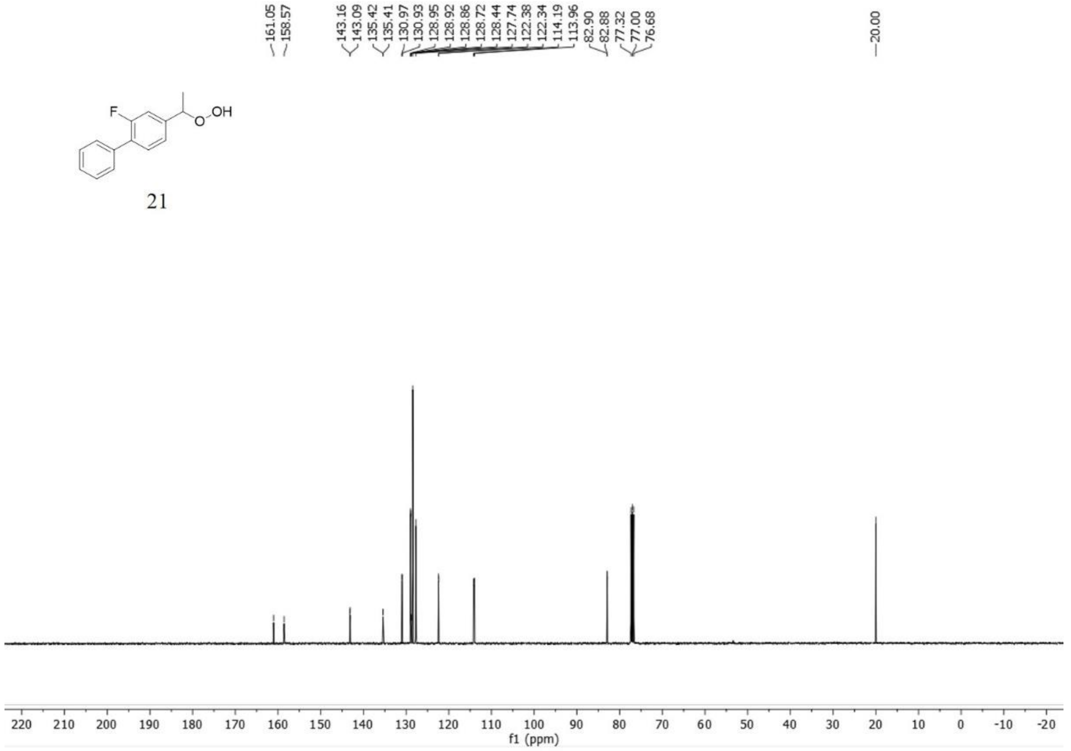

^{13}C NMR (101 MHz, $CDCl_3$)

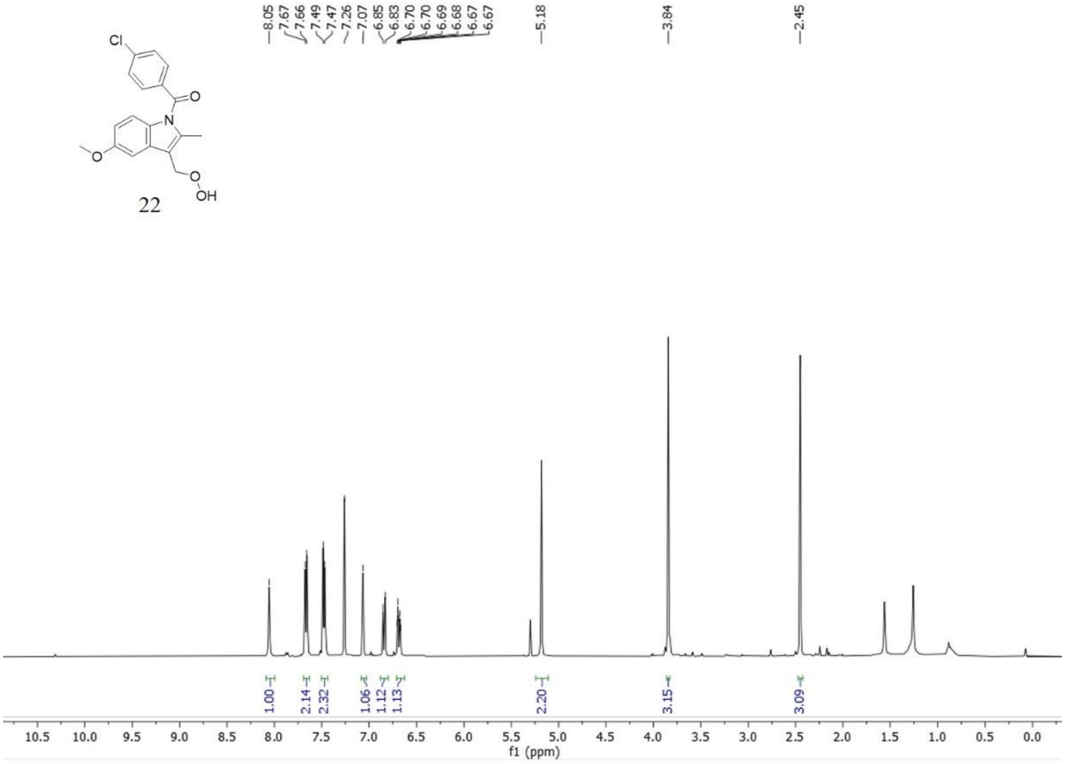

^{1}H NMR (400 MHz, $CDCl_3$)

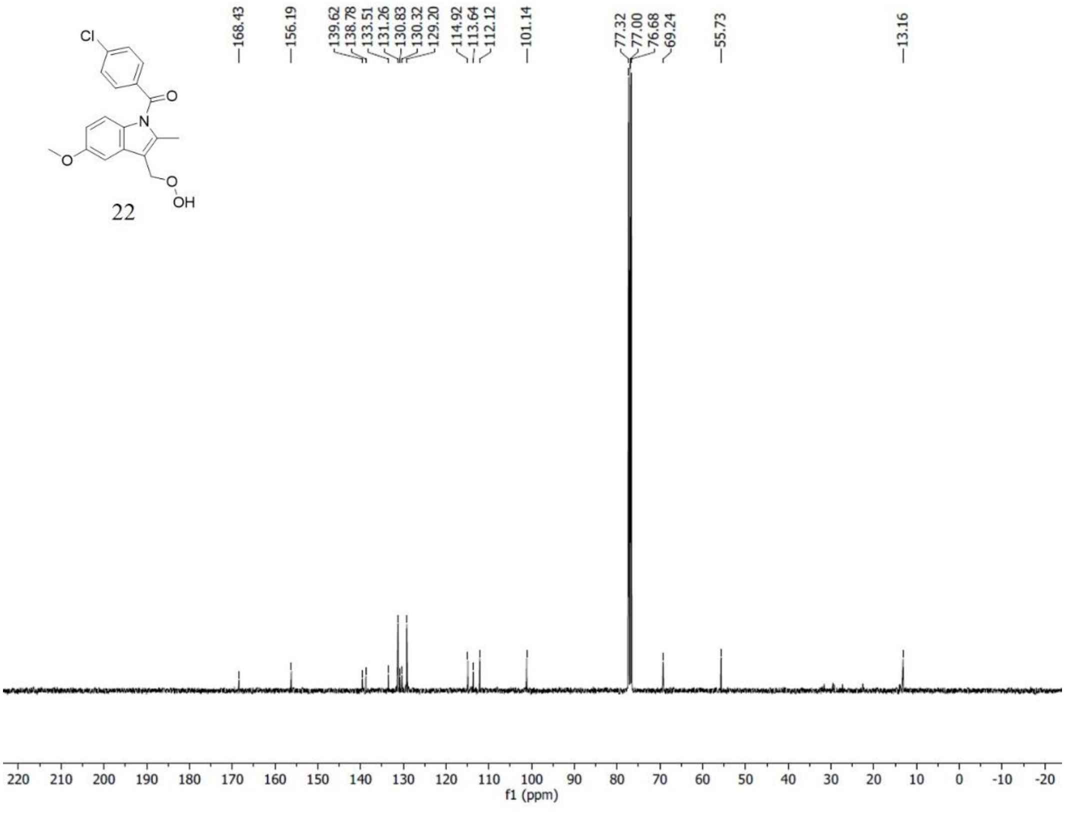

^{13}C NMR (101 MHz, $CDCl_3$)

8.2 NMR spectra of aldehydes and ketones

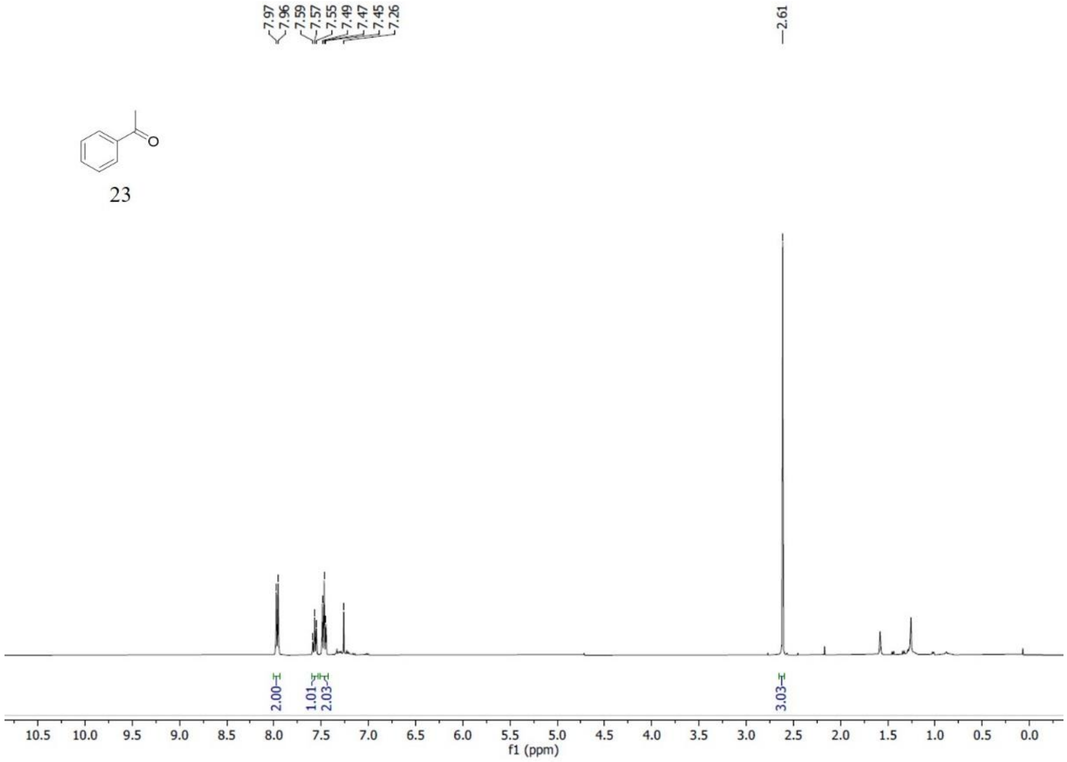

^{1}H NMR (400 MHz, $CDCl_3$)

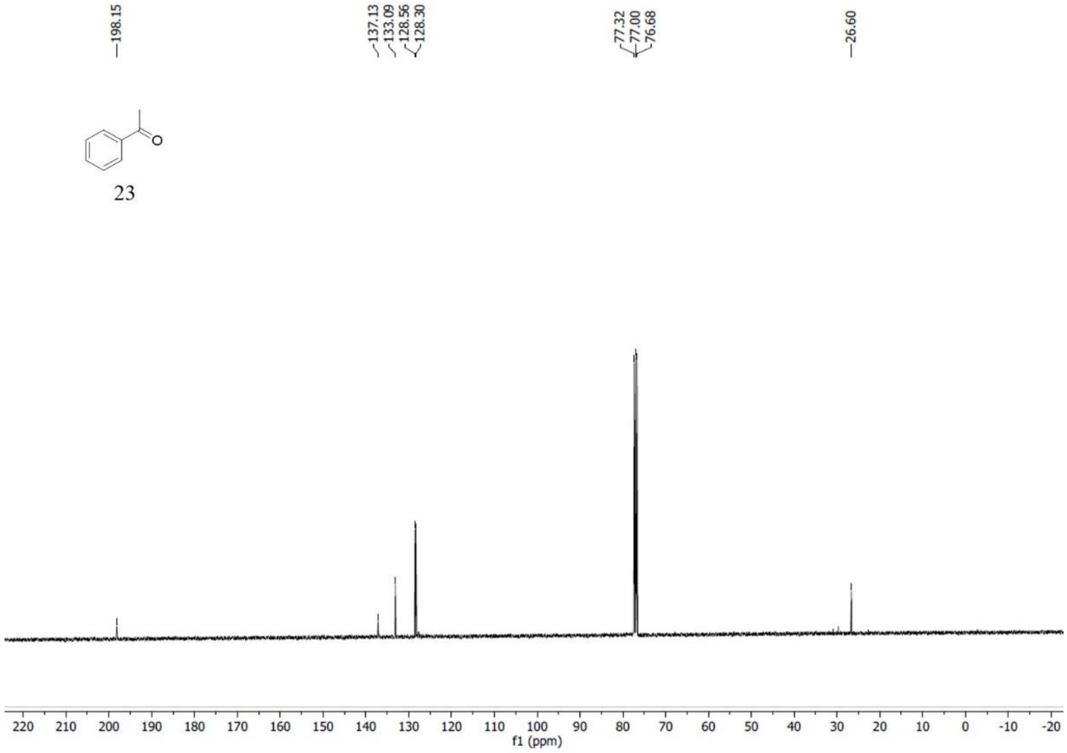

^{13}C NMR (101 MHz, $CDCl_3$)

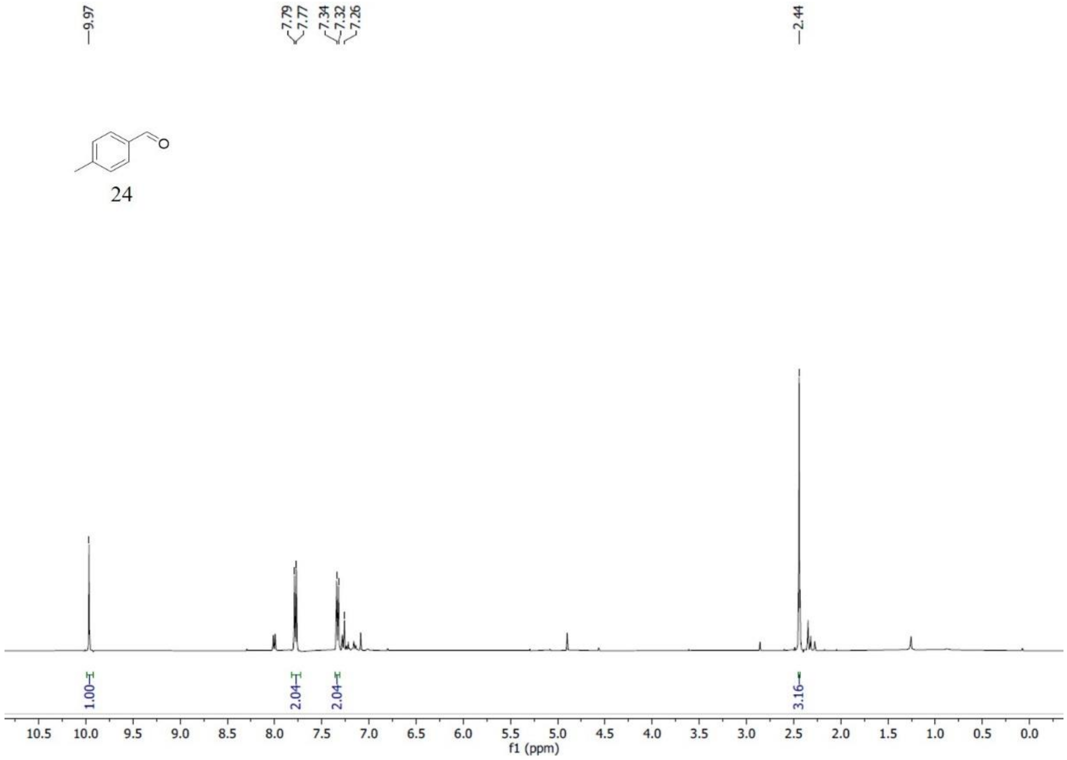

^{1}H NMR (400 MHz, $CDCl_3$)

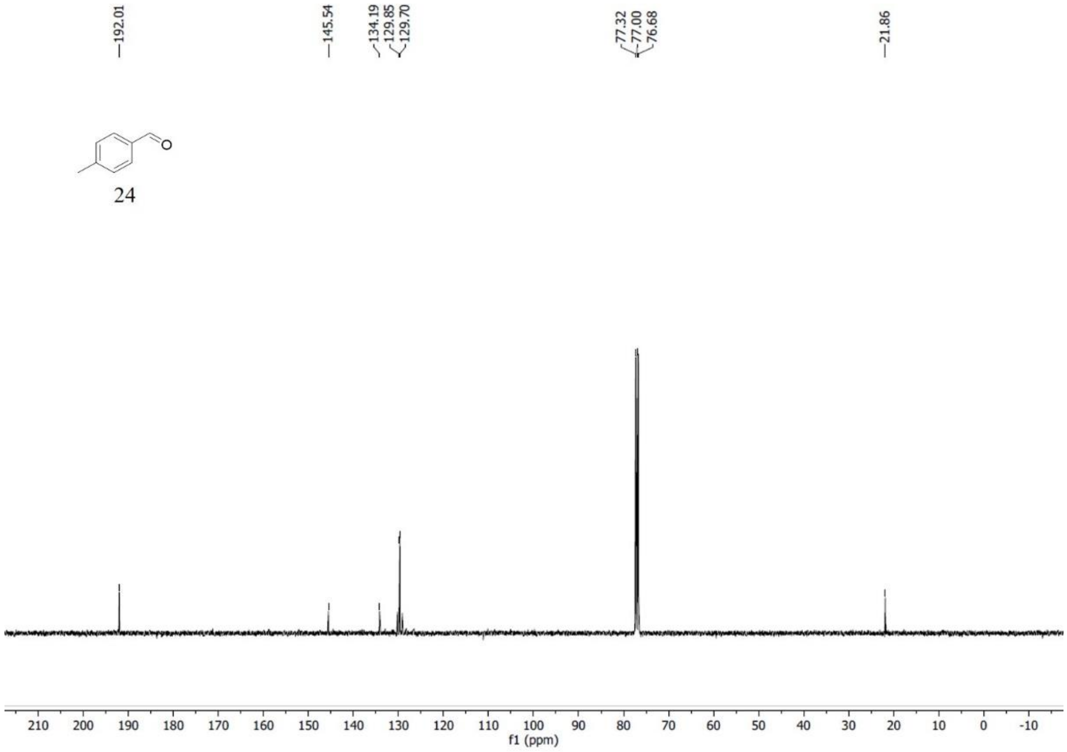

^{13}C NMR (101 MHz, $CDCl_3$)

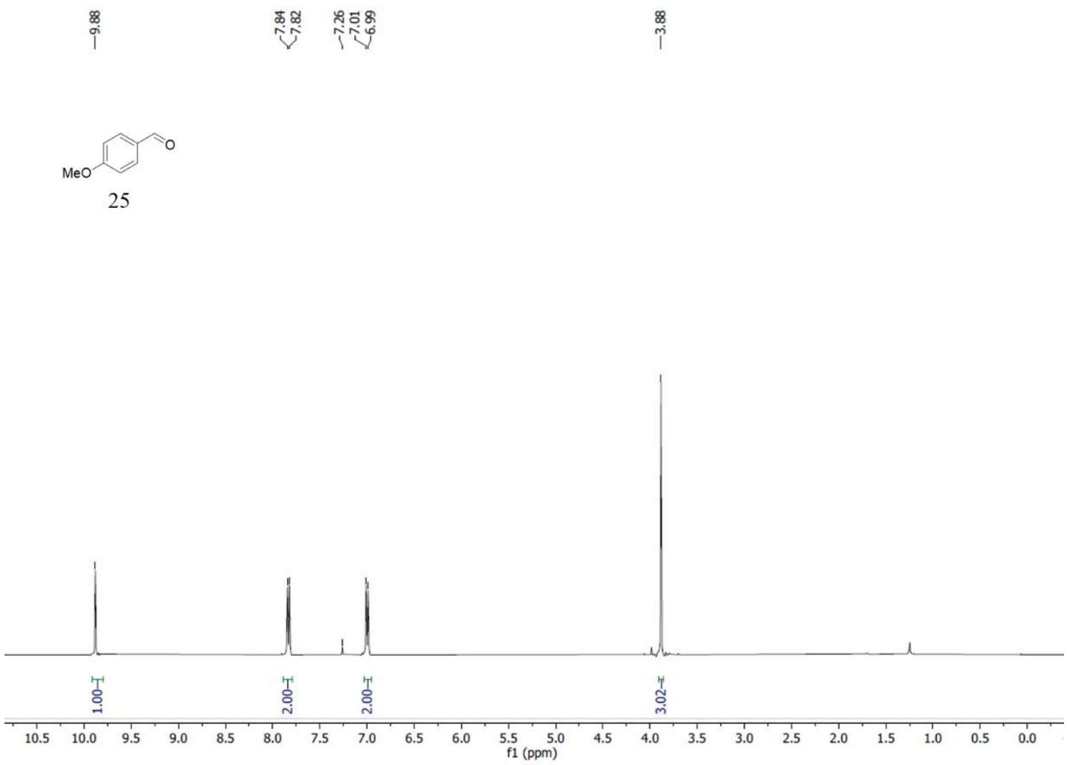

^{1}H NMR (400 MHz, $CDCl_3$)

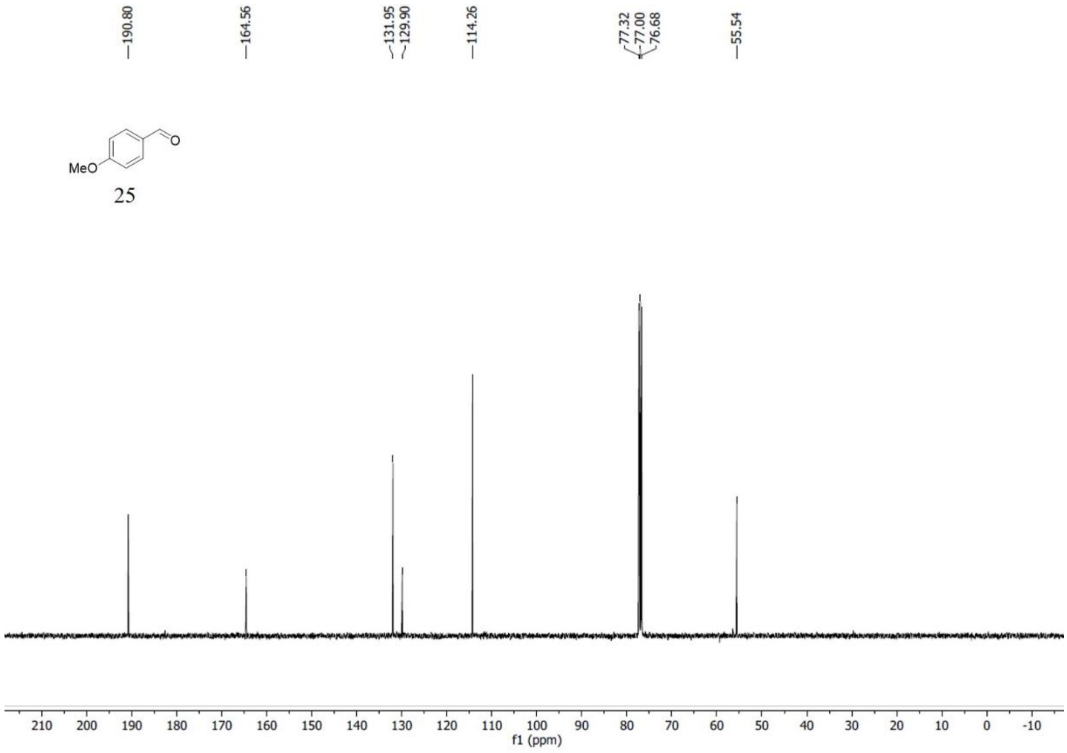

^{13}C NMR (101 MHz, $CDCl_3$)

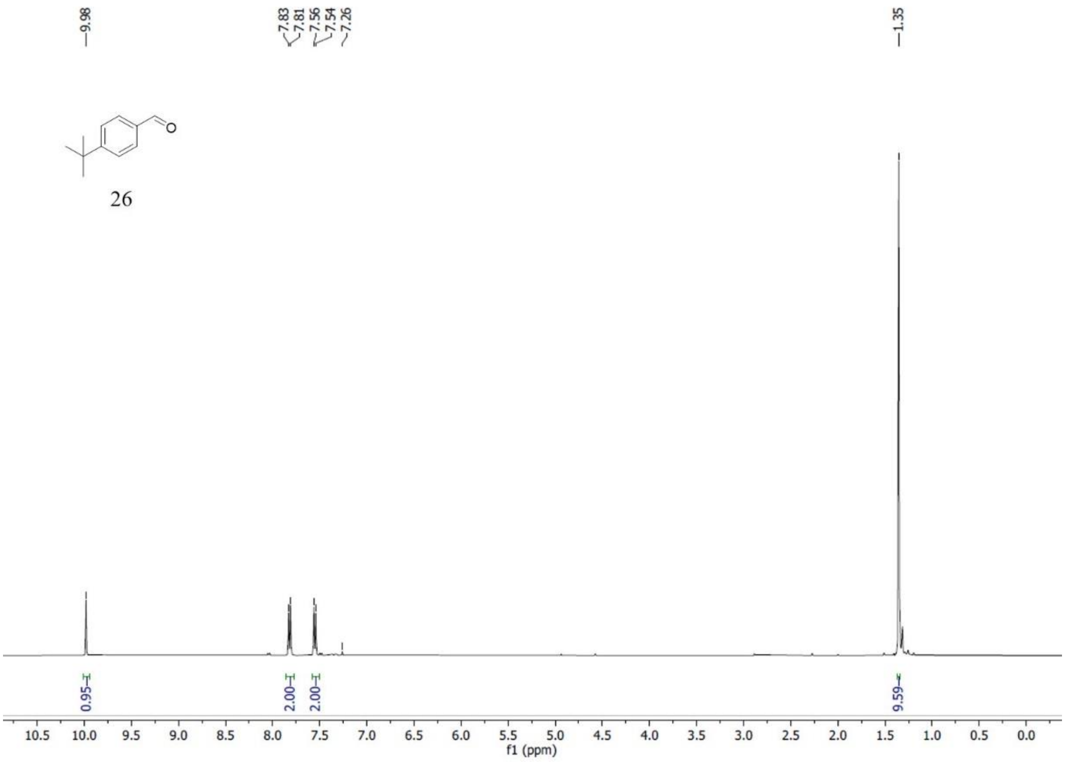

^1H NMR (400 MHz, $CDCl_3$)

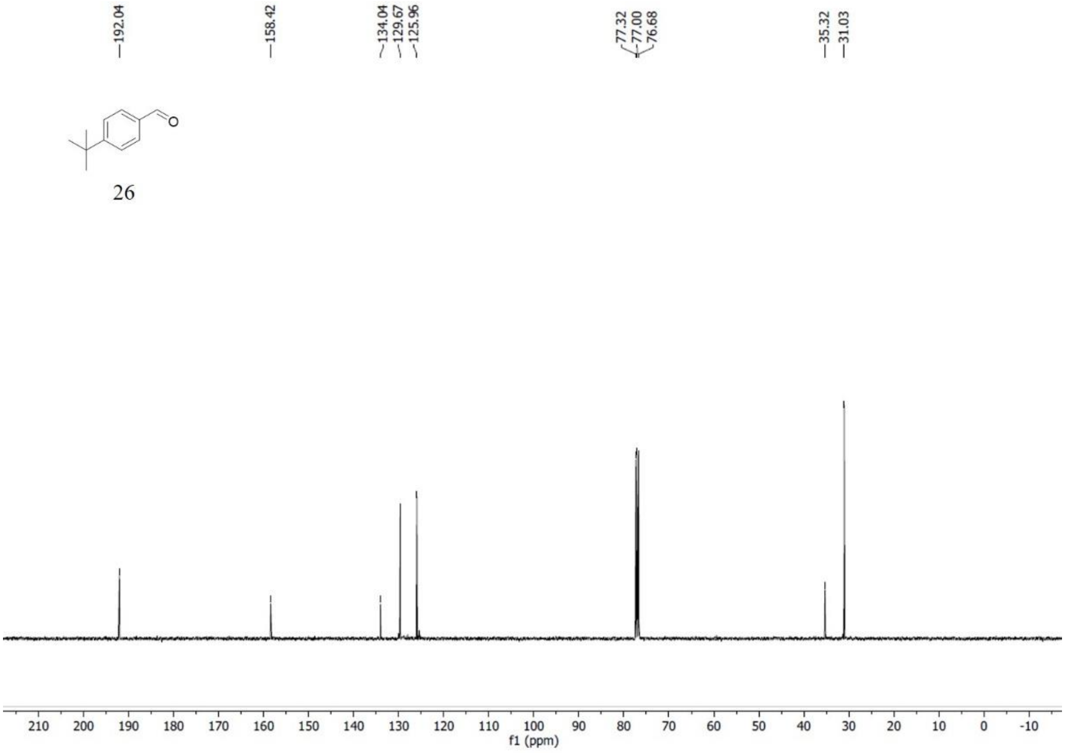

^{13}C NMR (101 MHz, $CDCl_3$)

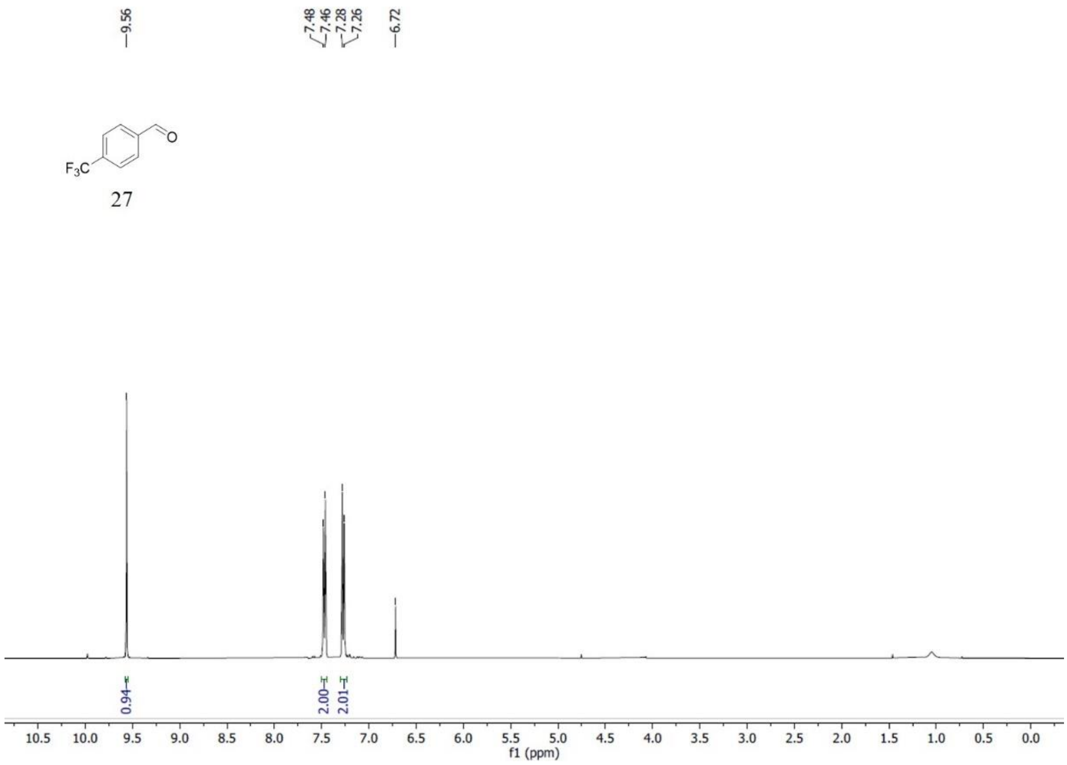

^{1}H NMR (400 MHz, $CDCl_3$)

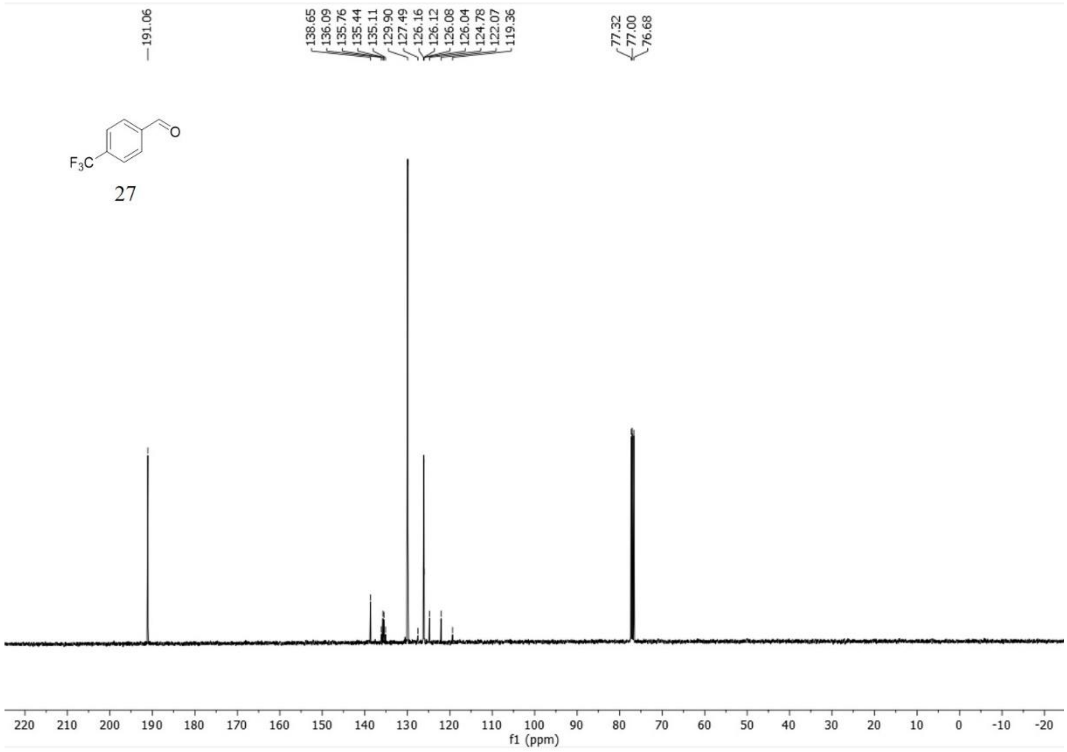

^{13}C NMR (101 MHz, $CDCl_3$)

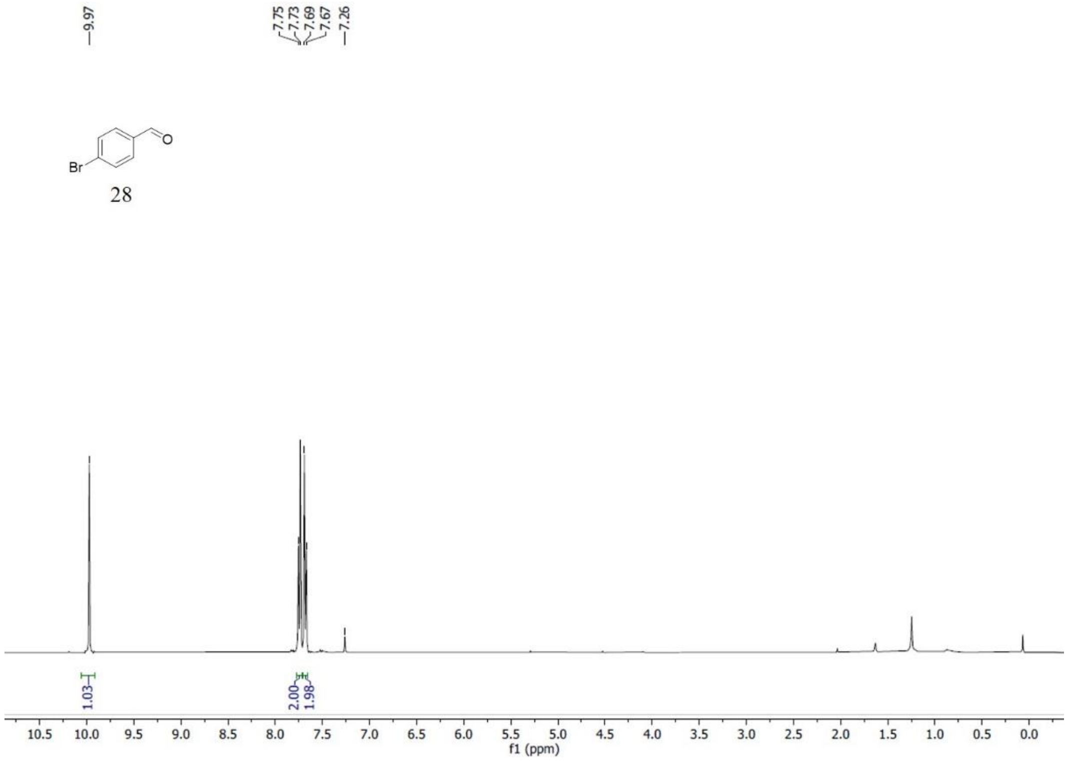

^{1}H NMR (400 MHz, $CDCl_3$)

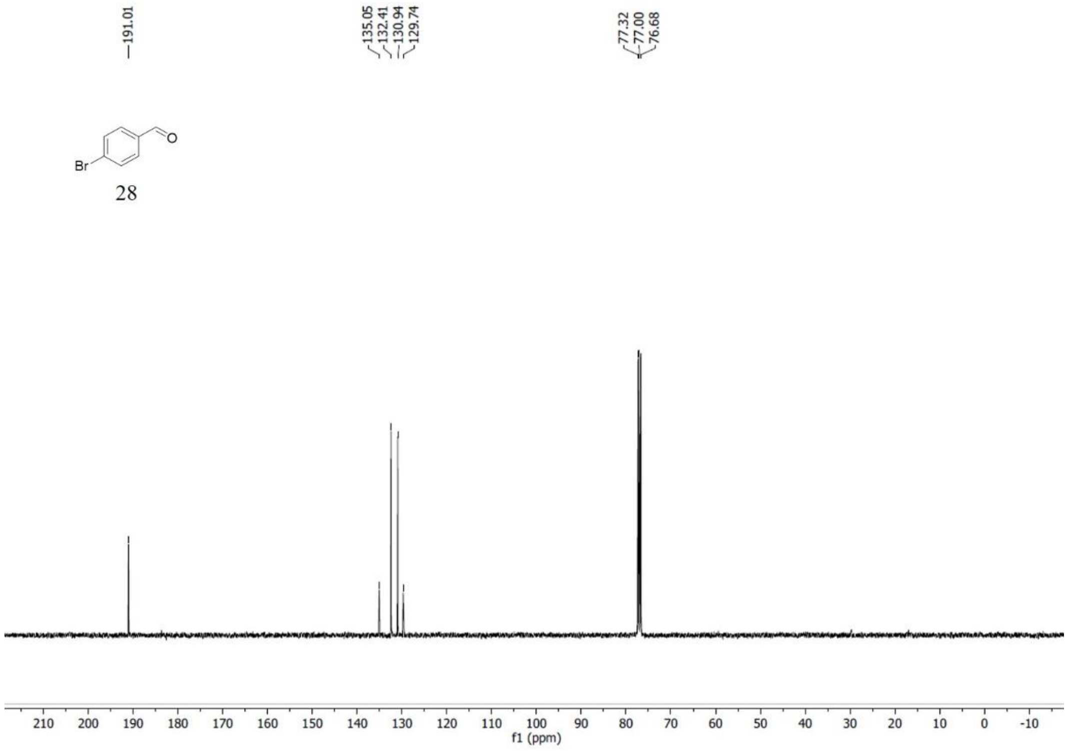

^{13}C NMR (101 MHz, $CDCl_3$)

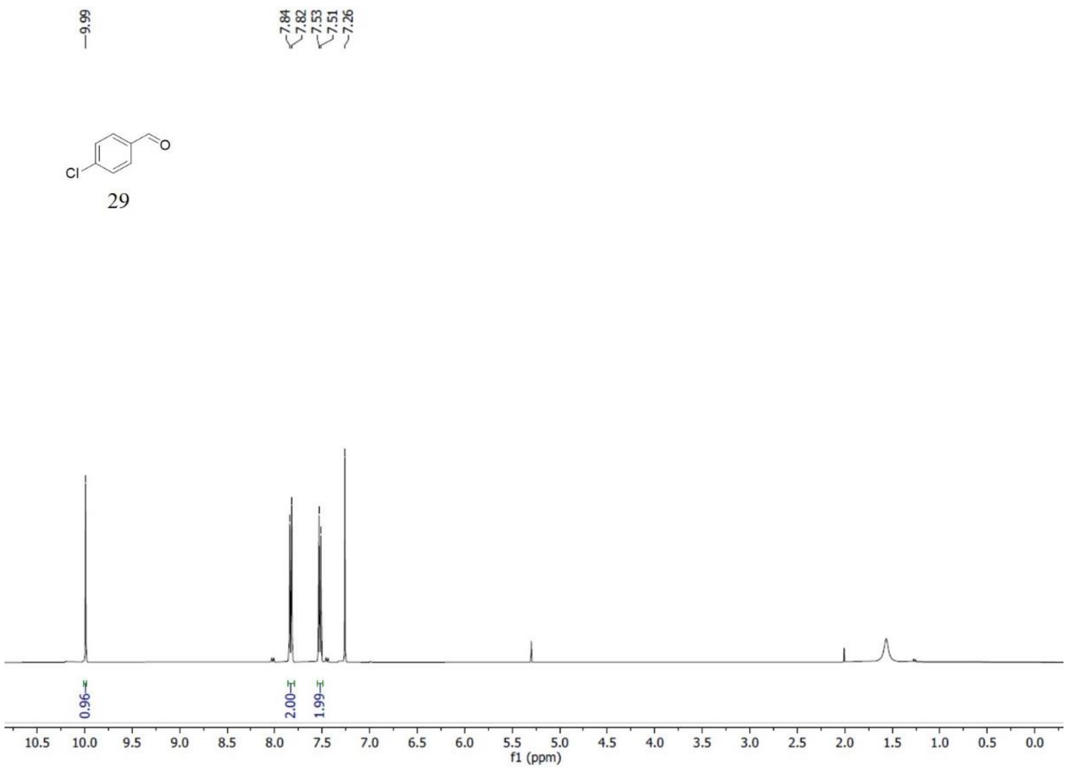

^{1}H NMR (400 MHz, $CDCl_3$)

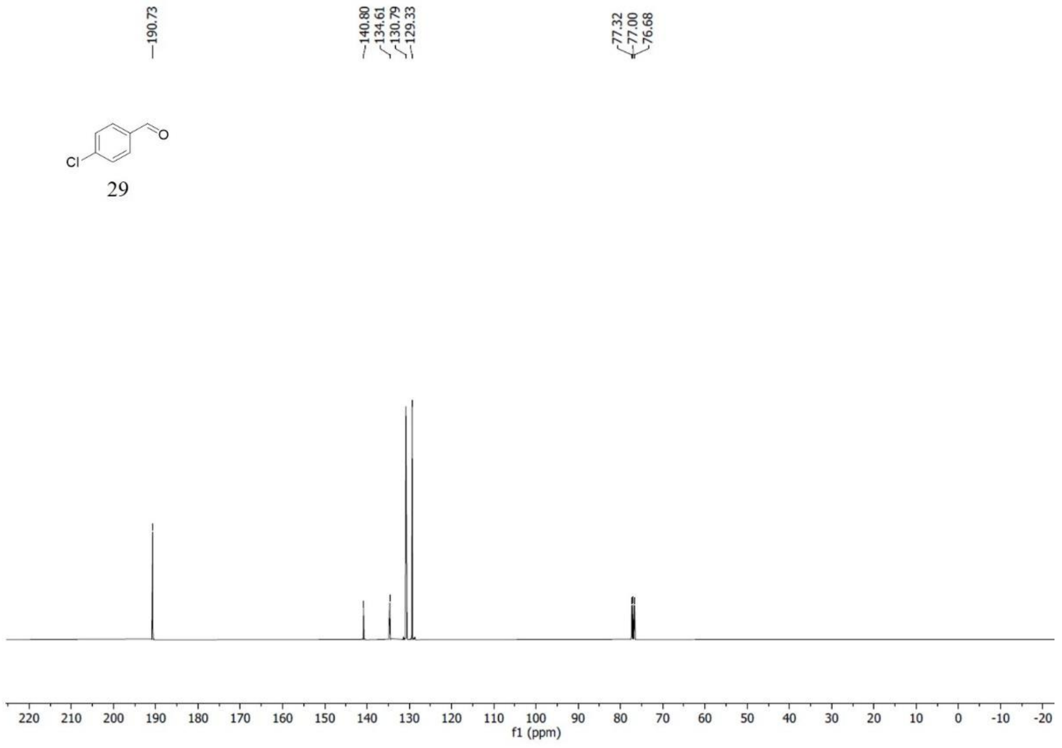

^{13}C NMR (101 MHz, $CDCl_3$)

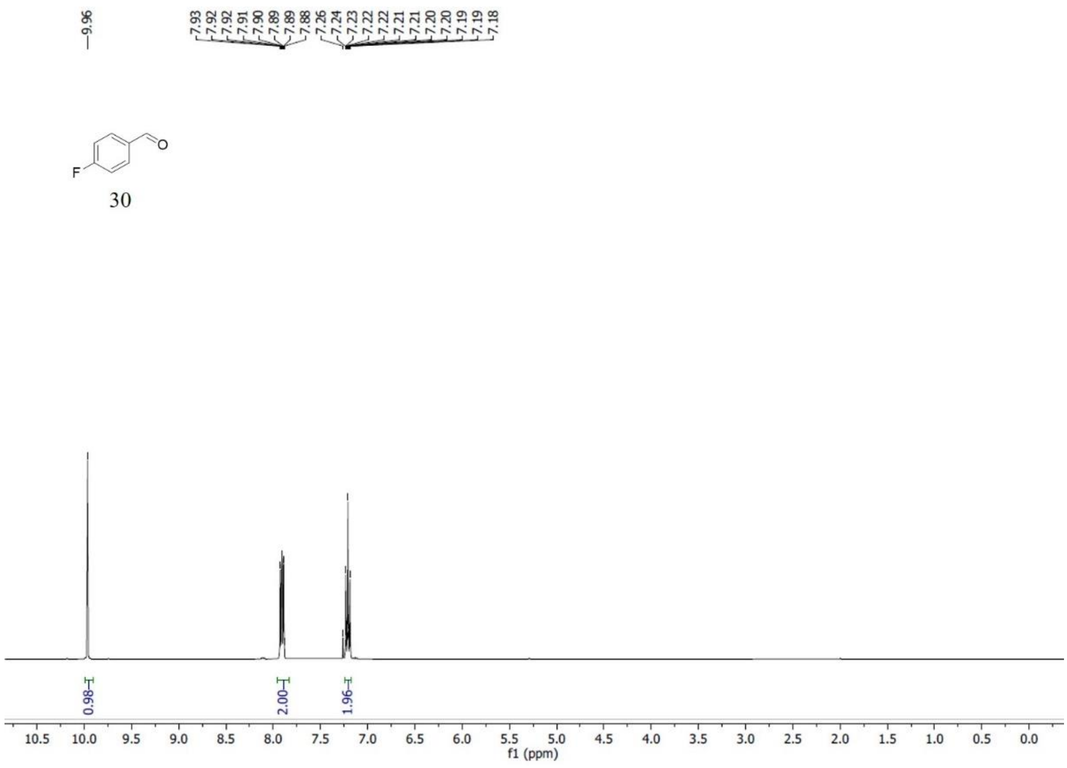

^{1}H NMR (400 MHz, $CDCl_3$)

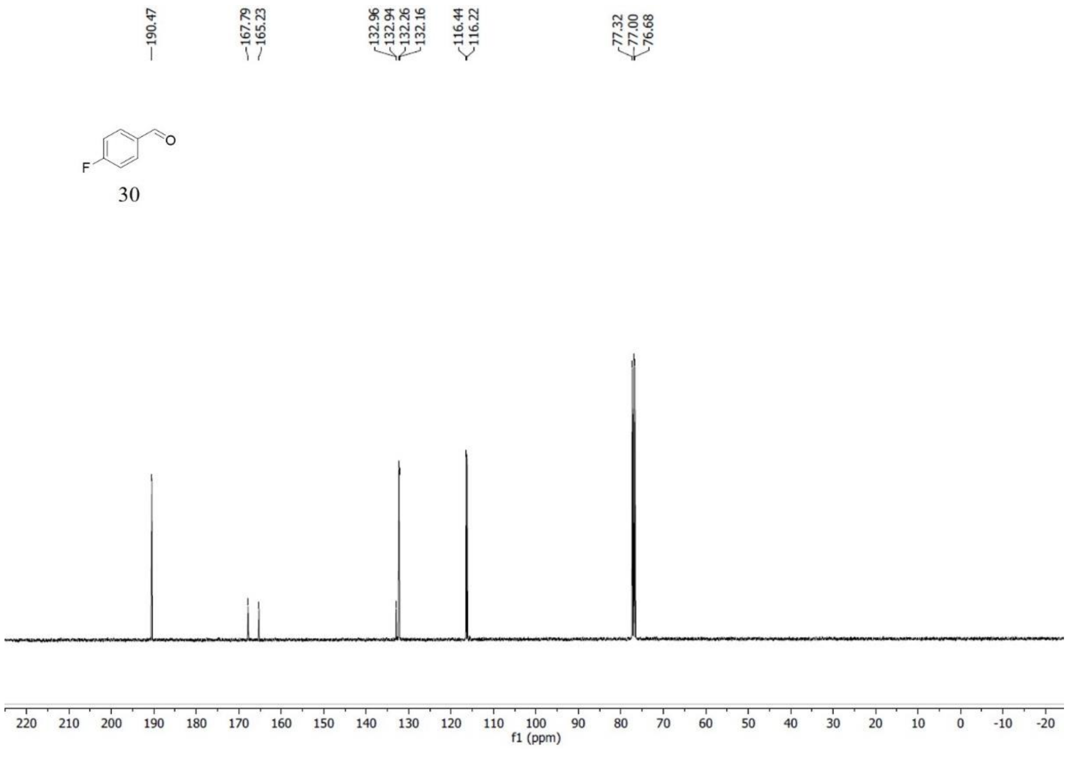

^{13}C NMR (101 MHz, $CDCl_3$)

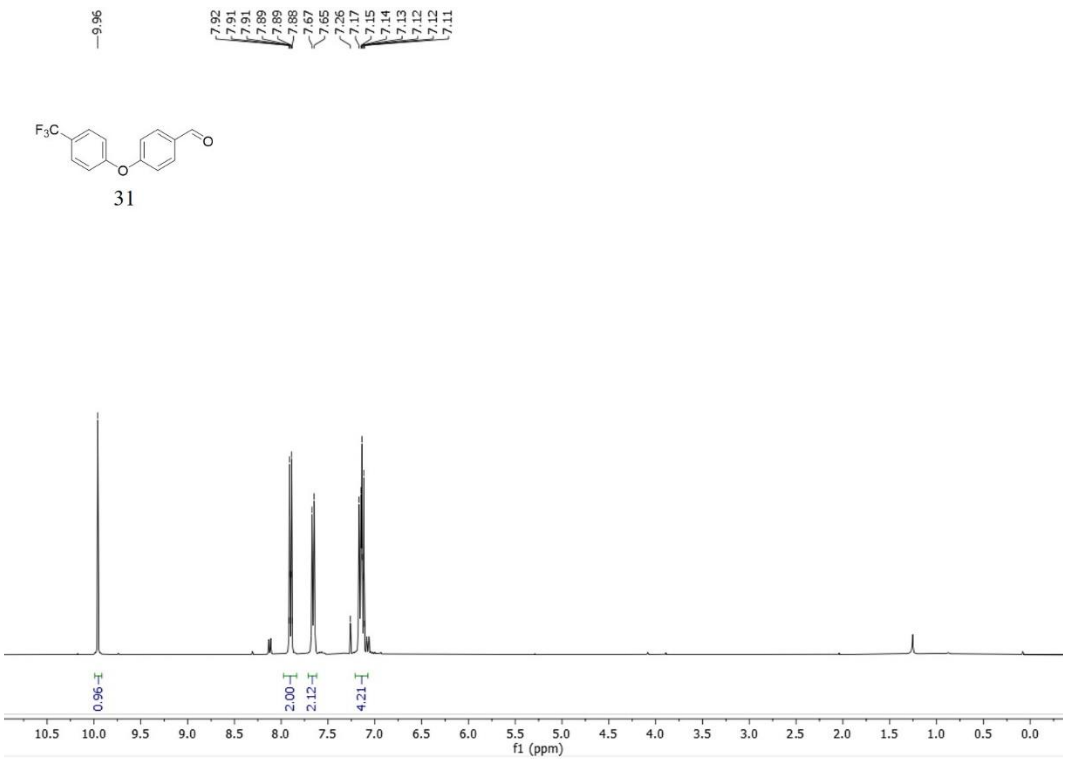

^{1}H NMR (400 MHz, $CDCl_3$)

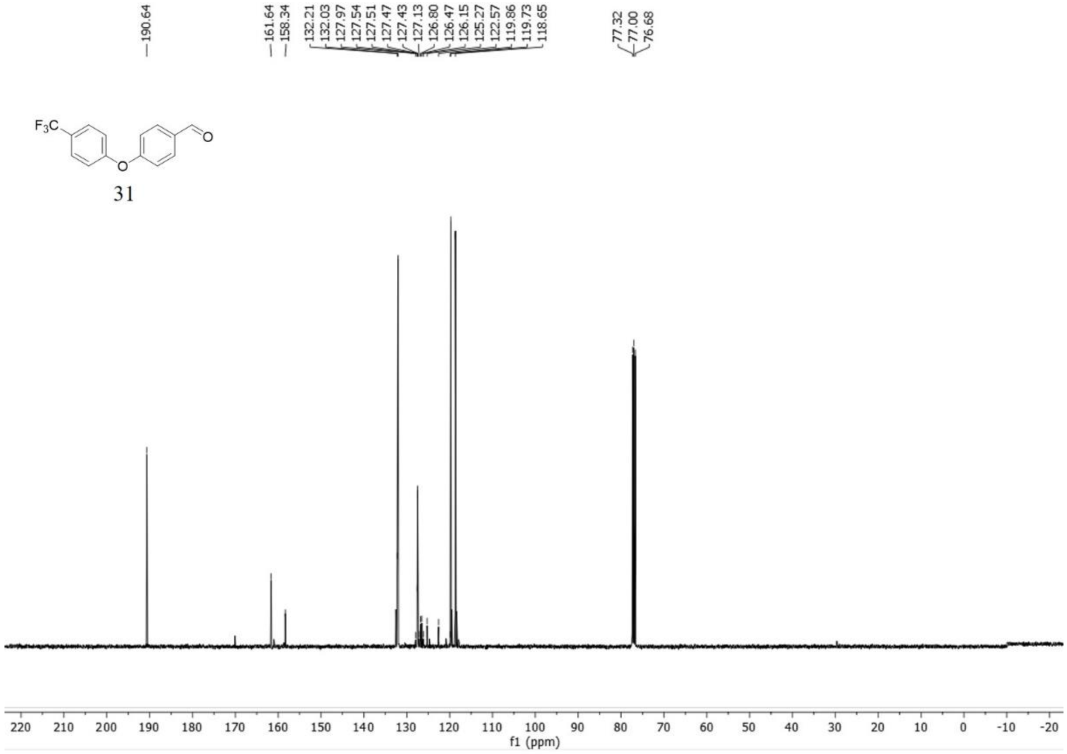

^{13}C NMR (101 MHz, $CDCl_3$)

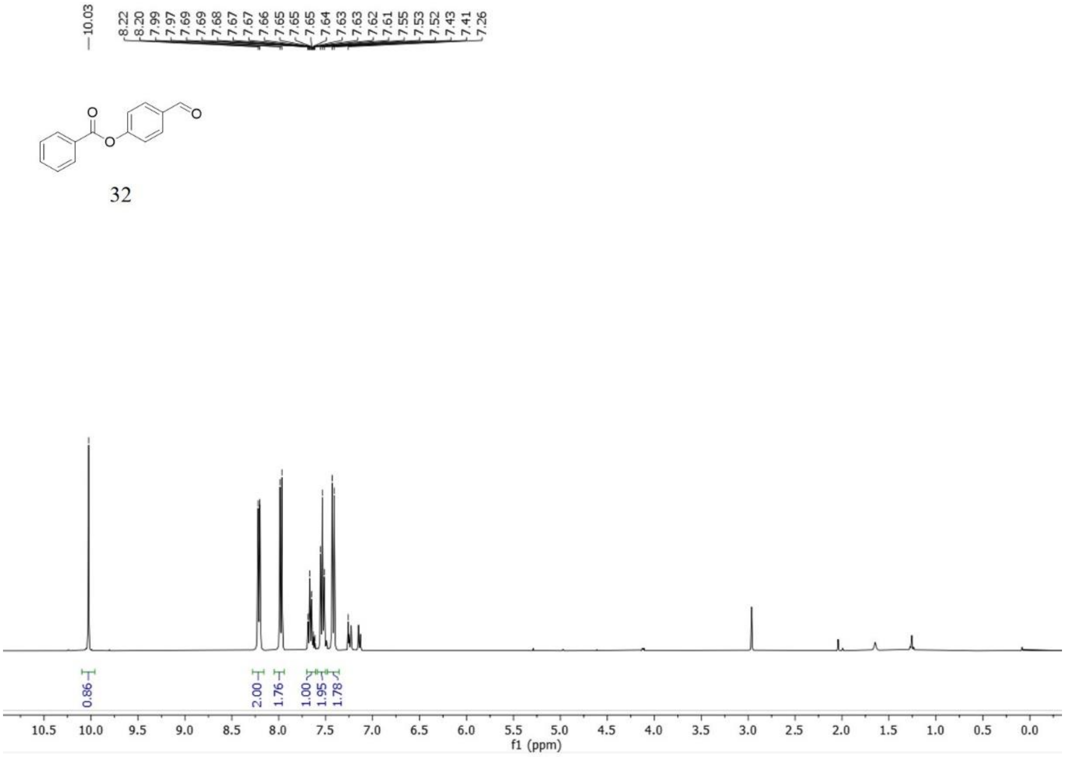

^{1}H NMR (400 MHz, $CDCl_3$)

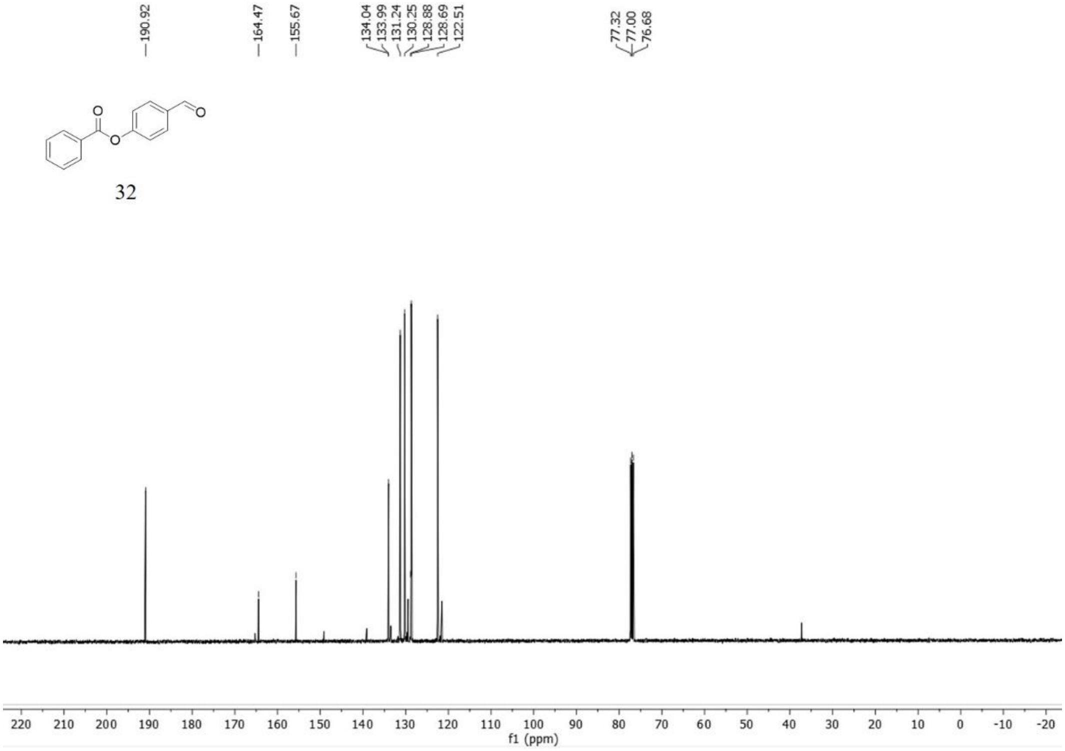

^{13}C NMR (101 MHz, $CDCl_3$)

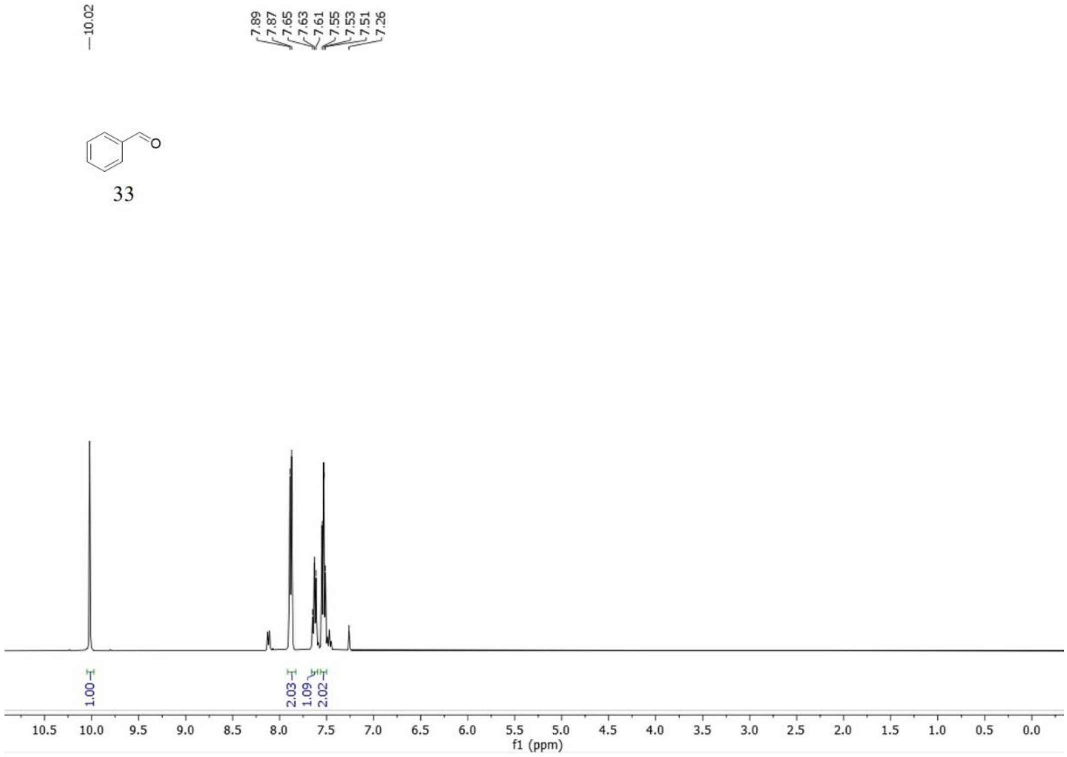

^1H NMR (400 MHz, $CDCl_3$)

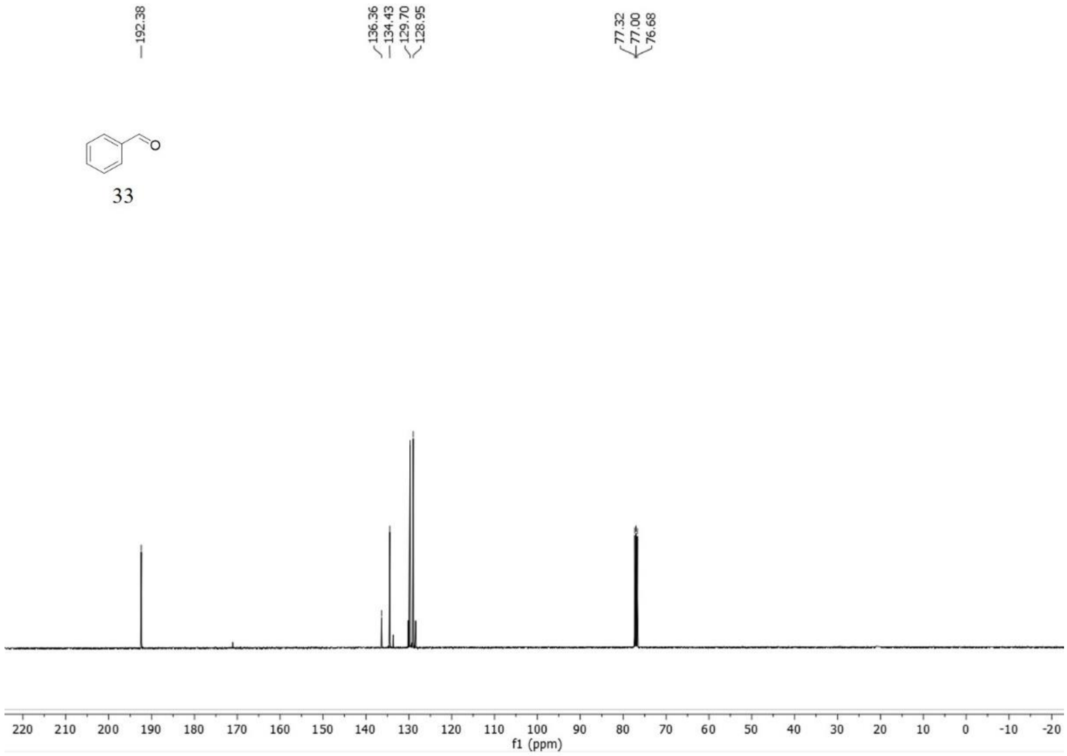

^{13}C NMR (101 MHz, $CDCl_3$)

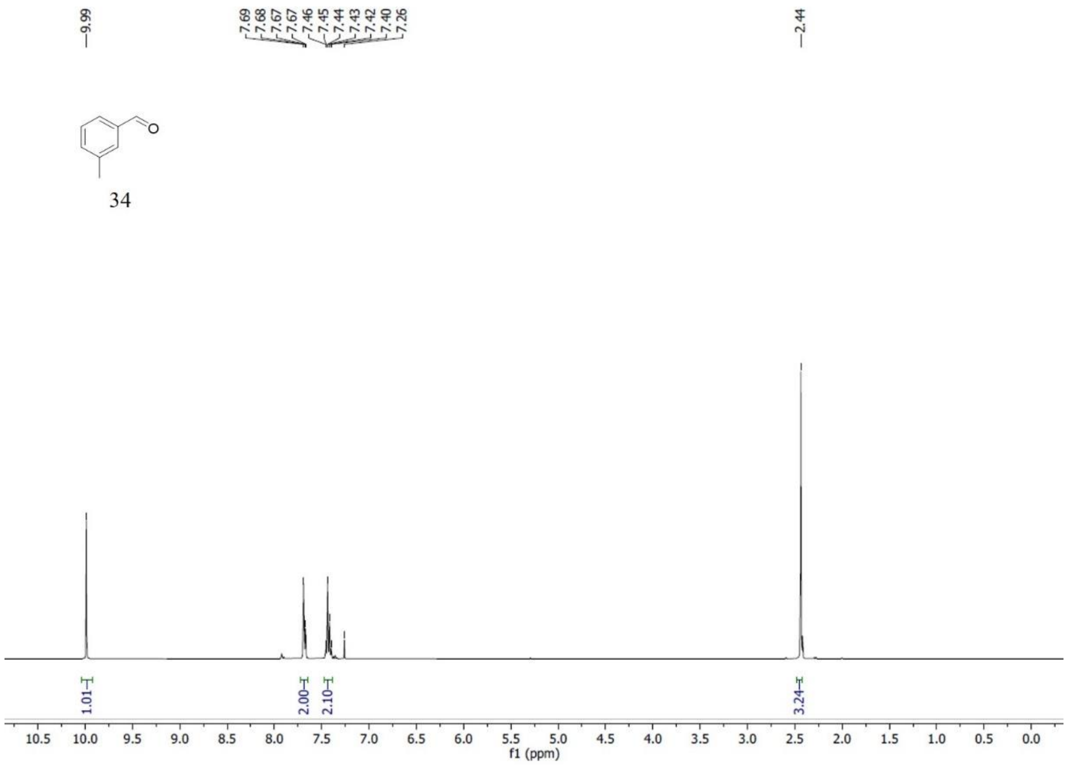

^{1}H NMR (400 MHz, $CDCl_3$)

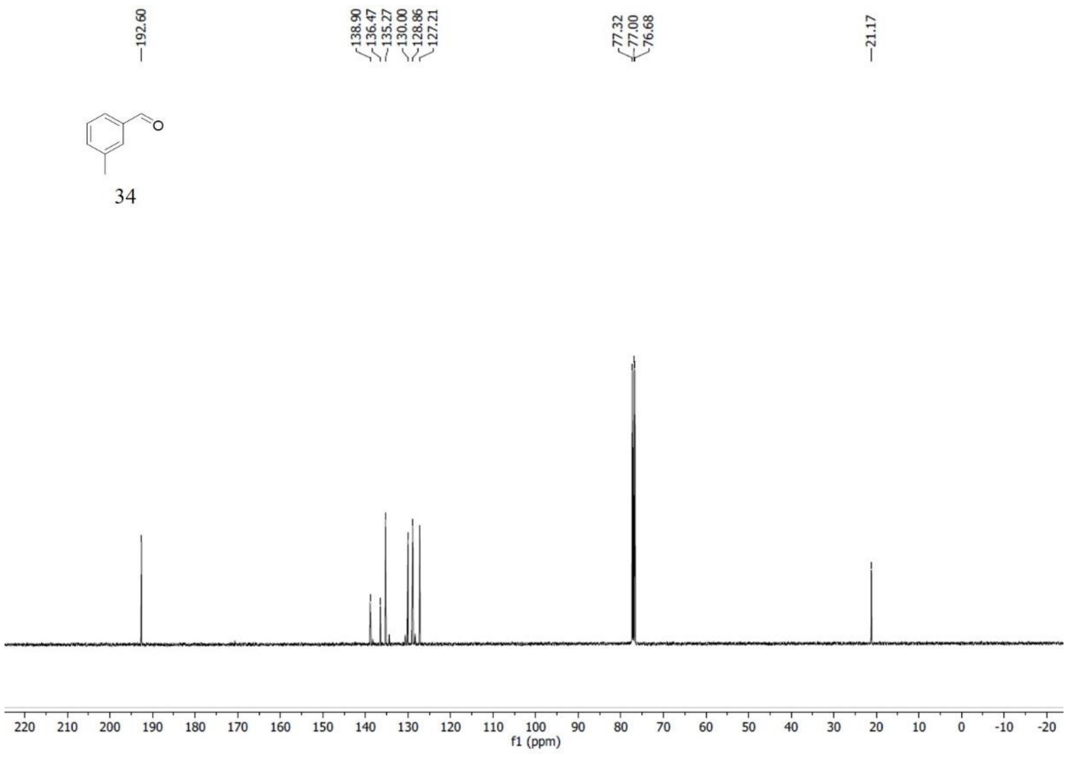

^{13}C NMR (101 MHz, $CDCl_3$)

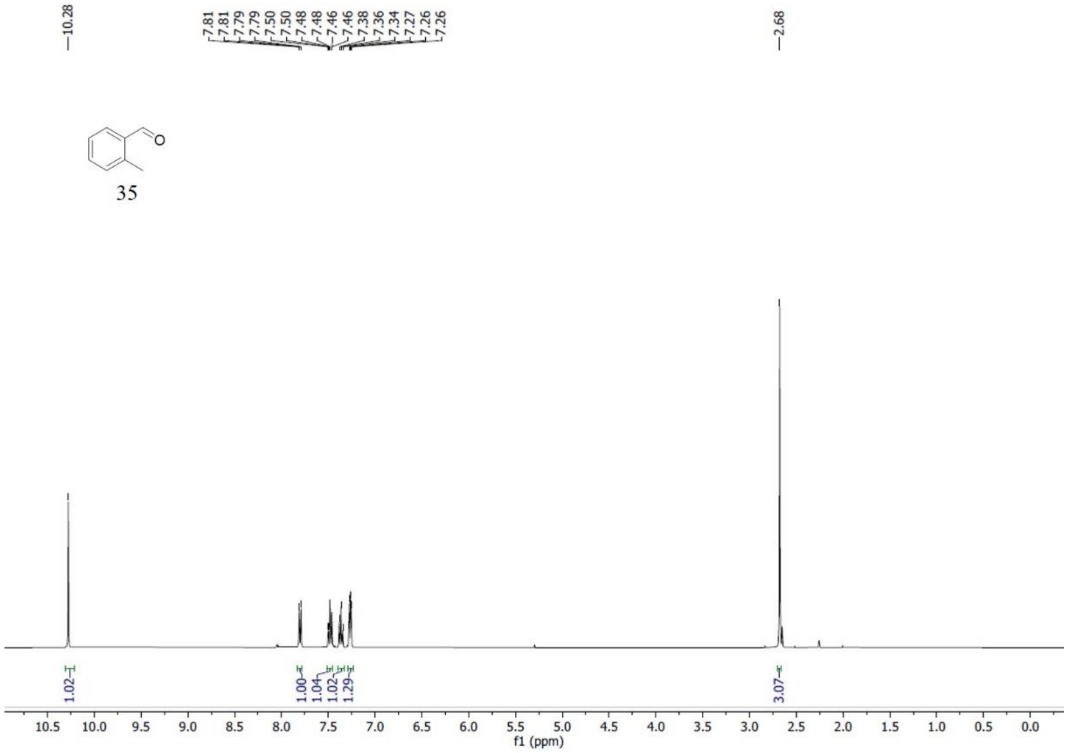

^{1}H NMR (400 MHz, $CDCl_3$)

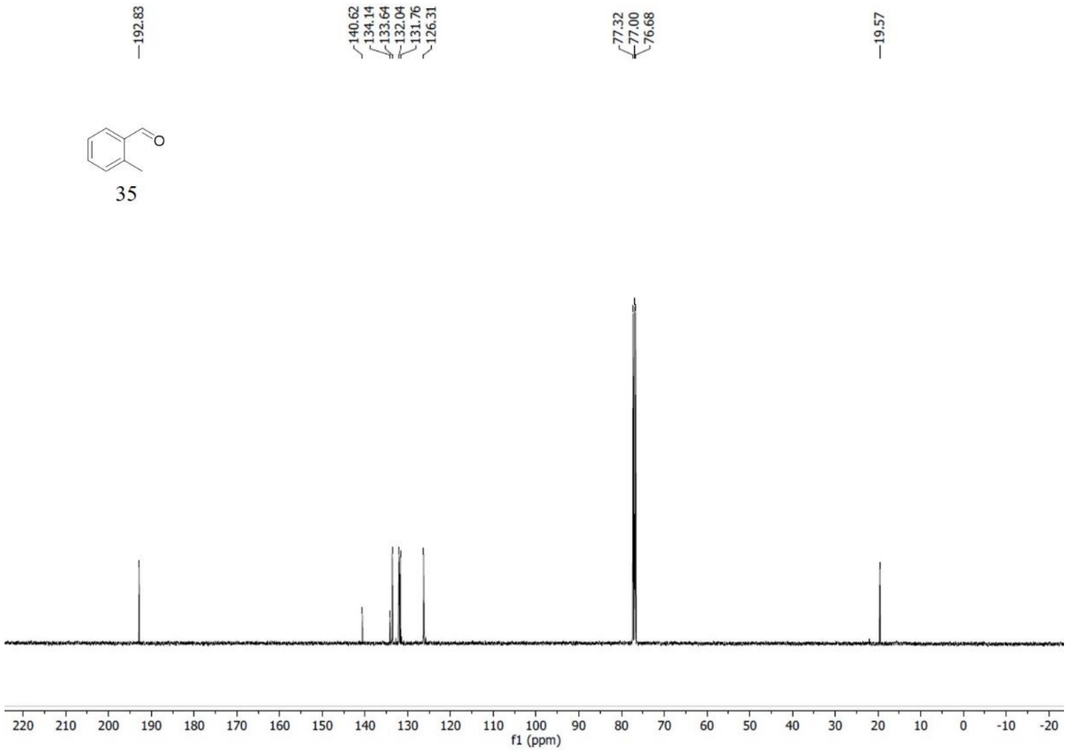

^{13}C NMR (101 MHz, $CDCl_3$)

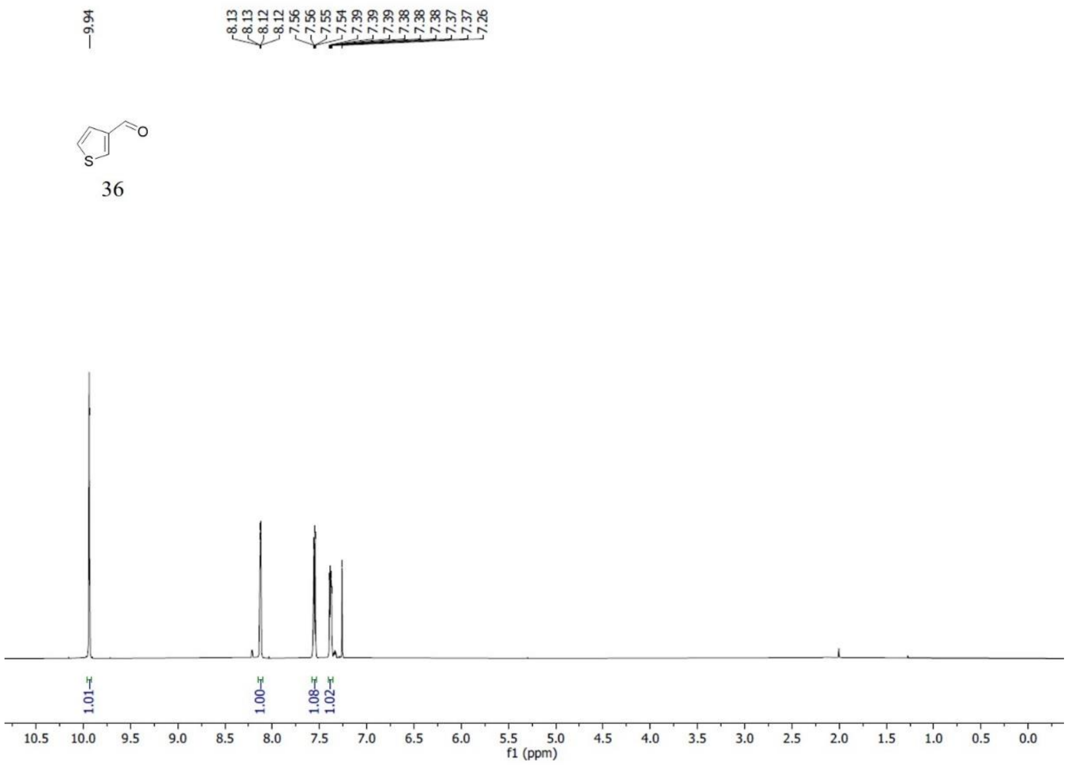

^{1}H NMR (400 MHz, $CDCl_3$)

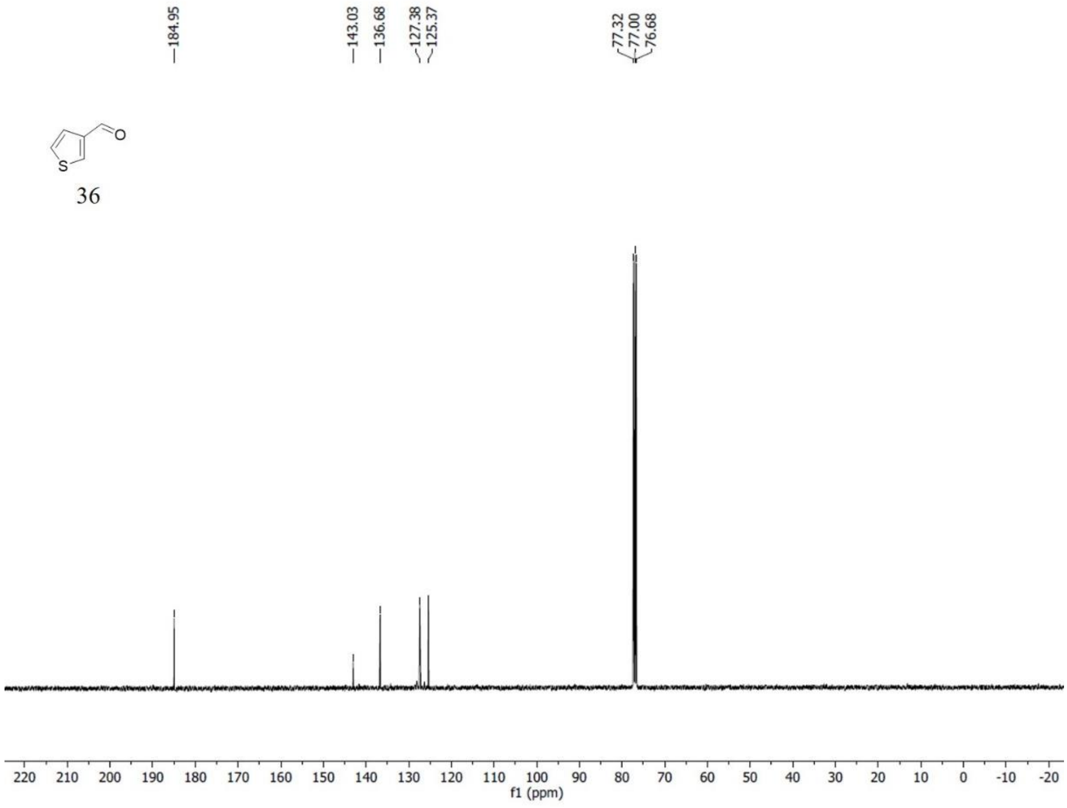

^{13}C NMR (101 MHz, $CDCl_3$)

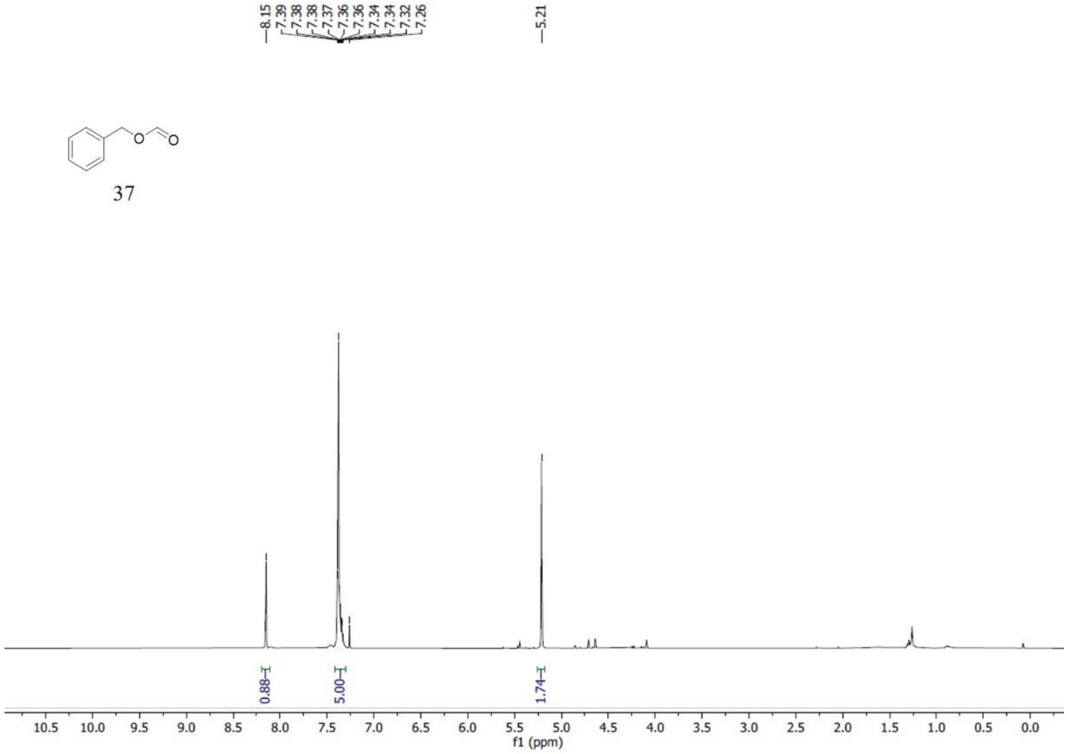

^{1}H NMR (400 MHz, $CDCl_3$)

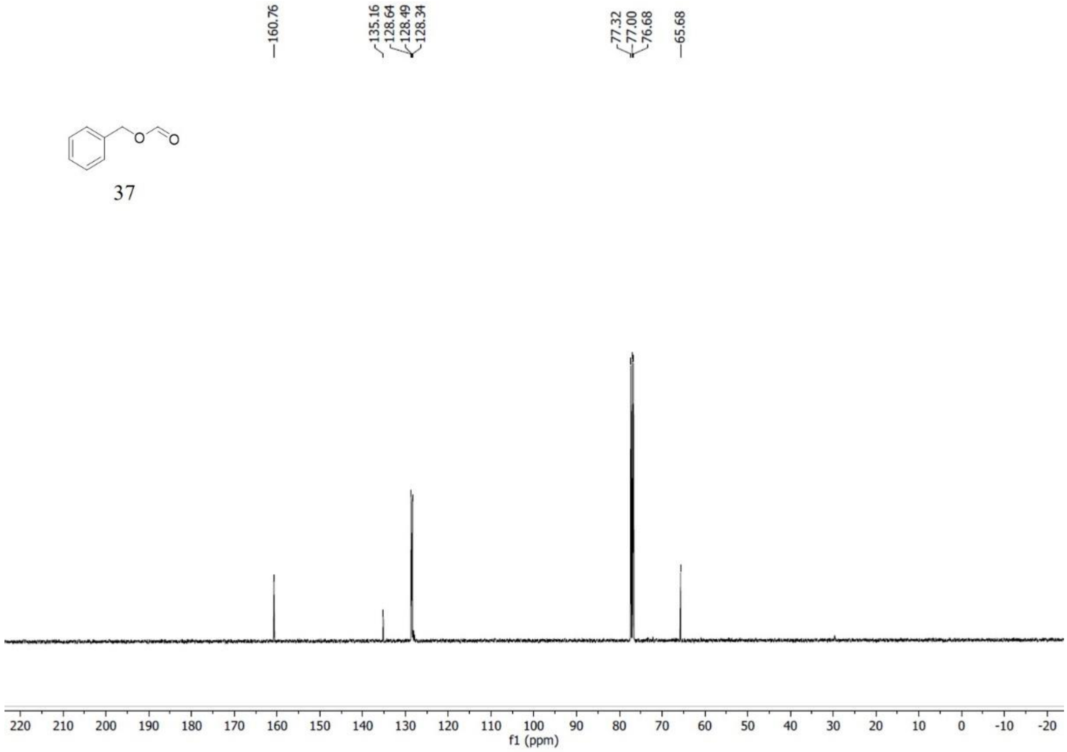

^{13}C NMR (101 MHz, $CDCl_3$)

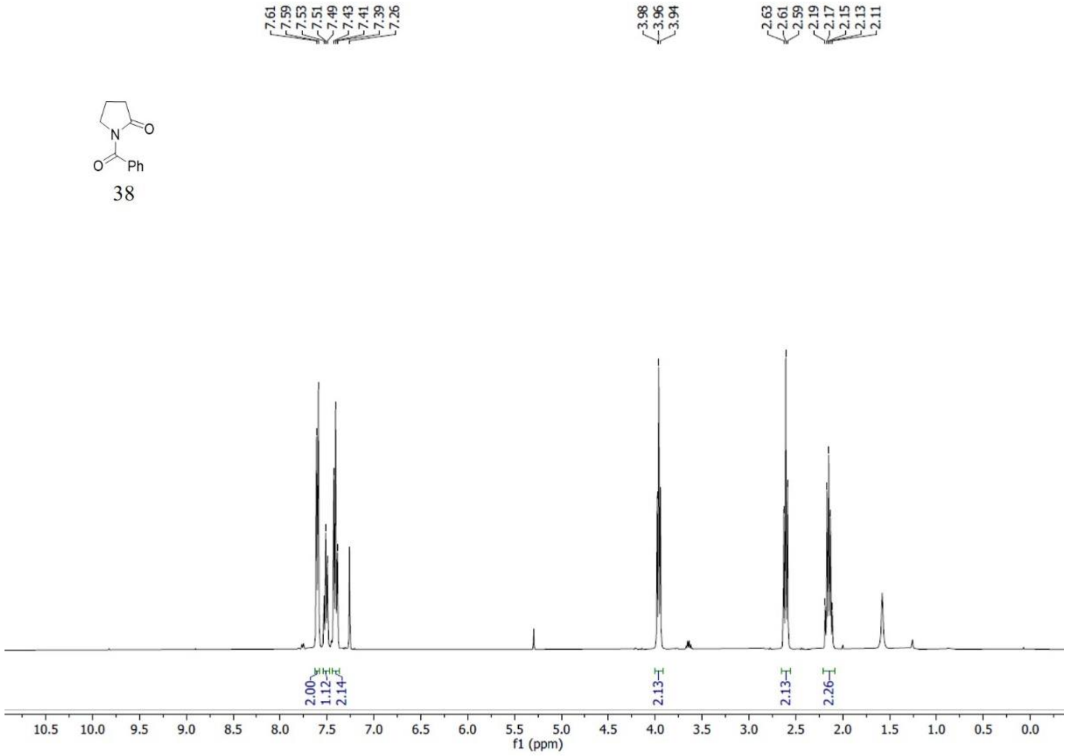

^1H NMR (400 MHz, $CDCl_3$)

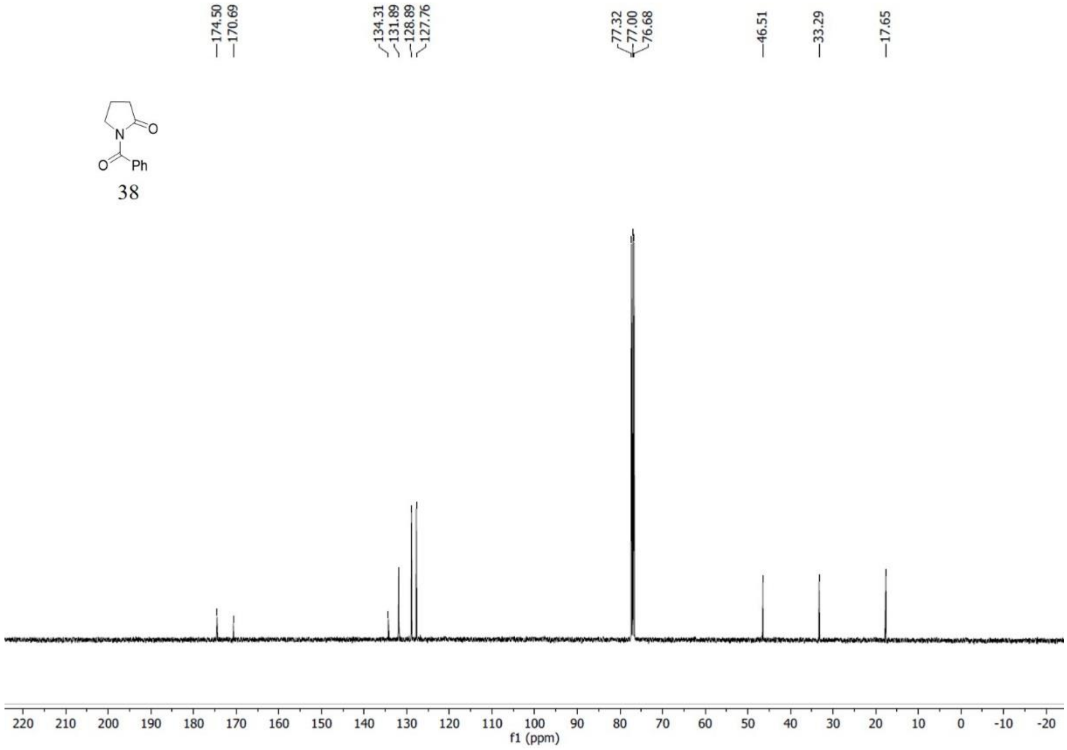

^{13}C NMR (101 MHz, $CDCl_3$)

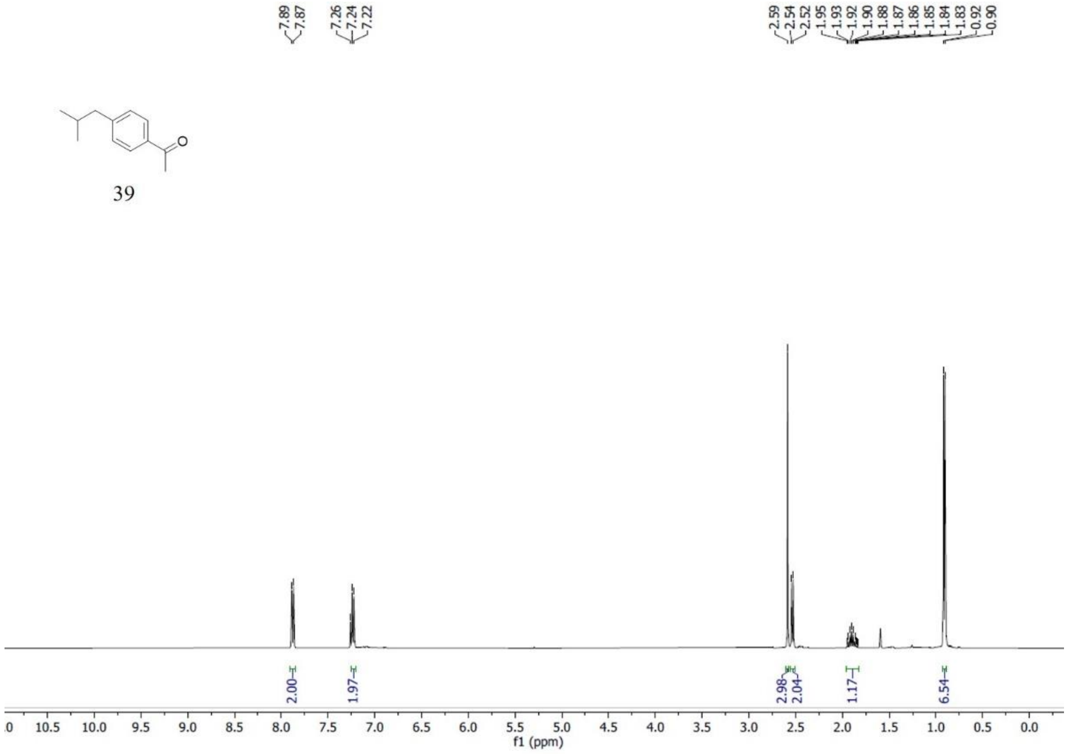

^{1}H NMR (400 MHz, $CDCl_3$)

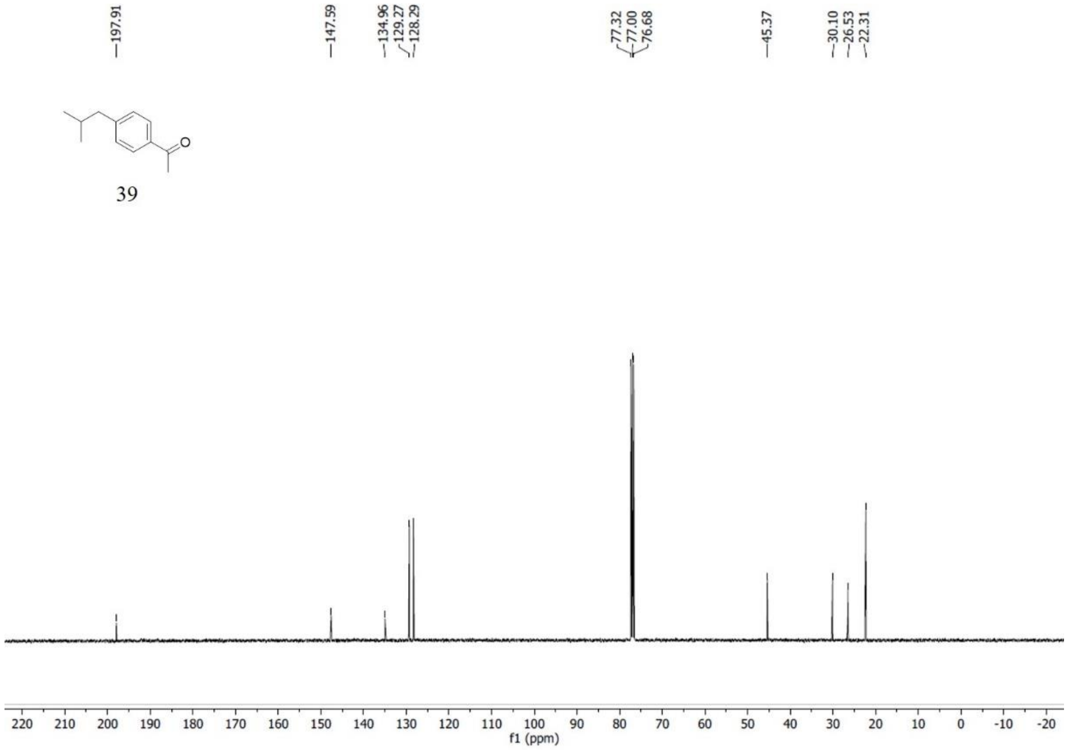

^{13}C NMR (101 MHz, $CDCl_3$)

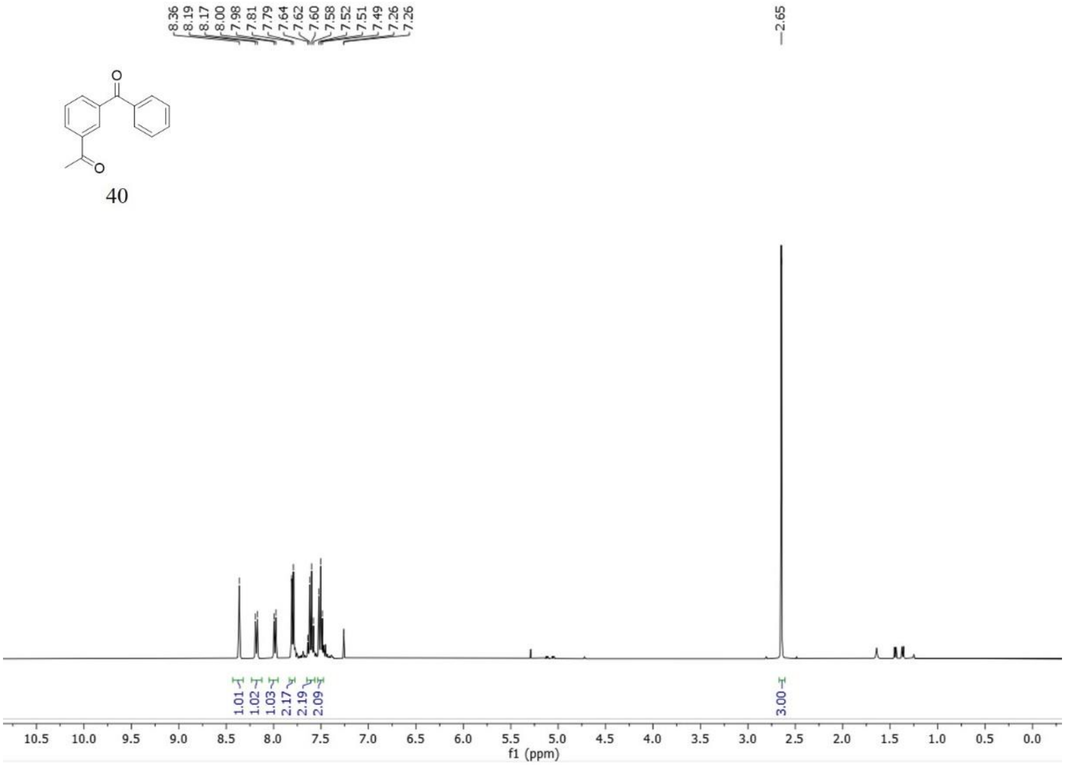

^{1}H NMR (400 MHz, $CDCl_3$)

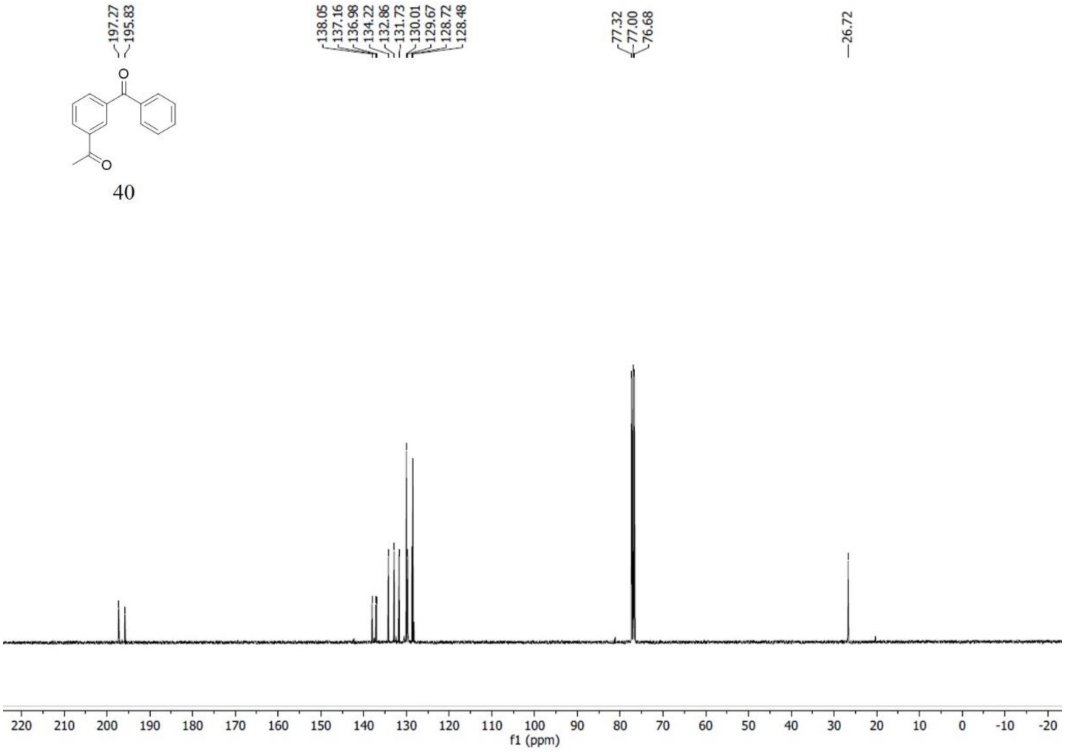

^{13}C NMR (101 MHz, $CDCl_3$)

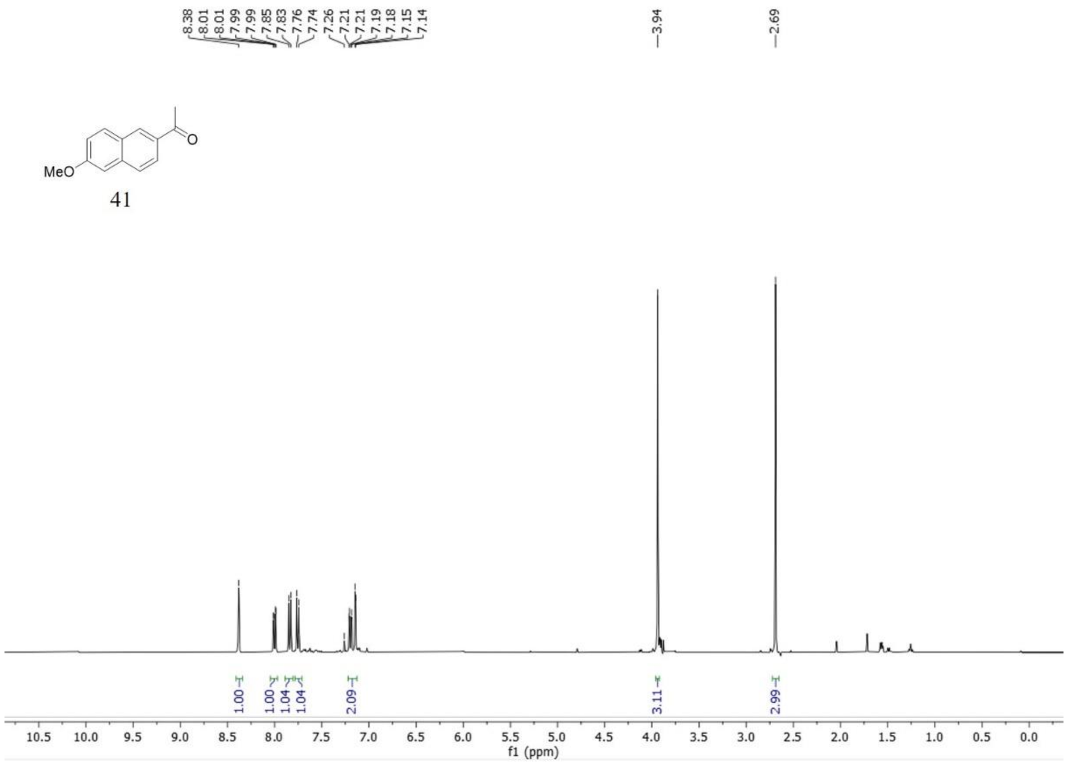

41

^{1}H NMR (400 MHz, $CDCl_3$)

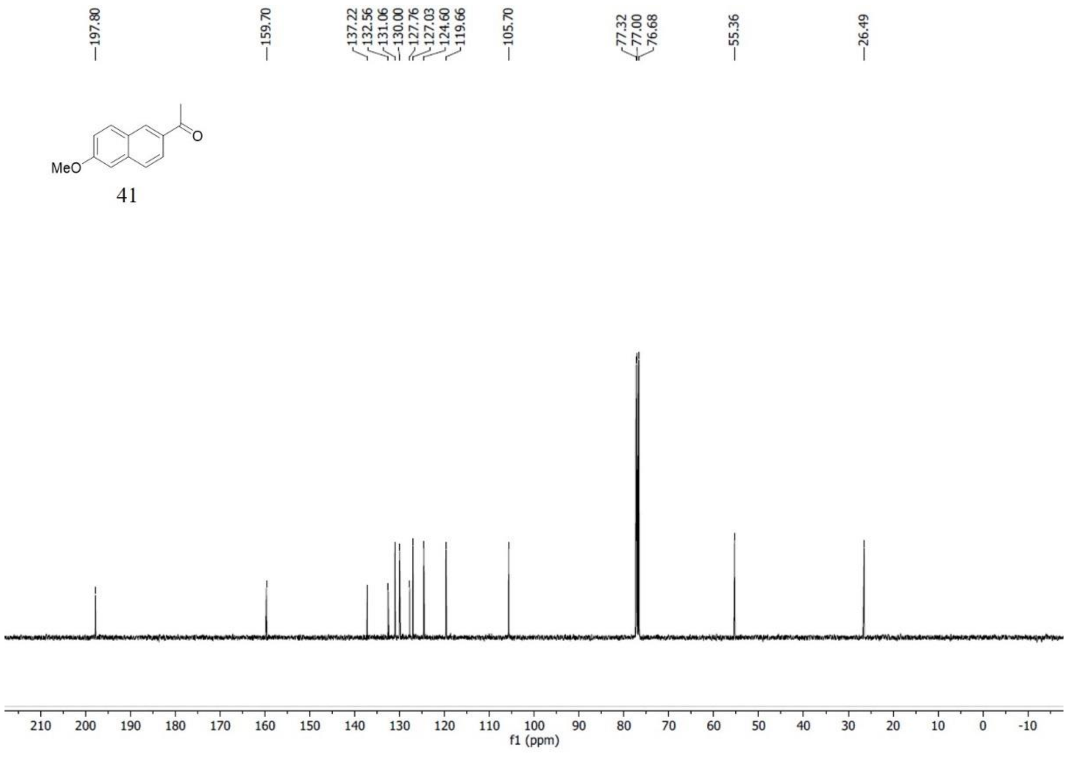

41

^{13}C NMR (101 MHz, $CDCl_3$)

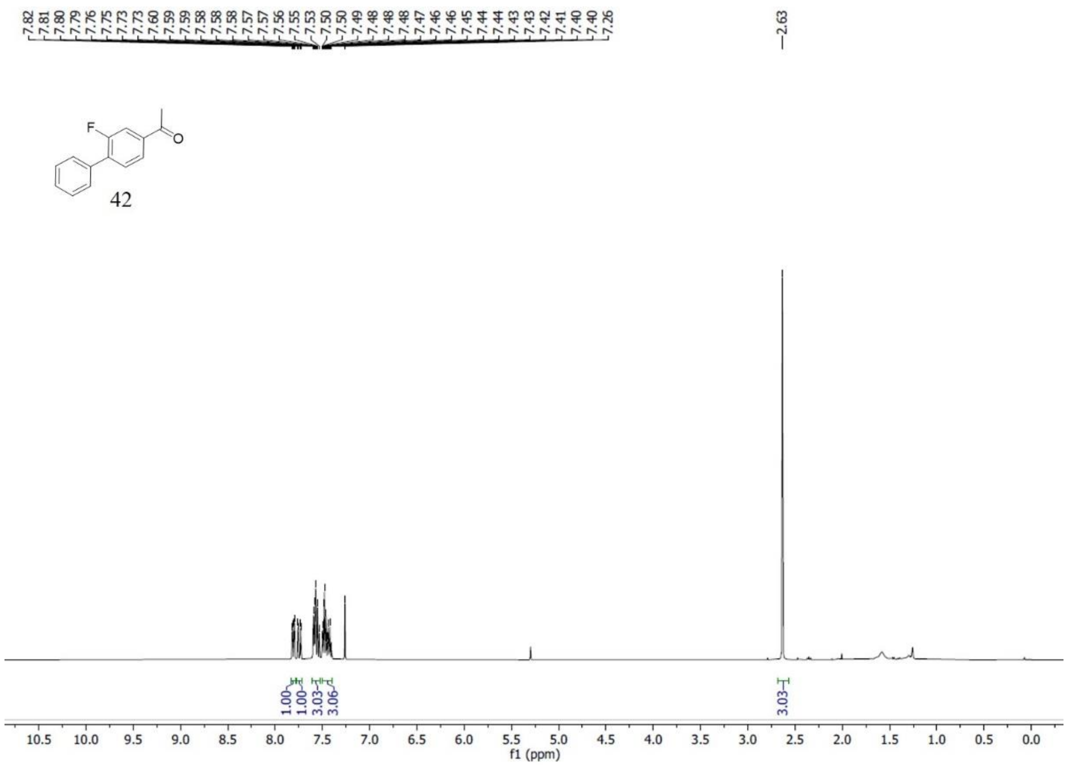

^{1}H NMR (400 MHz, $CDCl_3$)

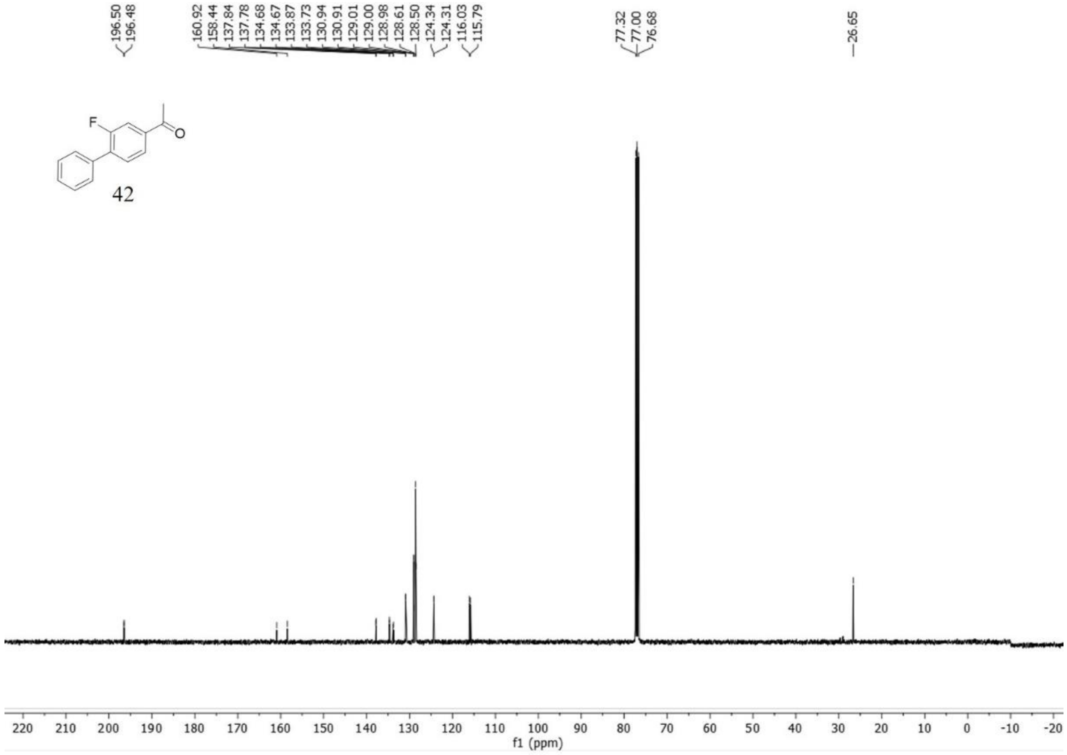

^{13}C NMR (101 MHz, $CDCl_3$)

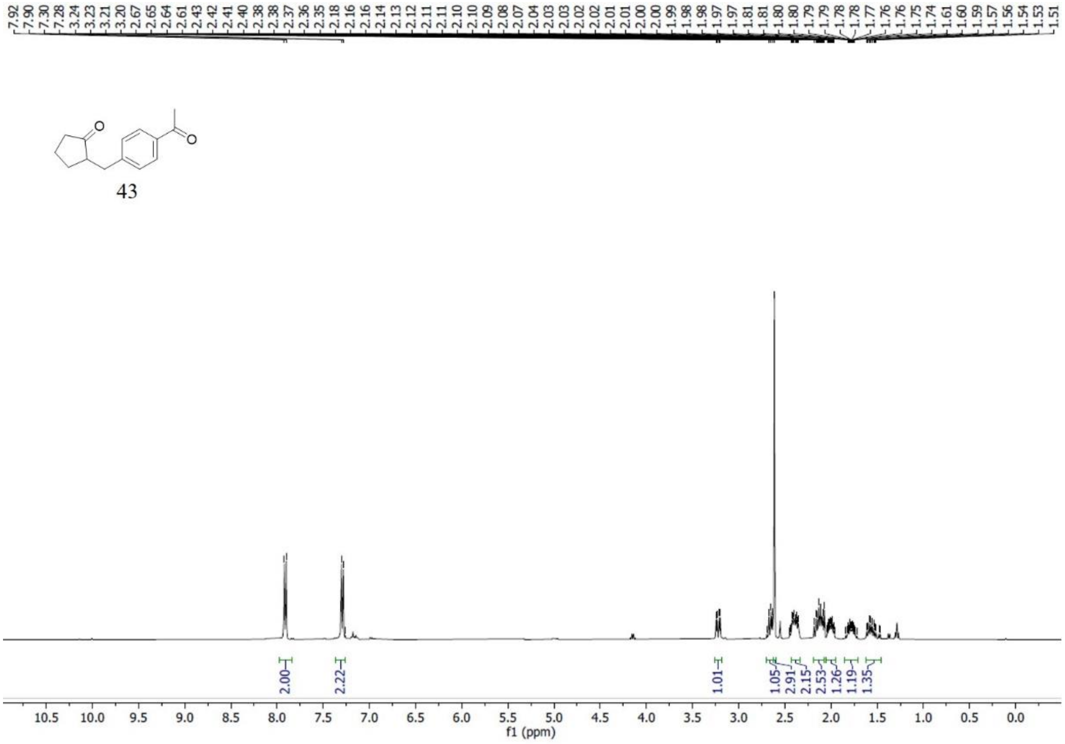

^{1}H NMR (400 MHz, $CDCl_3$)

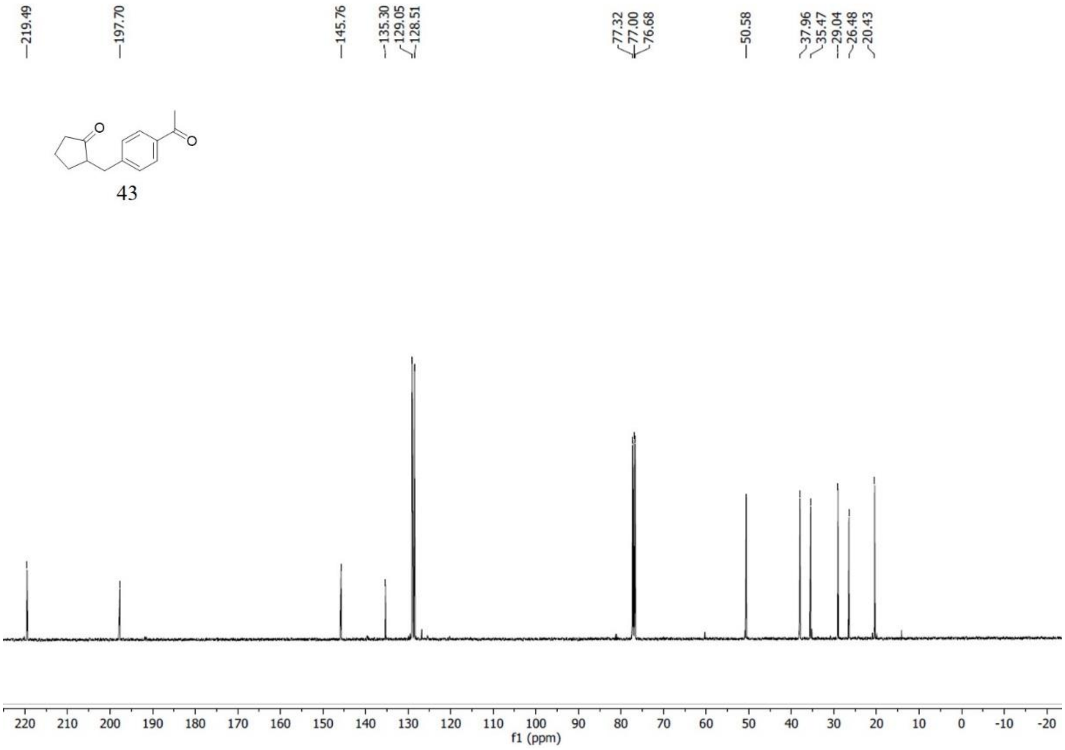

^{13}C NMR (101 MHz, $CDCl_3$)

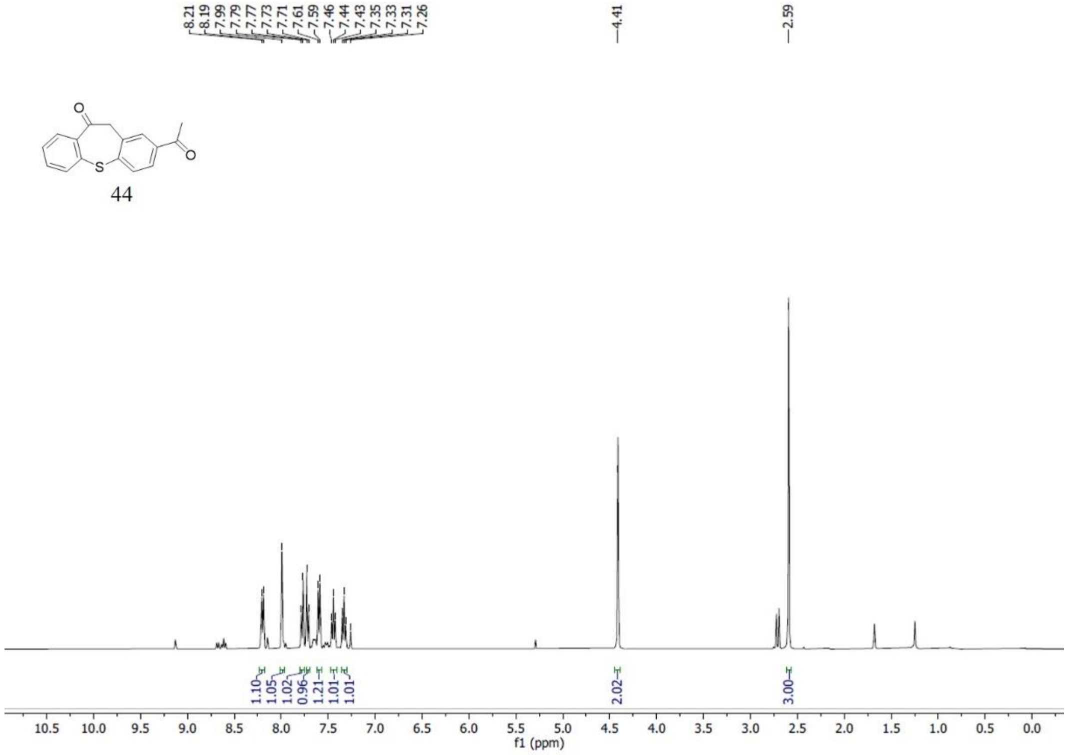

^{1}H NMR (400 MHz, $CDCl_3$)

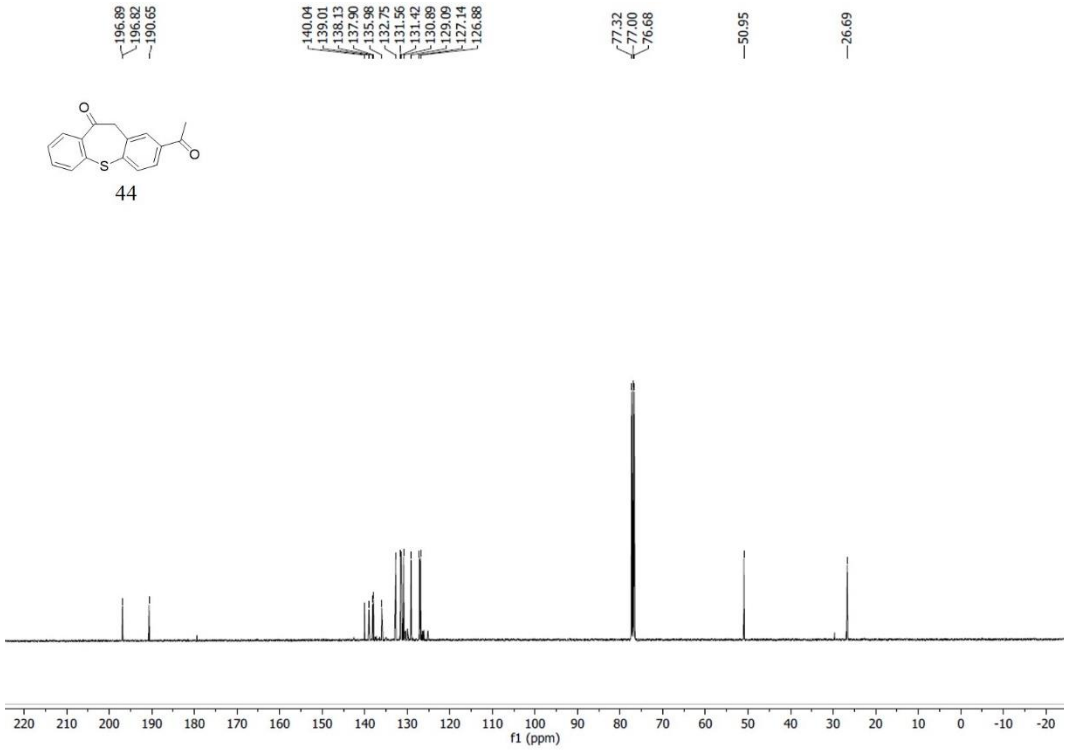

^{13}C NMR (101 MHz, $CDCl_3$)

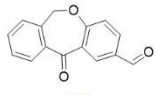

45

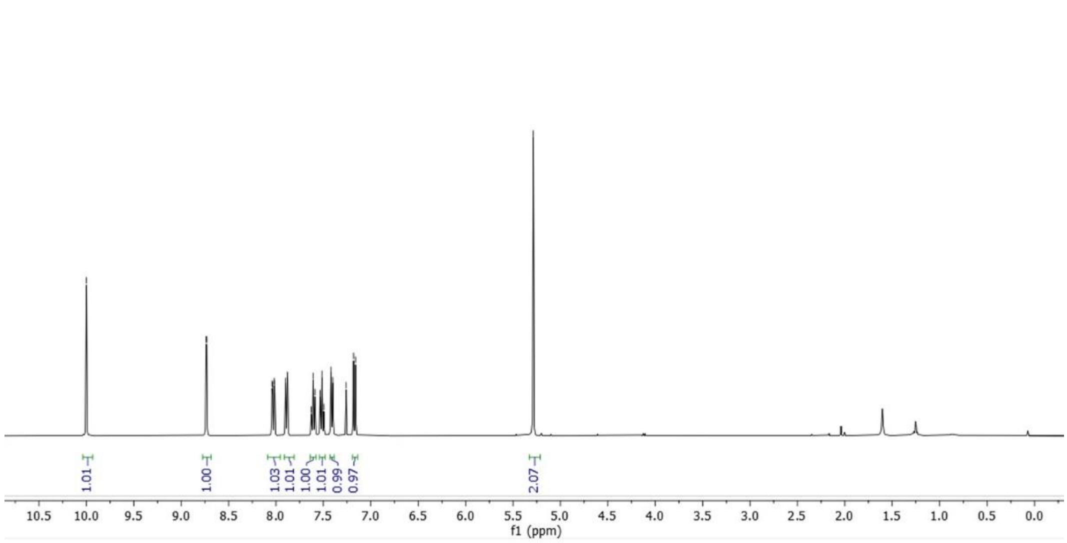

^{1}H NMR (400 MHz, $CDCl_3$)

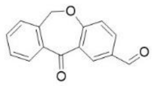

45

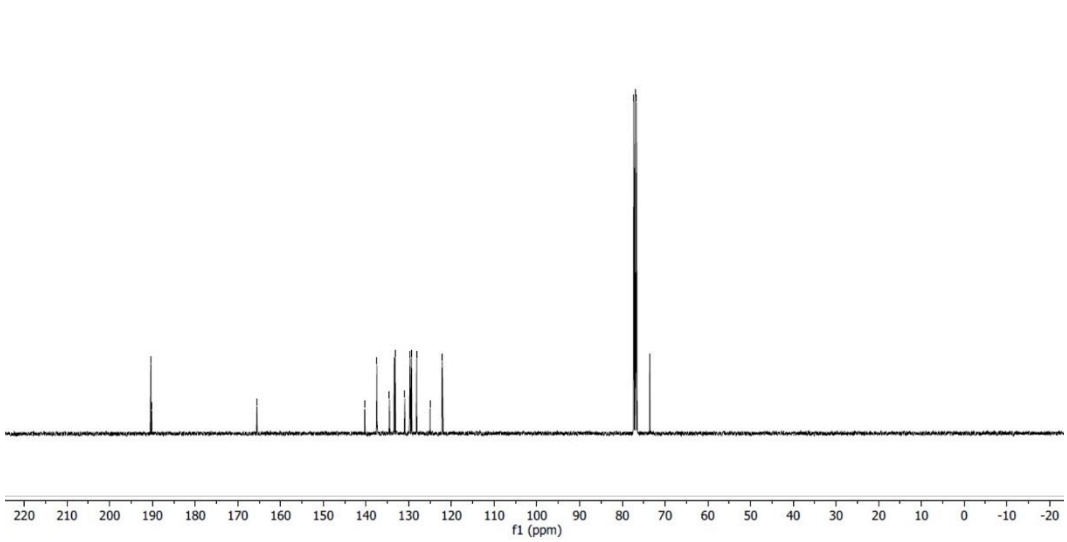

^{13}C NMR (101 MHz, $CDCl_3$)

References

[1] Y. Liao, Y. Zhou, Z. Zhang, J. Fan, F. Liu, Z. Shi, *Org. Lett.* **2021**, *23*, 1251-1257.
[2] J.-P. Bachmann, *U.S. Patent No. 9,745,245.* **2017**.
[3] Y. Sasano, S. Nagasawa, M. Yamazaki, M. Shibuya, J. Park, Y. Iwabuchi, *Angew. Chem. Int. Ed.* **2014**, *53*, 3236-3240.
[4] J. Stahl, V. R. Yatham, S. Crespi, B. König, *ChemistrySelect* **2021**, *6*, 4107-4111.
[5] M. E. Azenha, H. D. Burrows, S. M. Fonseca, M. L. Ramos, J. Rovisco, J. S. de Melo, A. J. F. N. Sobral, K. Kogej, *New J. Chem.* **2008**, *32*.
[6] K. Sugamoto, Y.-i. Matsushita, T. Matsui, *J. Chem. Soc. Perkin Trans. 1* **1998**, 3989-3998.
[7] Y. Hou, J. Hu, R. Xu, S. Pan, X. Zeng, G. Zhong, *Org. Lett.* **2019**, *21*, 4428-4432.
[8] S. Kyasa, R. N. Meier, R. A. Pardini, T. K. Truttmann, K. T. Kuwata, P. H. Dussault, *J. Org. Chem.* **2015**, *80*, 12100-12114.
[9] Y. Wang, P. Li, J. Wang, Z. Liu, Y. Wang, Y. Lu, Y. Liu, L. Duan, W. Li, S. Sarina, H. Zhu, J. Liu, *Catal. Sci. Technol.* **2021**, *11*, 4429-4438.
[10] T. Cui, L. Ma, S. Wang, C. Ye, X. Liang, Z. Zhang, G. Meng, L. Zheng, H. S. Hu, J. Zhang, H. Duan, D. Wang, Y. Li, *J. Am. Chem. Soc.* **2021**, *143*, 9429-9439.
[11] S.-S. Meng, L.-R. Lin, X. Luo, H.-J. Lv, J.-L. Zhao, A. S. C. Chan, *Green Chem.* **2019**, *21*, 6187-6193.
[12] M. Uyanik, R. Fukatsu, K. Ishihara, *Chem. Asian J.* **2010**, *5*, 456-460.
[13] J. Chen, X. Wang, X. Zheng, J. Ding, M. Liu, H. Wu, *Tetrahedron* **2012**, *68*, 8905-8907.
[14] C. Gennari, S. Ceccarelli, U. Piarulli, K. Aboutayab, M. Donghi, I. Paterson, *Tetrahedron* **1998**, *54*, 14999-15016.
[15] M. Uygur, J. H. Kuhlmann, M. C. Pérez-Aguilar, D. G. Piekarski, O. García Mancheño, *Green Chem.* **2021**, *23*, 3392-3399.
[16] P. K. Behera, P. Choudhury, S. K. Sahu, R. R. Sahu, A. N. Harvat, C. McNulty, A. Stitgen, J. Scanlon, M. Kar, L. Rout, *Asian J. Org. Chem.* **2021**, *10*, 1117-1122.
[17] P. D. Dharpure, A. Bhowmick, P. K. Warghude, R. G. Bhat, *Tetrahedron Lett.* **2020**, *61*.
[18] Z. Li, J. Chu, D. Meng, Y. Wen, X. Xing, H. Miao, M. Hu, C. Yu, Z. Wei, Y. Yang, Y. Li, *ACS Catal.* **2019**, *9*, 8659-8668.
[19] P. S. Humphries, R. Bersot, J. Kincaid, E. Mabery, K. McCluskie, T. Park, T. Renner, E. Riegler, T. Steinfeld, E. D. Turtle, Z. L. Wei, E. Willis, *Bioorg. Med. Chem. Lett.* **2018**, *28*, 293-297.
[20] C. Gangadurai, G. T. Illa, D. S. Reddy, *Org. Biomol. Chem.* **2020**, *18*, 8459-8466.
[21] Y. Sakakibara, P. Cooper, K. Murakami, K. Itami, *Chem. Asian J.* **2018**, *13*, 2410-2413.
[22] N. Kawai, N. Kato, Y. Hamada, T. Shioiri, *Chem. Pharm. Bull.* **1983**, *31*, 3139-3148.
[23] L. Ge, D. X. Wang, R. Xing, D. Ma, P. J. Walsh, C. Feng, *Nat. Commun.* **2019**, *10*, 4367.
